# The Rvv two-component regulatory system regulates biofilm formation and colonization in *Vibrio cholerae*

**DOI:** 10.1371/journal.ppat.1011415

**Published:** 2023-05-22

**Authors:** Giordan Kitts, Andrew Rogers, Jennifer K. Teschler, Jin Hwan Park, Michael A. Trebino, Issac Chaudry, Ivan Erill, Fitnat H. Yildiz

**Affiliations:** 1 Department of Microbiology and Environmental Toxicology, University of California, Santa Cruz, Santa Cruz, California, United States of America; 2 Department of Biological Sciences, University of Maryland Baltimore County (UMBC), Baltimore, Maryland, United States of America; University of California Davis School of Medicine, UNITED STATES

## Abstract

The facultative human pathogen, *Vibrio cholerae*, employs two-component signal transduction systems (TCS) to sense and respond to environmental signals encountered during its infection cycle. TCSs consist of a sensor histidine kinase (HK) and a response regulator (RR); the *V*. *cholerae* genome encodes 43 HKs and 49 RRs, of which 25 are predicted to be cognate pairs. Using deletion mutants of each HK gene, we analyzed the transcription of *vpsL*, a biofilm gene required for *Vibrio* polysaccharide and biofilm formation. We found that a *V*. *cholerae* TCS that had not been studied before, now termed Rvv, controls biofilm gene transcription. The Rvv TCS is part of a three-gene operon that is present in 30% of Vibrionales species. The *rvv* operon encodes RvvA, the HK; RvvB, the cognate RR; and RvvC, a protein of unknown function. Deletion of *rvvA* increased transcription of biofilm genes and altered biofilm formation, while deletion of *rvvB* or *rvvC* lead to no changes in biofilm gene transcription. The phenotypes observed in Δ*rvvA* depend on RvvB. Mutating RvvB to mimic constitutively active and inactive versions of the RR only impacted phenotypes in the Δ*rvvA* genetic background. Mutating the conserved residue required for kinase activity in RvvA did not affect phenotypes, whereas mutation of the conserved residue required for phosphatase activity mimicked the phenotype of the *rvvA* mutant. Furthermore, Δ*rvvA* displayed a significant colonization defect which was dependent on RvvB and RvvB phosphorylation state, but not on VPS production. We found that RvvA’s phosphatase activity regulates biofilm gene transcription, biofilm formation, and colonization phenotypes. This is the first systematic analysis of the role of *V*. *cholerae* HKs in biofilm gene transcription and resulted in the identification of a new regulator of biofilm formation and virulence, advancing our understanding of the role TCSs play in regulating these critical cellular processes in *V*. *cholerae*.

## Author summary

Two-component signal transduction systems are one of the most used regulatory networks by microorganisms to sense, respond, and adapt to their environments and increase their environmental fitness. We systematically analyzed *V*. *cholerae* HKs and identified Rvv, a new regulator of biofilm gene transcription and virulence. This study lays the groundwork for understanding the signal transduction pathways that regulate *V*. *cholerae’s* environmental fitness. Additionally, it enhances our understanding of the role TCSs play in regulating these critical cellular processes in *V*. *cholerae*.

## Introduction

Two-component signal transduction systems (TCSs), composed typically of a sensor histidine kinase and a cognate response regulator, are one of the most prevalent regulatory mechanisms that allow microorganisms to detect, respond to, and adapt to changes in their extracellular or intracellular environments [1,2]. In the most common type of two-component system, a cell membrane-associated sensor protein called a histidine kinase (HK) phosphorylates itself on a conserved histidine residue when it senses a change in the environment [3]. A phosphoryl group is then transferred to a cytoplasmic response regulator (RR), and phosphorylation of the RR’s conserved aspartate residue activates its output domain. While there are many kinds of RR output domains, most of them are DNA-binding domains that link phosphorylation to the transcriptional regulation of target genes. Many pathogenic microorganisms use TCSs to modulate gene transcription in response to changes in their environment and regulate diverse virulence-associated cellular processes [4].

*Vibrio cholerae*, a facultative human pathogen responsible for the life-threatening diarrheal disease cholera, uses TCSs to enhance its environmental fitness during the infection cycle. The genome of the *V*. *cholerae* O1 El Tor N16961 strain encodes 43 histidine kinases (HKs) and 49 response regulators (RRs) [5]. Specifically, *V*. *cholerae* has 29 classical types HKs (HisKA and HATPase domains), six hybrid HKs (HisKA, HATPase, and REC domains), five unorthodox HKs (HisKA, HATPase, REC, and HPT domains), and three CheA type HKs (HPT, H-kinase_dim, HATPase_c, and CheW domains) [5].

A set of *V*. *cholerae* TCSs is known to regulate *V*. *cholerae* pathogenesis; these include VarAS, the quorum sensing (QS) signal transduction circuitry (CqsS, LuxPQ, CqsR, VpsS kinases through LuxO), VieABS, PhoBR, and ArcA [6–12]. They regulate the production of major virulence factors, the toxin coregulated pilus (TCP), and the cholera toxin (CT) through their regulation of the virulence network master transcriptional regulators ToxT, ToxRS, and TcpP [10,13–15]. Another group of TCSs impacts intestinal colonization through their ability to control motility and chemotaxis (FlrBC and CheY-3), resistance to antimicrobial peptides (CarRS), colonization resistance by interbacterial competition (VxrABC), or increasing growth advantage through modulation of the acetate switch (CrbRS) [16–23].

In *V*. *cholerae*, TCSs also govern the formation and dispersal of biofilms [24]. Biofilms enhance the pathogen’s environmental survival, transmission, and infectivity to the human host. Biofilm formation is tightly regulated by diverse physical, chemical, and biological cues [25,26]. *V*. *cholerae* biofilm formation requires production of the main matrix component, *Vibrio* polysaccharide (VPS) [27]. The genes encoding the proteins for VPS production reside on two operons, *vps*-I, and *vps-II* [27]. The *vps* operons are under the control of multiple regulators. Two orphan response regulators, VpsR and VpsT, activate the transcription of *vps* operons upon binding to the signaling molecule c-di-GMP [28–31]. The quorum sensing (QS) phosphorelay signal transduction circuitry also controls the *vps* operons. CqsS, LuxPQ, CqsR, and VpsS kinases, through the signal integrator protein LuxU, direct phosphate to the response regulator LuxO [8,9]. At low cell densities, phospho-LuxO stimulates the transcription of genes encoding the Qrr sRNA regulatory RNAs [6]. The Qrr sRNAs repress the translation of HapR, which directly represses the transcription of biofilm genes [32]. The VxrABCDE system positively regulates *vps* gene transcription, partly by integrating envelope stress into biofilm formation [33,34]. Several TCSs, including PhoBR, CarRS, NtrBC, VieABS, and DbfSR, repress biofilm formation [20,35–39]. These RRs may affect the formation of biofilms by interacting with other important regulators or by controlling the transcription of biofilm genes directly.

In earlier studies, we systematically evaluated the involvement of *V*. *cholerae* TCSs in intestinal colonization and biofilm formation by generating in-frame deletion mutants of each RR gene and studying the mutants for their *in vivo* colonization and biofilm gene transcription phenotypes [22,33]. We wanted to determine how a similar study of HK genes would compare with this RR work. In-frame deletion strains for each of the 42 genes encoding HKs were generated. We performed phenotypic characterization of this deletion library by looking at how each *V*. *cholerae* HK affected the transcription of biofilm genes. We identified a previously uncharacterized HK, VCA0257, which we named RvvA (regulator of *V**ibrio* *v**ps*), which regulates *vps* gene transcription and biofilm formation as well as *in vivo* colonization. We further show that the Rvv TCS operates by conventional phosphorylation-dependent signal transduction.

## Results

### VCA0257/RvvA regulates *vps* gene transcription

**Fig 1:**
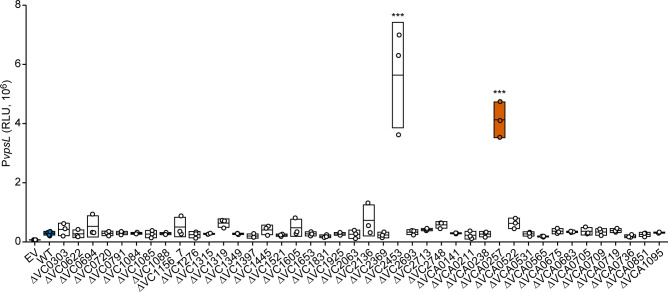
Identification of HKs impacting *vpsL* transcription in *Vibrio cholerae*. Promoter activity of the transcriptional fusion P*vpsL-lux* was measured from cells grown to exponential phase in strains harboring deletions of individual HKs and WT. The orange coloring indicates the HK (VCA0257) that is the focus of this study. Individual data points of Relative Luminescent Units (RLU) are plotted with bars at the mean and error bars representing standard deviation. Statistical significance was determined using a One-Way ANOVA and post-hoc Tukey’s multiple comparisons test. Means from individual biological replicates (n ≥ 3) were compared to that of wild type, and differences with an adjusted *P* value of ≤ 0.01 were deemed significant. ***, *P* ≤ 0.0001.

There has been no systematic examination of the effect of HKs on *V*. *cholerae* biofilm gene transcription. To evaluate the role of the *V*. *cholerae* HKs in biofilm formation, we first generated in-frame deletion mutants for each of the 42 predicted HKs. We excluded VC1639 as we could not mutate this gene in the *V*. *cholerae* strain used here, A1552. Next, to identify the specific HKs that contribute to *vps* transcription, we measured the promoter activity of the *vps-II* operon genes under standard laboratory conditions as a proxy for biofilm gene transcription. We compared the transcriptional activity of the *PvpsL-lux* reporter in wild-type *V*. *cholerae* and the HK in-frame deletion strains. Most mutants did not exhibit any difference in *vpsL* transcription when compared to the wild type (Fig 1). Five, however, resulted in decreased *vpsL* transcription: VCA0565-(*vxrA*), VC1831-(*cqsR*), VCA0736-(*luxQ*), and VC1397-(*cheA1*). In contrast, deletion of nine increased *vpsL* transcription: VC1605, VC1156-7, VC0694, VC2748-(*ntrB*), VCA0522-(*cqsS*), VC1319-(*carS*), VC2136-(*flrB*), VCA0257, and VC2453-(*varS*). Only four of these have not been identified in previous work: VC1605, VC1156-7, VC0694, and VCA0257. In this study, we focused on VCA0257 (RvvA), because it was uncharacterized and its deletion resulted in the second highest increase in *vpsL* transcription (Fig 1).

### *rvv* TCS regulates *vps* gene transcription

**Fig 2:**
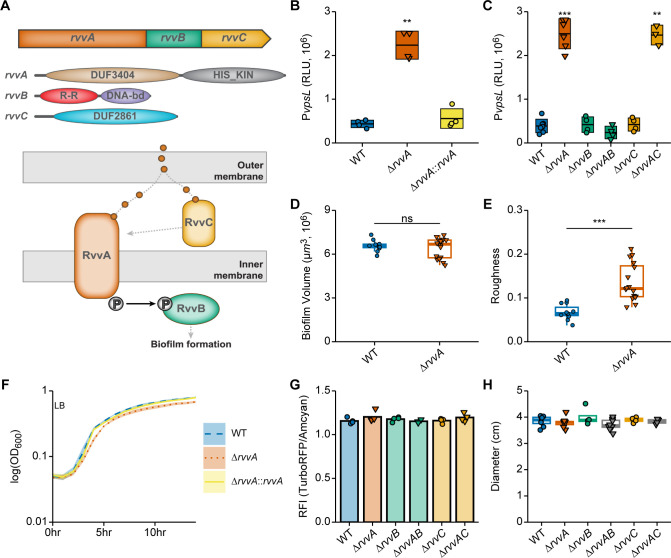
Rvv contributions to biofilm formation and biofilm-associated cellular processes in *V*. *cholerae*. (A) Depiction of the genomic region surrounding *rvvA* (VCA0257) (top), the predicted domains of each protein in RvvABC (middle), and putative model of Rvv interactions based on predicted function and cellular localization (bottom). (B–C) Promoter activity of the transcriptional fusion P*vpsL*-lux was measured from cells grown to exponential phase in the indicated strains. Individual data points (circles–RvvA present; triangles–RvvA deleted) of Relative Luminescent Units (RLU) are plotted with crossbars representing mean and standard deviation. Statistical significance was determined using a One-Way ANOVA and post-hoc Tukey’s multiple comparisons test. Means from individual biological replicates (n ≥ 3) were compared to that of wild type, and differences with an adjusted *P* value of ≤ 0.01 were deemed significant. **, *P* ≤ 0.001; ***, *P* ≤ 0.0001. (D-E) Static biofilms of *gfp*-tagged WT and Δ*rvvA* strains were grown in LB for 6 hours at 30°C and imaged with confocal laser scanning microscopy (CLSM). Biofilm biovolume (D) and biofilm roughness (E) of WT and Δ*rvvA* static biofilms was measured using the quantitative image analysis software BiofilmQ. Means from at least three biological replicates were compared by an unpaired t-test, and mean differences with a *P* value of ≤ 0.01 were considered significant. ***, P ≤ 0.001; ns, not significant. (F) Growth curves of WT (blue), Δ*rvvA* (orange), and the complemented *rvvA* deletion strain Δ*rvvA*::*rvvA* (yellow) in LB medium, 30°C. (G) c-di-GMP levels were measured from cells grown to exponential phase in the indicated strains using the Bc3-4 c-di-GMP biosensor. Relative Fluorescence Intensity (RFI) was measured as TurboRFP / Amcyan (normalizer). (H) Single colonies of indicated strains were inoculated in 0.3% agar plates. Swimming motility was measured by diameter (cm) surrounding inoculum position after 16 hours of incubation. n ≥ 4.

Analysis of the *V*. *cholerae rvvA* genomic context showed that VCA0257 (*rvvA*) is the first gene of a predicted three-gene operon composed of VCA0257, VCA0256 (*rvvB*), and VCA0255 (*rvvC*) (Fig 2A). RvvA is a HisKA family protein; it is 484 amino acids in length and it is predicted to have an N-terminal DUF3404 domain (25–248), a transmembrane region (249–271), and a C-terminal histidine kinase domain (272–484). The DUF3404 domain is functionally uncharacterized and is found associated with proteins containing Pfam domains PF02518 (HATPase_c) and PF00512 (HisKA). The RvvB protein is 223 amino acids in length with an N-terminal REC domain (10–121) and a C-terminal winged helix-turn-helix DNA-binding domain (126–220) (OmpR/PhoB-type-DNA-binding domain). The RvvC protein is 276 amino acids in length; it is predicted to be a periplasmic protein containing a DUF2861 domain (36–276). The DUF2861 domain is functionally uncharacterized and proteins containing this domain have no known function.

We next determined the role of the *rvv* loci in *vps* gene transcription. We found that *vpsL* promoter activity, measured using P*vpsL-lux* in the exponentially grown Δ*rvvA* strain, increased 5-fold compared to the wild type. This phenotype is complemented by introducing the wild-type copy of *rvvA* under the control of its native promoter in the Tn7 site (Fig 2B). These results demonstrate that RvvA negatively regulates *vpsL* gene transcription under the conditions tested. To determine whether the products of *rvvB* or *rvvC* are involved in this regulation, we generated in-frame deletion mutants of *rvvB* or *rvvC* and analyzed *vpsL* transcription in these strains. We observed that, in contrast to Δ*rvvA*, Δ*rvvB* and Δ*rvvC* strains showed no changes in *vpsL* transcription. We asked if the elevated *vpsL* transcription in Δ*rvvA* depended on RvvB or RvvC; we determined that while the Δ*rvvAB* strain has *vpsL* transcription similar to wild type, Δ*rvvAC* displayed increased *vpsL* transcription similar to Δ*rvvA* (Fig 2C). This finding suggests that, under the conditions tested, the increase in *vpsL* transcription seen in Δ*rvvA* requires RvvB but does not require RvvC.

We then compared biofilm formation between wild-type and Δ*rvvA* strains. Biofilms were grown under static conditions and quantified using BiofilmQ to calculate biofilm volume and roughness [40] (Fig 2D–2E). Overall, under the conditions tested, the total biomass was similar between wild type and Δ*rvvA*, but biofilm roughness in Δ*rvvA* was higher than that of the wild type. Our results show that RvvA alters the structural properties of biofilms, albeit slightly. We also compared the growth of Δ*rvvA*, a *rvvA* complemented strain, and the wild type (Fig 2F). While the overall growth patterns of the strains were similar, we found that the generation time of the Δ*rvvA* (67.4min ± 1.7) was slightly increased compared to the wild type (63.6min ± 0.8).

Biofilm formation and motility are inversely regulated, and cellular levels of the signaling molecule cyclic-di-GMP govern both processes. To see if Rvv influenced motility and cellular c-di-GMP levels, we measured swimming motility diameter and cellular c-di-GMP levels in *rvv* loci deletion strains using a c-di-GMP reporter. In contrast to *vpsL* promoter activity, the Δ*rvv* strains showed no differences from the wild type in cellular c-di-GMP levels or motility (Fig 2G and 2H). Taken together, these results indicate that RvvA regulates *vps* gene transcription independently of c-di-GMP signaling.

### RvvA acts upstream of the major biofilm regulators, VpsR and VpsT

**Fig 3:**
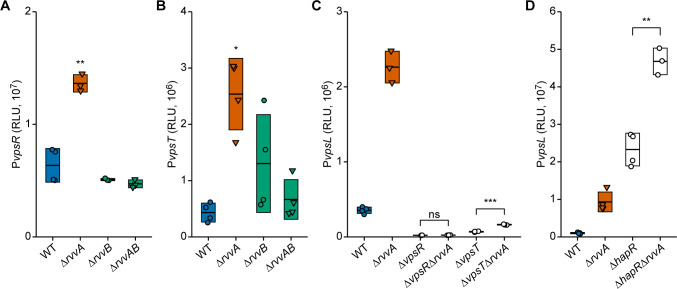
Epistasis analysis of *rvv* loci contributions to biofilm formation. Promoter activity of the transcriptional fusions P*vpsR*-lux (A), and P*vpsT*-lux (B) P*vpsL*-lux (C, D) were measured from cells grown to exponential phase in the indicated strains. Individual data points (circles–RvvA present; triangles–RvvA deleted) of Relative Luminescent Units (RLU) are plotted with crossbars representing mean and standard deviation. (A, B), Statistical significance was determined using a One-Way ANOVA and post-hoc Tukey’s multiple comparisons test. Means from individual biological replicates (n ≥ 3) were compared to that of wild type, and differences with an adjusted *P* value of ≤ 0.01 were deemed significant. **, *P* ≤ 0.001; ***, *P* ≤ 0.0001. (C, D) Means from individual biological replicates (n ≥ 3) were compared by an unpaired t-test, and mean differences with a *P* value of ≤ 0.01 were considered significant. **, *P* ≤ 0.001; ***, *P* ≤ 0.0001; ns, not significant.

*V*. *cholerae* core biofilm regulatory circuitry has two positive regulators, VpsR and VpsT, and a negative regulator, HapR. To gain further insight into the mechanism by which RvvA contributes to *vpsL* transcription, we investigated whether the absence of RvvA affects the transcription of *vpsR* and *vpsT*, which directly regulate *vps* gene transcription. We measured the promoter activity of the *vpsR* and *vpsT* genes using the transcriptional reporters P*vpsR*-*lux* and P*vpsT*-*lux* in wild-type, Δ*rvvA*, Δ*rvvB*, and Δ*rvvAB* strains. We observed that Δ*rvvA* has a 2-fold increase in *vpsR* and a 6-fold increase in *vpsT* transcription compared to the wild type (Fig 3A and 3B). In contrast, there was no statistically significant change in *vpsR* and *vpsT* transcription in either the Δ*rvvB* or Δ*rvvAB* strain. These findings suggest that RvvA regulates the transcription of *vps* structural and regulatory genes and that this regulation requires RvvB.

We conducted genetic interaction studies to determine how RvvA contributes to biofilm formation. To this end, we first generated *ΔrvvAΔvpsR* and *ΔrvvAΔvpsT* double mutants. We next measured P*vpsL*-*lux* promoter activity in *ΔrvvAΔvpsR* and *ΔrvvAΔvpsT*, *ΔrvvA*, *ΔvpsR*, and *ΔvpsT* strains (Fig 3C). In Δ*vpsRΔrvvA*, the *vpsL* transcription levels were similar to that of the Δ*vpsR*, suggesting RvvA requires VpsR for *vpsL* regulation. In *ΔrvvAΔvpsT*, *vpsL* transcription was 2-fold higher than in Δ*vpsT*, suggesting that VpsT contributes but is not required for RvvA effects. Together, these findings indicate that VpsR and VpsT function downstream of RvvA to control *vpsL* transcription.

Finally, we wanted to know if RvvA’s effect on *vpsL* transcription depended on HapR, the main negative regulator of biofilm formation (Fig 3D). Deletion of *hapR* led to a 23-fold increase in *vpsL* transcription compared to the wild type. In contrast, a Δ*hapR*Δ*rvvA* strain had a 51-fold increase in *vpsL* transcription compared to the wild type. The increase in *vpsL* transcription in Δ*hapR*Δ*rvvA* strains was greater than in Δ*hapR* or Δ*rvvA* strains, indicating that HapR and RvvA modulate *vpsL* transcription via parallel regulatory pathways.

### Mutations in the RvvA-HisKA and RvvB-REC domains impact *vps* gene transcription

**Fig 4:**
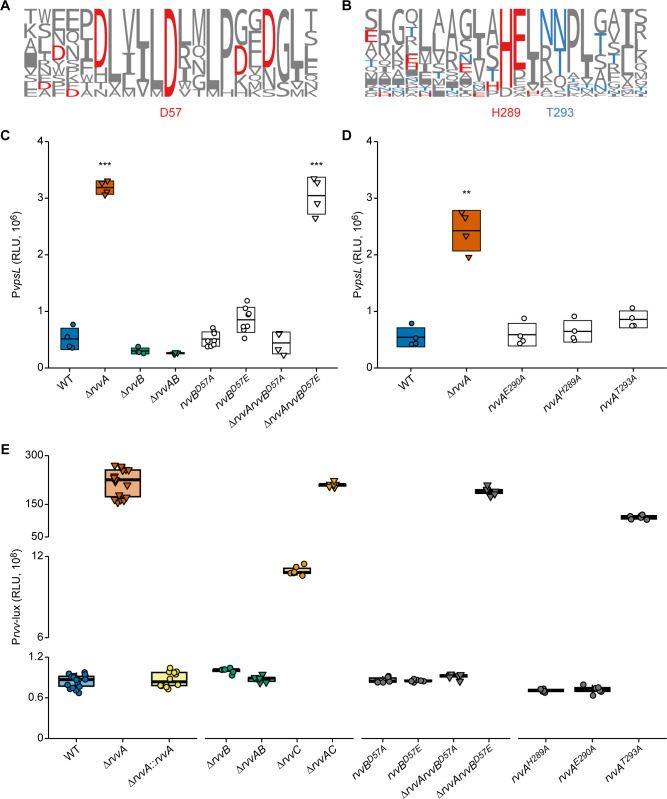
Impact of RvvAB phosphotransfer mutations on Rvv phenotypes. (A) Seqlogo generated from amino acid sequence alignment of the REC domain of OmpR-like response regulators (RRs) in *V*. *cholerae* using ClustalO. The most conserved aspartate that was chosen for mutation is labeled (D57). (B) Seqlogo from amino acid sequence alignment of the HisKA DHp domain of Classic-type histidine kinases (HKs) in *V*. *cholerae* using ClustalO. Labels indicate the mutations generated in residues expected to impact kinase (H289) and phosphatase (T293) activity, respectively. (C-D) Promoter activity of the transcriptional fusion P*vpsL*-lux was measured from cells grown to exponential phase in the indicated strains. Individual data points (circles–RvvA present; triangles–RvvA deleted) of Relative Luminescent Units (RLU) are plotted with crossbars representing mean and standard deviation. Statistical significance was determined using a One-Way ANOVA and post-hoc Tukey’s multiple comparisons test. Means from individual biological replicates (n ≥ 6) were compared to that of wild type, and differences with an adjusted *P* value of ≤ 0.01 were deemed significant. **, *P* ≤ 0.001; ***, *P* ≤ 0.0001. (E) Promoter activity of the transcriptional fusion P*rvv*-lux was measured from cells grown to exponential phase in the indicated strains. Individual data points (circles–RvvA present; triangles–RvvA deleted) of Relative Luminescent Units (RLU) are displayed on top of a boxplot for each strain.

The phosphorylation state of a RR determines its activity; we identified the aspartate residue that is predicted to be phosphorylated in the REC domain of RvvB using an amino acid sequence alignment of the *V*. *cholerae* OmpR family RRs (Figs 4A and S5A). We then substituted the aspartate residue in the REC domain of RvvB to mimic constitutively active (D57E) and inactive (D57A) versions and replaced the wild-type gene in the chromosome with the mutated versions of *rvvB* in both Δ*rvvA* and wild-type genetic backgrounds. We determined the impact of RvvB^D57E^ and RvvB^D57A^ substitutions on RvvAB regulation of *vpsL* (Fig 4C). In strains with RvvB^D57E^ and RvvB^D57A^ substitutions, *vpsL* transcription did not change compared to the wild type. In contrast, in the Δ*rvvA* strain, RvvB^D57E^ increased *vpsL* transcription 5-fold, while in RvvB^D57A^, *vpsL* transcription phenocopied that of the wild type. This finding suggests that the increased transcription of *vpsL* in Δ*rvvA* requires an active RvvB.

RvvB^D57E^ displayed increased *vpsL* transcription only in strains lacking RvvA, suggesting that an active Rvv TCS may upregulate *vpsL* but, under the conditions tested, RvvA keeps the system inactive. HKs mediate phosphorylation and subsequent dephosphorylation of their cognate RR [41,42]; we wondered if RvvA acts primarily as a phosphatase on RvvB under the conditions tested, preventing RvvB from modulating *vpsL* transcription. We thus aligned amino acid sequences of the HisKA Dhp domain of classic-type HKs in *V*. *cholerae* (Figs 4B and S5B) to determine the conserved histidine (H289) and glutamate (E290) of RvvA, predicted to be important for kinase activity, as well as the conserved threonine (T293), predicted to be important for phosphatase activity [41–43]. We independently substituted each of these residues with alanine (H289A, E290A, T293A) and replaced the wild-type gene in the chromosome with the mutated versions of *rvvA*. In contrast to our prediction, we observed that in strains with *rvvA* substitutions predicted to impair kinase or phosphatase activity, P*vpsL*-lux activity was similar to the wild type (Fig 4D).

### Mutations in the RvvA-HisKA and RvvB-REC domains impact *rvv* gene transcription

In the Rvv system, changes to biofilm gene transcription are evident only in the Δ*rvvA* genetic background. Therefore, we reasoned that the *rvv* operon’s transcription is autoregulated by RvvB, and increased transcription of the *rvv* operon is needed for the observed phenotypes. To test this hypothesis, we generated a P*rvv*-lux transcriptional fusion and analyzed promoter activity in *rvv* single and double deletion strains (Δ*rvvA*, Δ*rvvB*, Δ*rvvC*, Δ*rvvAB*, Δ*rvvAC*, Δ*rvvA*::*rvvA*) (Fig 4E). Compared to the wild type, Δ*rvvA*, Δ*rvvC*, and Δ*rvvAC* showed 256-fold, 12-fold, and 248-fold increases in P*rvv* transcription during exponential growth, respectively. Δ*rvvB* strains resulted in very low P*rvv* transcription, either singly or in combination with *rvvA*. These findings suggest that the *rvv* operon is autoregulated and that RvvB is required for this regulation. While the role of the predicted periplasmic protein RvvC in the modulation of RvvAB TCS signaling is unknown, the relatively small but reproducible *rvv* upregulation in Δ*rvvC* suggests that RvvC may be important for *rvv* activation.

We next measured P*rvv*-lux activity in strains harboring the mutated versions of RvvA and RvvB. In strains with *rvvA* substitutions predicted to impair kinase activity (RvvA^H289A^ and RvvA^E290A^), P*rvv*-lux activity was similar to wild-type. In contrast, in strains with *rvvA* substitutions predicted to impair phosphatase function, RvvA^T293A^ P*rvv*-lux activity was markedly increased, with a 130-fold increase in *rvv* transcription compared to the wild type (Fig 4E).

We additionally observed that in the Δ*rvvArvvB*^*D57E*^ strain, P*rvv*-lux promoter activity increased 225-fold compared to the wild type; this transcriptional activation was abolished in the Δ*rvvArvvB*^*D57A*^ strain. These findings suggest that, under the conditions tested, RvvA functions as a phosphatase keeping RvvB in a dephosphorylated state and resulting in basal *rvv* operon transcription. In contrast, in *rvvA* deletion and RvvA-phosphatase deficient strains, as well as in RvvB constitutively active strains, *rvv* operon transcription is increased, leading to activation of the Rvv TCS and associated phenotypes.

### RvvA contributes to intestinal colonization

**Fig 5:**
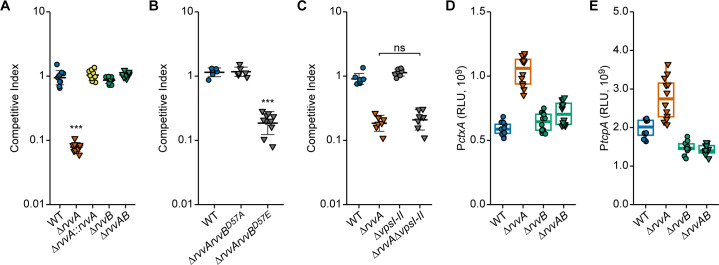
The role of the *rvv* system in host colonization. (A, B, C). The competitive index (CI) of indicated mutant strains was analyzed and compared to the CI of wild type during colonization of the infant mouse intestine. Each data point represents the CI in an individual mouse. (A, B) Statistical significance was determined using a One-Way ANOVA and post-hoc Tukey’s multiple comparisons test. Means from individual biological replicates (n ≥ 6) were compared to that of wild type, and differences with an adjusted *P* value of ≤ 0.01 were deemed significant. ***, *P* ≤ 0.0001. (C) Means from at least six biological replicates were compared by an unpaired t-test, and mean differences with a *P* value of ≤ 0.01 were considered significant. ns, not significant. (D, E) Promoter activity of the transcriptional fusions P*ctxA*-lux (D), and P*tcpA*-lux (E) were measured from cells grown to exponential phase in the indicated strains. Individual data points (circles–RvvA present; triangles–RvvA deleted) of Relative Luminescent Units (RLU) are displayed on top of a boxplot for each strain.

The involvement of *V*. *cholerae* TCSs in colonization and adaptability to host conditions is poorly understood. We evaluated the ability of RvvA to colonize the small intestine in an *in vivo* competition assay where the *in vivo* fitness of a mutant strain was compared to that of a wild-type strain using the infant mouse infection model. We found that Δ*rvvA* had a ~10-fold defect in *in vivo* colonization (Fig 5A). The wild-type copy of *rvvA* under the control of its native promoter in the Tn7 site (Fig 5A) complements the colonization defect, further confirming that RvvA regulates *in vivo* colonization. We next tested colonization phenotypes of Δ*rvvB and* Δ*rvvA*Δ*rvvB*; we observed that while Δ*rvvB* colonization is similar to that of the wildtype, Δ*rvvA*Δ*rvvB* strain phenocopies the Δ*rvvB* strain. We next performed an *in vivo* competition assay between wild type and strains harboring different phoshomimetic versions of RvvB in the *ΔrvvA* genetic background (Δ*rvvArvvB*^*D57A*^ and Δ*rvvArvvB*^*D57E*^) (Fig 5B). The competitive index of Δ*rvvArvvB*^*D57A*^ phenocopied wild type, while a Δ*rvvArvvB*^*D57E*^ strain showed a ~10-fold defect in *in vivo* colonization. These results suggest that the colonization defect observed for Δ*rvvA* depends on the presence and phosphorylation state of RvvB. The absence of RvvA leads to increased *vps* transcription and a defect in *in vivo* colonization. To determine if the presence of *vps* contributes to the colonization defect seen in Δ*rvvA*, we performed *in vivo* competition assays with Δ*rvvA*, Δ*vpsI-II*, and Δ*rvvA* Δ*vpsI-II* and wild-type strains (Fig 5C). In this experiment, Δ*rvvA* and Δ*rvvA ΔvpsI-II* showed a ~6-fold defect compared to the wild type. We did not observe any difference in colonization between Δ*rvvA* and Δ*rvvA* Δ*vpsI-II*. In contrast, Δ*rvvA* and Δ*rvvA* Δ*vpsI-II* showed a statistically significant defect in colonization compared to WT or Δ*vpsI-II*. These results suggest that the RvvA colonization defect is independent of increased *vps* transcription.

Based on the above results, we hypothesized that Δ*rvvA* might downregulate the transcription of some virulence factors, leading to the observed defect in colonization. Therefore, we examined the transcription of core virulence factors in *rvv* mutants grown under virulence-inducing conditions. We analyzed *ctx*, (cholera toxin), and *tcp*, *(*toxin co-regulated pilus) promoter activity in wild-type, Δ*rvvA*, Δ*rvvB*, and Δ*rvvAB* strains using P*ctx-*lux and P*tcp*-lux transcriptional fusions. (Fig 5D and 5E). Surprisingly, we found a small but consistent increase in *ctx* and *tcp* transcription in Δ*rvvA*. This observation suggests that changes in *ctx* and *tcp* transcription are not the source of Δ*rvvA*’s defect during *in vivo* colonization.

### Characterization of Δ*rvvA*’s regulon

To better understand how the Rvv TCS contributes to *V*. *cholerae* infection, we compared the transcriptional profiles of wild type and Δ*rvvA* grown under virulence-inducing conditions using RNA-seq. Differentially expressed transcripts were defined as those having an adjusted p-value ≤ 0.05, and a log_2_(fold-change) ≥ ±1. This provided 55 upregulated and 19 downregulated transcripts that were differentially expressed in Δ*rvvA* compared to wild type under these conditions.

We next examined the Rvv regulon in the context of GO terms (biological process), where we used a cut-off of at least two differentially expressed genes being present for a given GO pathway. We found increased message abundance in Δ*rvvA* compared to wild type for genes predicted to be involved in regulation of DNA-templated transcription, carbohydrate metabolic process, overall metabolic process, proton transmembrane transport, cation transport, and protein transport. Conversely, we found decreased message abundance in Δ*rvvA* compared to wild type for genes predicted to be involved in transmembrane transport, proteolysis, phosphorelay signal transduction system, aromatic amino acid metabolic process, and signal transduction.

**Fig 6:**
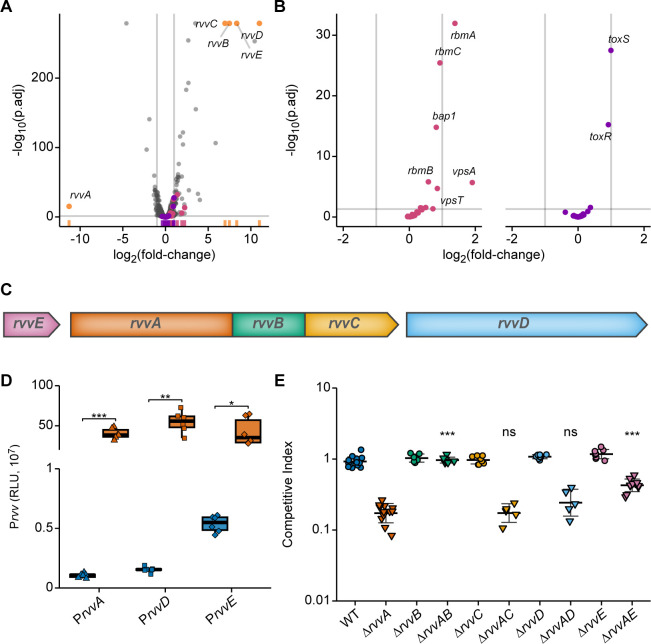
Rvv regulon and its contribution to virulence. (A) RNA-seq analysis of Δ*rvvA* and wild-type strains grown under virulence-inducing conditions (AKI). Volcano plots display differential transcript abundance in Δ*rvvA* compared to wild type (n = 3). The negative log of the adjusted p-value (base 10) is plotted on the y axis, and the log of the fold-change (FC) (base 2) is plotted on the x axis. Each point represents a transcript. Gray lines indicate cutoffs for differential expression–a log2 FC with an absolute value greater than 1 (vertical lines), and an adjusted p-value less than 0.05 (horizontal line). A log_2_(fold change) > 0 indicates increased expression of a transcript in Δ*rvvA* compared to wild type. Differential coloring is used for transcripts of genes involved in biofilm formation (pink), pathogenesis (purple), or comprising the *rvv* loci (orange-yellow). A marginal distribution runs along the x-axis depicts the density of transcripts from a given pathway within the plot. (B) Volcano plots with subsets of the data shown in (A), displaying only transcripts of genes involved in biofilm formation (pink) and pathogenesis (purple). (C)—Genomic region representing *rvvABCDE* loci (VCA0258-VCA0254). (D)—Promoter activity of P*rvv*-lux transcriptional fusions from upstream regulatory regions of *rvvA* (triangles), *rvvD* (squares), and *rvvE* (diamonds) was measured from exponentially grown cells in wild type (blue) and Δ*rvvA* (orange). Individual data points of Relative Luminescence Units (RLU) are overlaid on top of crossbars displaying the mean and standard deviation. For each transcriptional fusion, means from at least three biological replicates were compared by an unpaired t-test. *, *P* ≤ 0.01; **, *P* ≤ 0.001; ***, *P* ≤ 0.0001. (E)–The competitive index (CI) of indicated strains to colonize the infant mouse intestine were analyzed using a competition assay with an isogenic wild-type strain. Each data point represents the CI in an individual mouse. Statistical significance was determined using a One-Way ANOVA and post-hoc Tukey’s multiple comparisons test. Means from individual biological replicates (n ≥ 6) were compared to that of Δ*rvvA*, and differences with an adjusted *P* value of ≤ 0.01 were deemed significant. ***, *P* ≤ 0.0001; ns, not significant.

The transcripts that had the highest increase in abundance in Δ*rvvA* compared to wild type were those encoding genes located in the genomic region surrounding *rvvA* (VCA0257) (Fig 6A and 6C). The transcript abundance for VCA0258 and VCA0254, the upstream and downstream genes adjacent to the *rvvABC* operon, encoding genes of unknown function, were up-regulated 324-fold and 2044-fold, respectively.

We now term these genes *rvvE* (VCA0258) and *rvvD* (VCA0254). We analyzed the promoter activity of P*rvvA-lux*, *PrvvD-lux*, and P*rvvE-lux* transcriptional fusions from cells grown in nutrient broth (Fig 6D). Consistent with our RNA-seq results, these transcriptional fusions also displayed increased promoter activity in Δ*rvvA* compared to wild type. The transcripts encoding the Rvv TCS system, *rvvB* (VCA0256), and *rvvC* (VCA0255) were also increased 178-fold, and 125-fold in Δ*rvvA*, respectively.

In addition to *rvvABCDE*, many transcripts encoding biofilm genes were higher in abundance in Δ*rvvA* compared to wild type. *vpsU* (VC0916), *vpsA* (VC0917), *rbmA* (VC0928), *rbmC* (VC0930), *bap1* (VC1888), *vpsT* (VCA0952), *rbmB* (VC0929), *vpsL* (VC0934), *vpsD* (VC0920), *vpsF* (VC0922), and *vpsK* (VC0927) were significantly increased, with an average of 1.83-fold in Δ*rvvA*. Among these, *vpsU*, *vpsA*, *and rbmA* were 4.56, 3.34, 2.58-fold, respectively.

Of the *V*. *cholerae* virulence regulon, we found that only transcripts encoding *toxR* and *toxS* were differentially increased in Δ*rvvA*, further supporting our observation that Δ*rvvA*’s *in vivo* fitness defect is not due to decreased transcription of core virulence factors.

Since the transcript abundance of genes surrounding the *rvv* loci was highest in Δ*rvvA*, we next analyzed the contribution of each *rvv* gene to the *in vivo* fitness defect. We deleted *rvvB* through *rvvE* in WT and Δ*rvvA* genetic backgrounds, and then performed *in vivo* competition assays (Fig 6E). In the WT background, deletion of *rvvB*, *rvvC*, *rvvD*, and *rvvE* did not impact *in vivo* fitness. In the Δ*rvvA* background, only deletion of *rvvE* abrogated the *rvvA* fitness defect by 4-fold. Taken together, these results suggest that increased transcription of *rvv* contributes to the *in vivo* fitness defect of Δ*rvvA* by a yet to be determined mechanism.

### Comparative genomic analysis of the *rvv* loci

**Fig 7:**
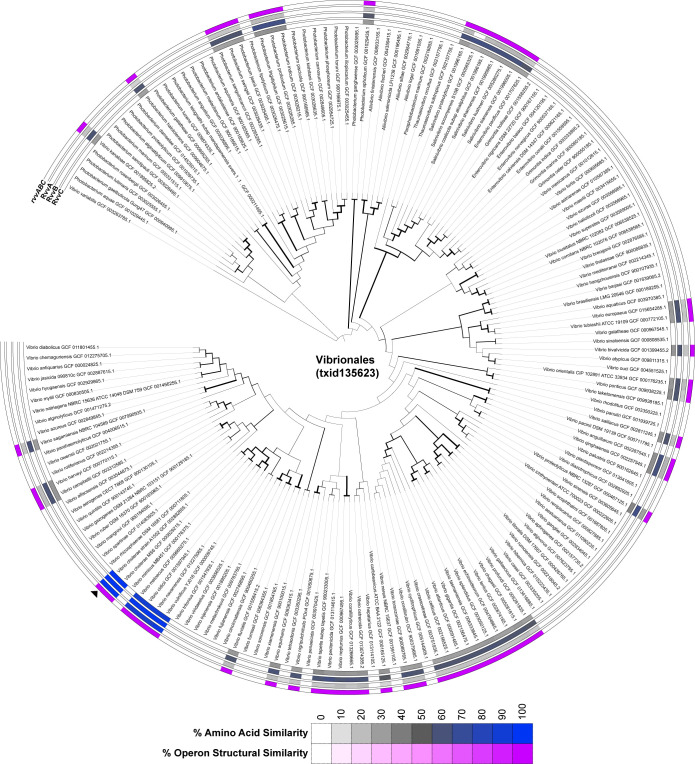
Conservation of *rvvABC* in Vibrionales. The conservation of *rvvABC* genes across the Vibrionales order was assessed and structural similarity scores and individual gene percent identities were annotated on a RecA reference phylogeny using the iTOL web service [62]. Amino acid % similarity of Rvv homologs is shown for each protein encoded in the *rvv* loci. % similarity is shown as a gradient from grey to blue, with blue representing the highest similarity. On the outermost ring, structural similarity of the *rvv* genomic region is visualized as a gradient from white to purple, with purple representing the highest structural similarity.

To further contextualize the role and possible function of the *rvv* locus, we performed a comprehensive reciprocal BLAST analysis of the structural and sequence conservation of *rvvABC* loci across Vibrionales representative genomes, superimposing the findings on a RecA phylogeny (Fig 7). Our results show that the structure of the *rvvABC* locus is conserved in all the Vibrionales species in which homologs to any of its constituent genes are detected, suggesting that it operates as a functional operon across this clade. The *rvvABC* locus presents a markedly uneven distribution across the Vibrionales, and it is detected in only 55 (32%) of the 171 representative genome assemblies analyzed. The *rvvABC* operon is consistently detected in several large clades within the *Photobacterium* and *Salinivibrio* genera, as well as in different groups of marine *Vibrio* associated with mollusks echinoderms and other marine invertebrates (e.g. *Vibrio neptunius*, *Vibrio echinoideorum*, *Vibrio atlanticus*). Outside these clades, the *rvvABC* locus presents a scattered distribution that is strongly suggestive of lateral gene transfer. The genomic context of the *rvvABC* locus is highly variable (S4C Fig), and shows evidence in several Vibrionales species (e.g. *Vibrio tapetis*) of association with chromosomally-encoded mobile genetic elements. The *rvvABC* locus is consistently detected in several close relatives of *V*. *cholerae* (*Vibrio metoecus*, *Vibrio navarrensis*, *Vibrio vulnificus*) suggesting that it was acquired by a recent ancestor of this group of human pathogens. The distribution of the *rvvABC* locus is in stark contrast with the one observed for the *vxrABCDE* operon, whose HK (*vxrA*) is the closest homolog of *rvvA* in the Vibrionales. The *vxrABCDE* operon structure and genomic context are highly conserved across the *Vibrio* genus (S2 and S4C Figs), suggesting an ancestral role for this operon in *Vibrio* biology [22]. This suggests that the *rvvABC* locus implements a specialized signal transduction system that has been acquired or retained by different Vibrionales species.

## Discussion

TCSs are commonly used by bacteria to sense and respond to their environment. Prototypical TCSs have a membrane-embedded histidine kinase that recognizes an external input and phosphorylates a response regulator that regulates gene transcription. In both the aquatic environment and the human host, *V*. *cholerae* encounters multiple changing inputs from the extracellular environment [44]. TCSs allow the bacterium to integrate these signals and, in response, adjust various cellular processes. Biofilm formation is one critical cellular process contributing to the *V*. *cholerae* infection cycle; however, the repertoire of TCSs governing biofilm formation has not been fully evaluated. In this study, we systematically analyzed the impact of HKs on biofilm gene transcription in *V*. *cholerae*. We noticed that most HKs lacked either statistically significant deficiencies in biofilm gene transcription or had modest defects. These TCSs may have a role in biofilm formation; however, they are either not expressed, or their cognate signal is absent under the experimental conditions utilized in this study.

In our earlier work, we analyzed *vpsL* transcription in RR deletion mutants in *V*. *cholerae* under the same conditions used in our HK deletion screen [33]. We found that 7 RR deletion mutants displayed significant changes in *vpsL* transcription. In Δ*vpsR*, Δ*vpsT*, *ΔluxO*, and Δ*vxrB* strains, *vpsL* transcription decreased compared to wild type. VpsR and VpsT are orphan response regulators. While VpsT activity does not depend on phosphorylation, VpsR activity is dependent upon the conserved aspartate residue predicted as a phosphorylation target [30,45,46]. Cognate histidine kinase(s) of VpsR remain unknown. LuxO is phosphorylated by a phospho-relay involving LuxU, an Hpt, through histidine kinases LuxQ (VCA0736), VpsS (VC1445), CqsR (VC1831), and CqsS (VCA0522) [8,9,24]. In this study, we found that *vpsL* transcription was decreased in Δ*luxQ* and Δ*cqsR*, increased in Δ*cqsS*, and unchanged in Δ*vpsS*. In Δ*vxrA*, a strain lacking the cognate histidine kinase of VxrB, *vpsL* transcription was decreased. In Δ*carR* and *ΔntrC* strains, *vpsL* transcription increased compared to wild type [21,36]. Consistent with this finding, we also observed that Δ*carS* and Δ*ntrB* displayed increased *vpsL* transcription. In Δ*varS*, *vpsL* transcription was markedly increased, which is consistent with the known role of the VarSA system in regulating *vps* transcription and biofilm formation [6]. Additionally, we observed that deletion of four previously uncharacterized HKs VC1156-7, VC0694, VC1605, and VCA0257 (RvvA) resulted in increased *vpsL* transcription.

We mainly focused on RvvABC, a novel regulator of *vps* and biofilm formation and pathogenicity. We showed that the sensor histidine kinase RvvA represses biofilm gene transcription and promotes mouse colonization. Somewhat surprisingly, given the large increase in *vpsL*, biofilms formed in Δ*rvvA* had modestly altered structural properties compared to the wild type. This finding correlated well with the observation that there were no changes in c-di-GMP levels or motility in Δ*rvvA*. This outcome suggests that RvvA is not a global c-di-GMP regulator, but instead has a more focused effect on *vpsL* transcription. RvvA repressed transcription of VpsR and VpsT, and epistasis analysis found that the increase in *vpsL* transcription seen in Δ*rvvA* was dependent on VpsR and VpsT, but not HapR. The increases in biofilm gene transcription in Δ*rvvA* were not the source of Δ*rvvA*’s survival defect during *in vivo* colonization, as Δ*rvvA* Δ*vpsI-II* displayed the same competitive fitness as Δ*rvvA*. *ctxA* and *tcpA* transcription was increased in Δ*rvvA* compared to wild type, suggesting a change in virulence gene transcription was also not the cause of the *in vivo* fitness defect. Comparison of transcriptional profiles of wild type and Δ*rvvA* under virulence-inducing conditions supported the idea that differential transcription of core virulence factors is not driving the *in vivo* fitness defect of Δ*rvvA*. The *rvv* regulon comprises many genes of unknown function. Further investigation is needed to determine the mechanism by which the Rvv regulon modulates different functions in *V*. *cholerae*.

**Fig 8:**
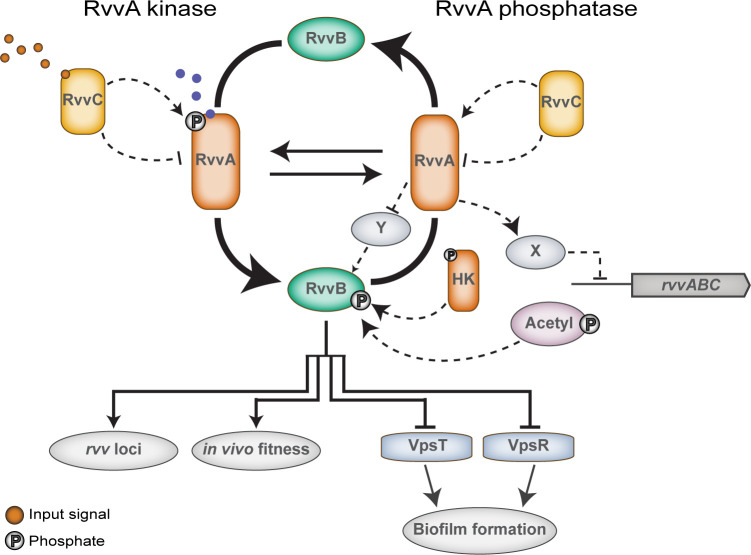
Model of Rvv TCS signal transduction. In the wild-type background, deletion of the gene encoding the sensor histidine kinase RvvA increases transcription of the *rvv* loci. Increased *rvv* transcription is dependent on both the presence and phosphorylation-state of the response regulator RvvB. However, RvvB^D57E^ is not sufficient to increase target gene transcription unless RvvA is lacking. This suggests that *rvv* transcription might be repressed by an unknown repressor that requires the presence of RvvA, or that RvvA is inhibiting a key protein/signal needed for the function/activation of RvvB and thus presenting RvvB associated phenotypes. A strain harboring RvvA^T293A^, a mutated form of RvvA that abolishes phosphatase function, phenocopies Δ*rvvA*, supporting a model where RvvA acts as a phosphatase on RvvB. Under the conditions tested, RvvB phosphorylation may be mediated by acetyl-phosphate or crosstalk with another HK. RvvA’s kinase function may be activated in response to a specific signal, leading to RvvB phosphorylation. The impact of RvvC on RvvAB phosphotransfer and the identity of signals sensed by RvvA or RvvC, have yet to be determined. Increased transcription and phosphorylation of RvvB activates the Rvv TCS, increasing transcription of *rvv* loci and biofilm formation genes, and reducing *in vivo* fitness.

The phenotypes in Δ*rvvA* depend on the presence of RvvB, the predicted cognate response regulator of RvvA. In canonical two-component systems, the sensor histidine kinase activates the response regulator upon detecting an input signal, and the response regulator elicits cellular output. HKs are often bifunctional and can dephosphorylate their cognate RRs, and signals can stimulate either the kinase or phosphatase activity of the HK [41,42]. We reason that, under the experimental conditions utilized in this study, RvvA’s HK activating signal is not present, and therefore RvvA functions as a phosphatase. RvvA’s input signal may modulate RvvA’s kinase/phosphatase activity, shifting RvvA from predominantly phosphatase to predominantly kinase when a signal is present and coordinate biofilm formation through activation of RvvB (Fig 8).

Two-component system genes must be transcribed at a basal level to produce enough sensor HK and regulators to detect and respond to specific signals. Numerous two-component systems contain both a constitutive and an autoregulated promoter, which is necessary to produce high levels of regulators to transduce and respond to signals, thereby allowing environmental adaptation [47]. We determined that the *rvv* TCS is autoregulated. This feedback regulation would be energetically beneficial, particularly for facultative pathogens such as *V*. *cholerae*, because this strategy would prevent unnecessary activation of cellular processes. In support of this hypothesis, the mutation of a conserved residue implicated in phosphatase activity (RvvA^T293A^), elevated *rvv* transcription similarly to the increase in transcription observed in Δ*rvvA*.

RvvB may be phosphorylated and active in Δ*rvvA*. In this case, a small-molecule donor, such as acetyl-phosphate, may phosphorylate RvvB in RvvA’s absence, as seen for other RRs [48,49] (Fig 8). Alternatively, cross-talk may occur between RvvB and the HK of a different TCS [50] (Fig 8). Phosphorylation of RvvB by another HK may not be detected if RvvA simultaneously dephosphorylates it. Only in the absence of RvvA could a phosphorylated version of RvvB persist in the cell and elicit a response. Further study is needed to determine the mechanisms that control Rvv TCS activation.

The RvvABC structural and sequence conservation analysis revealed that the RvvABC system shares similar domains with another *V*. *cholerae* TCS, VxrABC [22]. The RvvA domain organization is similar to that of the VxrABC HK, VxrA, with both possessing a periplasmic sensing region (SD) and a cytoplasmic DUF3404 domain (Pfam PF11884). The VxrA periplasmic domain has been crystallized, and revealed that it has a unique structural fold that forms an uncommon hairpin-swapped dimer. VxrA lacks a cytoplasmic linker region between the second transmembrane helix and the dimerization and histidine phosphotransfer (DHp) domain [51]. Structural studies revealed that the conformational change brought about by the relative rotation of the two monomers in a VxrA-SD dimer might alter the connection of transmembrane helices and, consequently, the pairing of cytoplasmic DHp domains, thereby transferring the ligand-binding signal from the periplasmic SD to the cytoplasmic kinase domain [51]. It is possible that VxrA and RvvA share similar activation mechanisms as many of the residues important for function in VxrA’s sensing domain are conserved in RvvA. RRs of both the Rvv and Vxr systems, RvvB and VxrB, share an N-terminal REC domain and a C-terminal winged helix-turn-helix DNA-binding domain with RR of the OmpR family. Finally, RvvC and VxrC share ~20% sequence identity, are both predicted to be periplasmic, and contain a DUF2861 domain whose function remains unknown.

RvvA/VxrA and Rvvc/VxrC represent the first examples in which the phenotypic consequences of mutating proteins with DUF3404 or DUF2861 domains are explored. In previously published work, VxrA and VxrB positively control *vpsL* transcription and biofilm development. However, VxrC acts as a biofilm repressor, and this phenotype depends on the presence of VxrB. VxrC interaction with the periplasmic domain of VxrA appears to inhibit VxrA and VxRB activation, though the molecular details are unknown. In the Rvv system, under the conditions used in this study, RvvA and RvvC, albeit slightly, negatively control *vpsL* transcription; this phenotype depends on the presence of RvvB. Thus, in both systems, the predicted periplasmic component plays a role in signal transduction. The cognate signal(s) governing the activity of RvvA and VxrA systems is unknown and, similarly, the molecular details of the signal transduction mechanism and the role the periplasmic proteins RvvC and VxrC play in this process are yet to be determined.

Since the Rvv and Vxr systems share overall operon structure and domain organization, we undertook comparative genomics analysis to determine the phylogenetic distribution of these systems. Comparative genomics analysis of the *rvvABC* locus revealed that this locus is present in multiple Vibrionales species and maintains a conserved operon structure but presents an overall scattered distribution across this phylogenetic order. Additionally, when the *rvvABC* locus is found, there is no evidence of genomic context conservation. The phylogenetic heterogeneity of the *rvvABC* operon and its lack of genomic context conservation contrast with those observed in the *vxrABCDE* operon. The *vxrA*-encoded histidine kinase is highly conserved and displays substantial genomic context conservation across the *Vibrio* genus, indicative of an ancestral role in the *Vibrio* genome. The phylogenetic and genomic context evidence, therefore, makes a strong case for multiple instances of lateral gene transfer of the *rvvABC* locus within the Vibrionales order. This suggests that the *rvvABC* operon codes for a specialized signal transduction pathway that has been acquired or selectively kept by a diverse group of Vibrionales species with very different habitats, including the group of human pathogens exemplified by *V*. *cholerae*. Our findings indicate that the Rvv TCS is an important regulator of biofilm gene transcription and *in vivo* colonization in *V*. *cholerae*. Further work characterizing the purpose of this TCS in *V*. *cholerae*’s life cycle may shed light on novel regulatory mechanisms connecting biofilm formation and virulence.

## Materials and methods

### Bacterial strains and growth conditions

The strains and plasmids used in this study are listed in S1 Table. *E*. *coli* CC118-λ*pir* and DH5α-λ*pir* strains were used for DNA manipulation, and *E*. *coli* S17-1λpir strains were used for conjugation with *V*. *cholerae*. *V*. *cholerae* and *E*. *coli* strains were grown aerobically at 30°C and 37°C, respectively, unless stated otherwise. Luria-Bertani Miller (LB) broth contained 1% tryptone, 0.5% yeast extract, 1% NaCl [pH 7.5]. LB agar medium included granulated agar (BD Difco, Franklin Lakes, NJ) at 1.5% (wt/vol); except for motility agar which was 0.3% (wt/vol). When AKI medium was used, media components and growth conditions were followed as previously described [52]. Antibiotics and inducers were used, when necessary, at the following concentrations: ampicillin (Ap), 100 μg/mL; rifampicin (Rif), 100 μg/mL; gentamicin (Gm), 15 μg/mL; chloramphenicol (Cm), 20 μg/mL for *E*. *coli* and 5 μg/mL or 2.5 μg/mL for *V*. *cholerae*. Unless specified otherwise, overnight cultures for experiments were prepared as follows: strains were struck from frozen glycerol stock onto an LB-agar plate and grown at 30°C overnight, 5 colonies were then inoculated into 5 mL LB media and grown overnight at 30°C with aeration (200 rpm).

### Strain and plasmid construction

Plasmids were constructed using standard molecular cloning techniques or the Gibson Assembly recombinant DNA technique (New England BioLabs, Ipswich, MA). In-frame gene deletions were generated through allelic exchange of native open reading frame (ORF) with the truncated ORF, as previously described [53].

### Luminescence assay

*V*. *cholerae* strains harboring transcriptional reporters were grown overnight with aeration in LB broth supplemented with chloramphenicol 5 μg/mL. Cultures were diluted 1:200 into fresh LB containing chloramphenicol 2.5 μg/mL. The freshly inoculated cultures were grown aerobically at 30°C to exponential phase (OD_600_ of 0.3 to 0.4) and then luminescence was measured using a PerkinElmer Victor3 multilabel counter (PerkinElmer, Waltham, MA). For the HK single deletion P*vpsL*-lux screen, cells were diluted 1:200 from 5ml ON cultures into 200μl LB and grown in 96-well plates statically, but all conditions kept identical otherwise. For non-P*vps* reporters, to identify how gene transcription changes over time, cells were grown in a 96-well plate in a heated plate reader for at least 15 hours, with OD_600_ and luminescence measurements taken every 30 minutes. A representative measurement with exponential phase OD_600_ was used for visualization and comparison. Luminescence activity is reported as relative luminescence units (RLU; counts min−1 ml−1/OD_600_ unit). Assays were repeated for a minimum of three independent biological replicates, with three technical replicates measured for all assays.

### c-di-GMP dual fluorescent reporter assay

Intracellular c-di-GMP levels were evaluated using a fluorescent reporter as previously described [54,55]. *V*. *cholerae* strains harboring a c-di-GMP dual-fluorescent biosensor (pFY_4535) were grown overnight with aeration in LB broth supplemented with Gentamicin. Cultures were diluted 1:200 into fresh LB. The freshly inoculated cultures were grown aerobically at 30°C to exponential phase (OD_600_ of 0.3 to 0.4) and then fluorescence was measured in Corning 96-well, clear-bottom, black, polystyrene microplates using a PerkinElmer Victor3 multilabel counter (PerkinElmer, Waltham, MA). 460/480 nm and 550/580 nm excitation/emission filters were used to measure fluorescence intensity for Amcyan (normalizer) and TurboRFP, respectively. c-di-GMP levels are reported as relative fluorescence intensity (RFI). RFI was calculated from the ratio of fluorescence intensity of TurboRFP to Amcyan. Assays were repeated for a minimum of three independent biological replicates, with three technical replicates measured for all assays.

### Swimming motility assay

Motility assays were performed in LB media containing 0.3% (wt/vol) agar. 100 ml of LB agar media was dried at room temperature in 150mm diameter petri dishes. After 22h drying, single colonies of *V*. *cholerae* strains were stabbed into the motility plate. Strains were positioned with equidistant spacing from center and edges of the petri dish, as well as equal spacing from other strains. After 16h, the diameter of the swimming area surrounding the inoculum position was measured and compared. Assays were repeated for a minimum of four independent biological replicates; each motility plate was treated as an independent biological replicate.

### Static biofilms and CLSM

Diluted overnight cultures of *gfp-*tagged *V*. *cholerae* strains were used for inoculation of static biofilms. 1 milliliter of 1:200 diluted cells (OD_600_ of 0.02) was introduced to a static biofilm chamber (Ibidi #80281*)*. Post-inoculation, static biofilm chambers were incubated at 30°C in LB with no aeration or movement. After 6-hours, static biofilms were gently washed twice in PBS. Biofilms were kept in PBS throughout imaging, and images were taken promptly after washing. Images of biofilms were captured with an LSM 880 (Zeiss, Jena, Germany), using an excitation wavelength of 488 nm and an emission wavelength of 543 nm. Three-dimensional (3D) images of the biofilms were processed using Imaris software (Bitplane, Zurich, Switzerland). All image processing parameters in Imaris (opacity, min, max, etc.) were kept identical between images for accurate comparison. BiofilmQ was used for quantitative analysis of biofilm parameters [40].

### Growth curves

*V*. *cholerae* strains were grown aerobically overnight in LB. Strains were diluted 1:200 into one well of a 96-well plate in 200uL. 96-well plates were grown in a plate reader at 30°C. OD_600_ measurements were taken hourly after brief shaking to stir the cells for the measurement. At least 3 biological replicates (independent overnight cultures) and 3 technical replicates (same overnight culture in different wells) were used for comparison of growth between different strains.

### Intestinal colonization assay

*In vivo* competition assays for intestinal colonization determination were performed as described previously [56]. In brief, cultures of relevant strains were grown to stationary phase at 30°C with aeration in LB broth. Each *lacZ*^*+*^ *V*. *cholerae* mutant strain was mixed with the wild-type reference strain (*lacZ*^*-*^, otherwise wild-type) in a 1:1 ratio in 1x PBS. The inoculum was plated on LB agar plates containing 5-bromo-4-chloro-3-indoyl-β- D-galactopyranoside (X-gal) to differentiate colonies of the reference strain from mutants and determine input ratios. 10^6^–10^7^ cfu of the input inoculum mixture was oral gavage administered to groups of 5–7 anesthetized 5-day old CD-1 mice (Charles River Laboratories, Hollister, CA). At 22h post-inoculation, small intestines were removed, weighed, homogenized, and plated on appropriate selective and differential media to obtain output ratios. *In vivo* competitive indices were then calculated by dividing the small intestine output ratio by inoculum input ratio of mutant to wild-type strains.

### RNA isolation

*V*. *cholerae* strains were grown overnight in LB broth at 37°C, and then diluted 1:100 into 10 ml of fresh AKI medium in borosilicate glass test tubes (diameter: 15mm, height: 150mm), and grown statically at 37°C. After 4 hours, cultures were transferred to 125ml flasks and grown on an orbital shaker with increased aeration (250 rpm). After 1 hour of increased aeration, 2 ml of each culture was centrifuged (16 K, 30 seconds), and the resulting pellet was immediately resuspended in 1 ml TRIzol (Invitrogen), flash-frozen, and subsequently stored at -80°C until RNA isolation. Total RNA isolation was performed according to the TRIzol manufacturer’s instructions. DNAse treatment, rRNA depletion (RiboZero Plus, Illumina), and library prep (Stranded RNA library preparation, Illumina) were performed according to manufacturer’s instructions. Sample QC was performed via Bioanalyzer. Illumina sequencing was performed for paired-end 150 bp reads.

### RNA-sequencing data analysis

Quality checks were performed on read data with FASTQC, version 0.11.9 [57]. Trimming was not performed as it was deemed unneeded from FASTQC analysis output. Transcript abundance was quantified with Salmon, version 1.5.2, using a recently inferred *V*. *cholerae* transcriptome derived from the N16961 reference genome as an index [58,59]. The resulting salmon quantification files were then imported into R via tximport [60]. Counts were normalized and differential expression analysis was performed using DESeq2, version 1.32.0 [61].

### Operon conservation analysis

The conservation of *rvvABC* genes was assessed using the *operon_conserve_detect* Python script (https://github.com/ErillLab/oprn_consv_calc). The script automates independent tBLASTN searches with each of the provided proteins for a given genomic locus against the NCBI RefSeq representative genomes database (*ref_prok_rep_genomes*) restricted to a taxon of interest (*Vibrio* (txid:662) and Vibrionales (txid: 135623)). tBLASTN hits were restricted to a maximum e-value of 10^−10^ and a minimum coverage of 30%. Inferred homology was strengthened by filtering hits based on whether a BLASTP of the hit against the reference genome returned the reference locus protein (i.e., reverse BLAST). The nucleotide records for the resulting hits were grouped by genome using their assembly accession number. For each nucleotide record, hits to the reference locus are grouped into operons based on the following criteria: (1) all genes in an operon must be on the same strand, (2) no more than three features can span the distance between two hits to the reference operon, and (3) the intergenic distance between operon genes must be less than 150 base pairs. Locus structure is evaluated by computing the split distance of the locus, using its reference instance for normalization (see S1 Data). Given a reference locus organization with *N* genes, the set of possible gene-pairs within the locus, *P*_*ref*_, has a size of *N*-1. For any given genome, the total possible number of each operon gene-pairs (*M*) is computed based on the number of hits to each reference gene in the specific gene-pair. The number of observed pairs *T* (pairs within predicted operons following the criteria above) is then computed, and for each pair the fraction *T/M* is considered the relative number of occurrences for that pair (*R*). The sum of relative occurrences, *R*, divided by the number of reference gene-pairs, *P*_*ref*_, is the structural similarity score, *Y*, ranging from 1 (total conservation) to zero (no pairs conserved). Structural similarity scores and individual gene percent identities were annotated on a reference phylogeny using the iTOL web service [62]. The reference phylogeny was generated using *Escherichia coli* RecA (WP_000963143.1) homologous sequences detected by BLASTP in all species of interest using the *phylo_seq_gen* Python script (https://github.com/ErillLab/phylo_seq_gen). The RecA sequences were aligned with CLUSTALW, and the resulting alignment was filtered with Gblocks [63]. This alignment was used to construct a Maximum Likelihood tree and infer bootstrap support (1,000 pseudo-replicates) using the MEGAX software suite [64].

## Supporting information

S1 Fig Conservation of *rvvABC* in Vibrios. The conservation of *rvvABC* genes across the Vibrio genus was assessed and structural similarity scores and individual gene percent identities were annotated on a RecA reference phylogeny using the iTOL web service [62]. Amino acid % similarity of Rvv homologs is shown for each protein encoded in the *rvv* loci. % similarity is shown as a gradient from grey to blue, with blue representing the highest similarity. On the outermost ring, structural similarity of the *rvv* genomic region is visualized as a gradient from white to purple, with purple representing the highest structural similarity.
(PDF)

S2 Fig Conservation of *vxrABCDE* in Vibrios. The conservation of *vxrABCDE* genes across the *Vibrio* genus was assessed and structural similarity scores and individual gene percent identities were annotated on a RecA reference phylogeny using the iTOL web service [62]. Amino acid % similarity of Rvv homologs is shown for each protein encoded in the *rvv* loci. % similarity is shown as a gradient from grey to blue, with blue representing the highest similarity. On the outermost ring, structural similarity of the *rvv* genomic region is visualized as a gradient from white to purple, with purple representing the highest structural similarity.
(PDF)

S3 Fig Conservation of *vxrABCDE* in Vibrionales. The conservation of *vxrABCDE* genes across the Vibrionales order was assessed and structural similarity scores and individual gene percent identities were annotated on a RecA reference phylogeny using the iTOL web service [62]. Amino acid % similarity of Rvv homologs is shown for each protein encoded in the *rvv* loci. % similarity is shown as a gradient from grey to blue, with blue representing the highest similarity. On the outermost ring, structural similarity of the *rvv* genomic region is visualized as a gradient from white to purple, with purple representing the highest structural similarity.
(PDF)

S4 Fig Predicted domains and genomic organization of the Rvv and Vxr TCSs. (A-B) Genomic organization (top) and predicted domains per protein (bottom) of the Rvv (A) and Vxr (B) TCSs in *V*. *cholerae*. (C) GeCoViz rendition of the surrounding genomic region of *rvv* and *vxr* in representative Vibrio species [65].
(PDF)

S5 Fig Phosphotransfer residue conservation in *V*. *cholerae* TCS. ClustalO Amino acid sequence alignment (right) and phylogenetic tree (left) of the (A) REC domain of OmpR-like response regulators in *V*. *cholerae* and (B) the HisKA DHp domain of Classic-type histidine kinases (HKs) in *V*. *cholerae*. For the HKs, the regions of the domain with conservation are shown, corresponding to regions 278–317 and 318–346 of RvvA’s amino acid sequence. Phylogenetic trees were generated from sequence alignment via neighbor-joining using BLOSUM62.
(PDF)

S6 Fig Alignment of RvvA and homologous representative RefSeq proteins. BlastP sequence alignment of RvvA and homologous proteins from selected high % identity species. Residues with 100% conservation across all selected species are shown in blue.
(PDF)

S7 Fig Alignment of RvvB and homologous representative RefSeq proteins. BlastP sequence alignment of RvvB and homologous proteins from selected high % identity species. Residues with 100% conservation across all selected species are shown in blue.
(PDF)

S8 Fig Alignment of RvvC and homologous representative RefSeq proteins. BlastP sequence alignment of RvvC and homologous proteins from selected high % identity species. Residues with 100% conservation across all selected species are shown in blue.
(PDF)

S1 Table Table of strains and plasmids used in this study. (PDF)

S1 Data Comparative genomics operon structure similarity calculation. (PDF)

S2 Data Comparative genomic analysis result tables. (ZIP)
