# Supplementary figures and images for: The Rvv two-component regulatory system regulates biofilm formation and colonization in Vibrio cholerae
Source: PLoS Pathog. 2023 May 22;19(5):e1011415. doi: 10.1371/journal.ppat.1011415 (PMC10237652; doi:10.1371/journal.ppat.1011415)

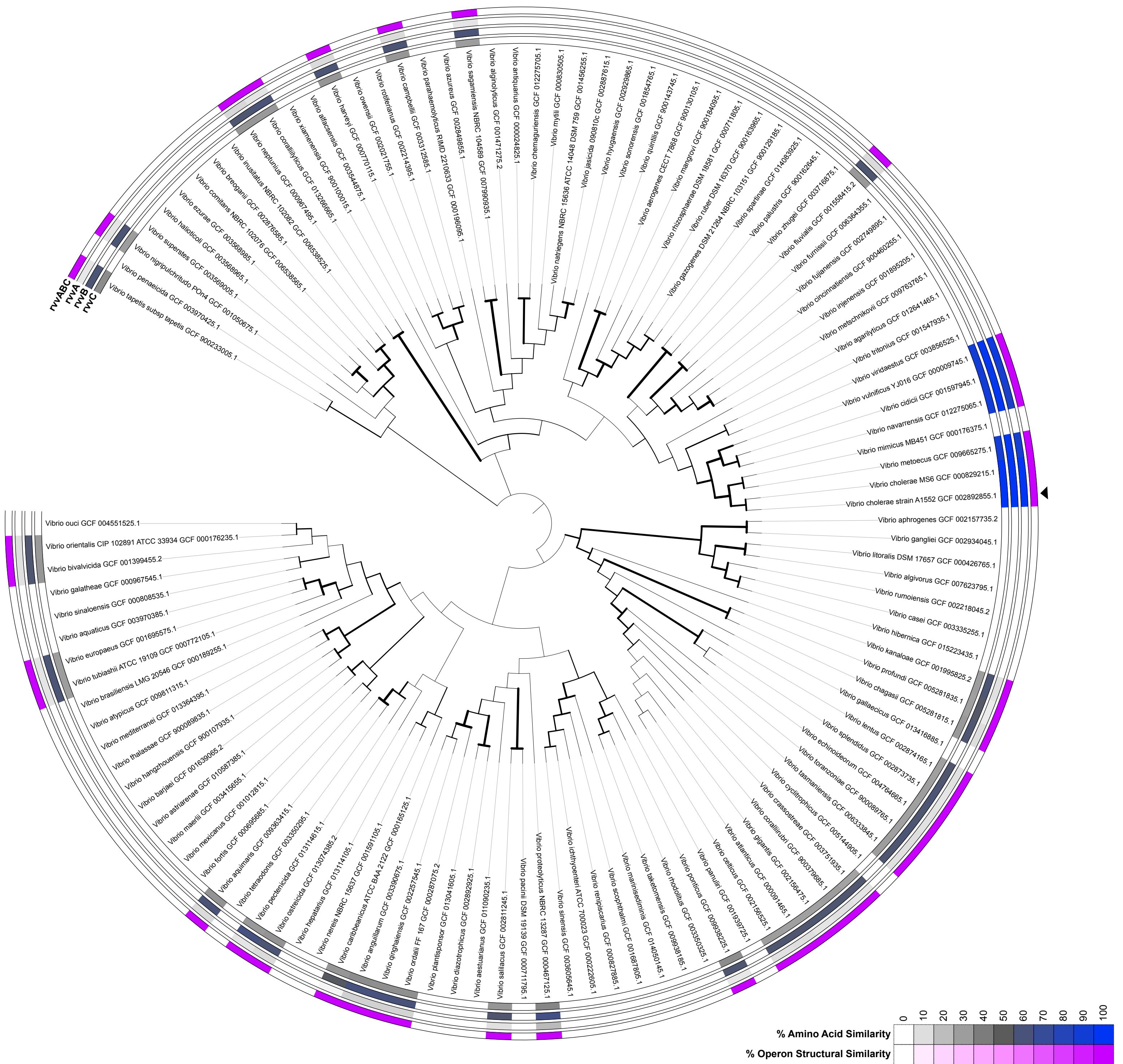

Supplement: S1 Fig — The conservation of rvvABC genes across the Vibrio genus was assessed and structural similarity scores and individual gene percent identities were annotated on a RecA reference phylogeny using the iTOL web service [62]. Amino acid % similarity of Rvv homologs is shown for each protein encoded in the rvv loci. % similarity is shown as a gradient from grey to blue, with blue representing the highest similarity. On the outermost ring, structural similarity of the rvv genomic region is visualized as a gradient from white to purple, with purple representing the highest structural similarity. (PDF) [file ppat.1011415.s001.pdf]

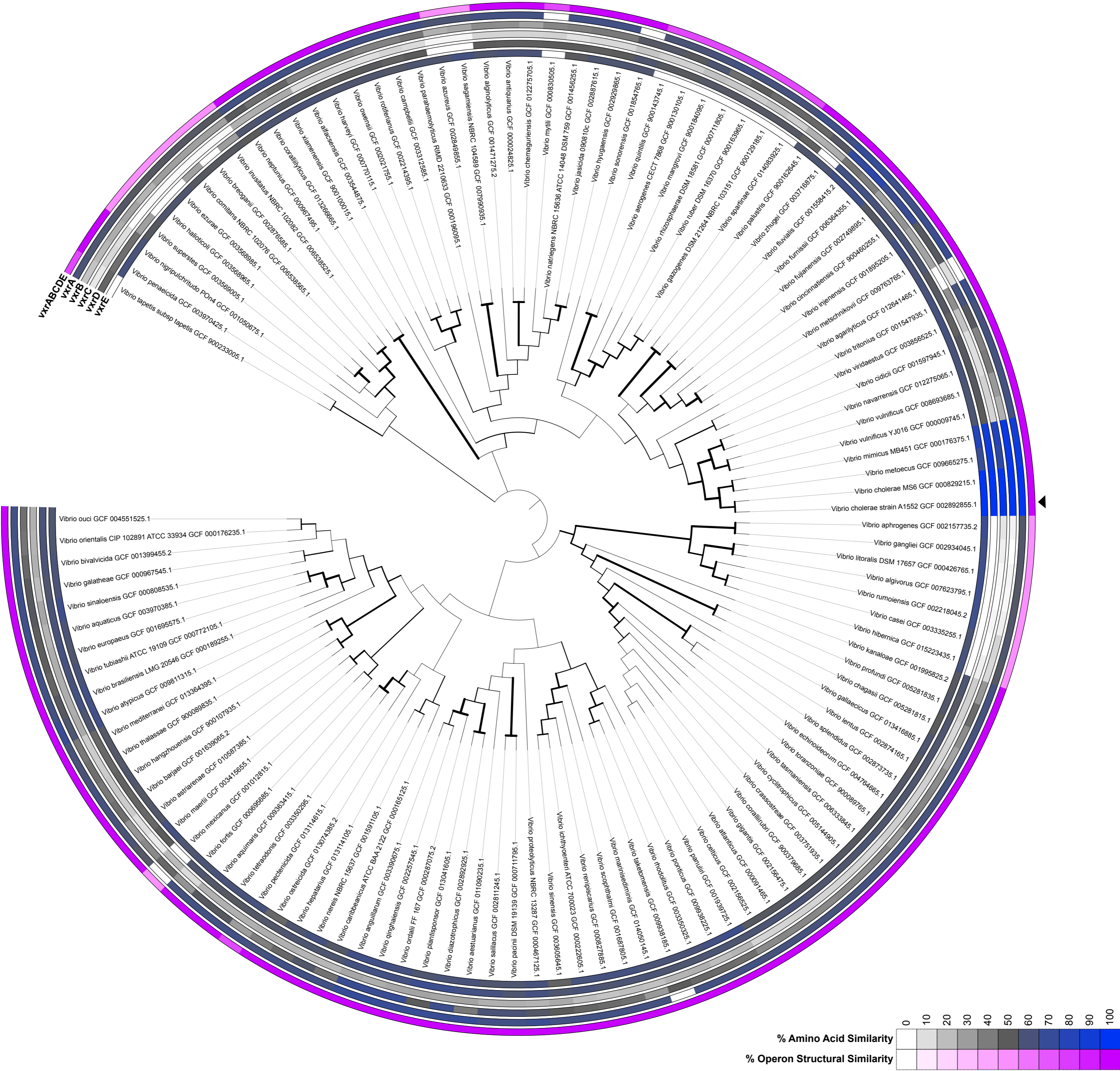

Supplement: S2 Fig — The conservation of vxrABCDE genes across the Vibrio genus was assessed and structural similarity scores and individual gene percent identities were annotated on a RecA reference phylogeny using the iTOL web service [62]. Amino acid % similarity of Rvv homologs is shown for each protein encoded in the rvv loci. % similarity is shown as a gradient from grey to blue, with blue representing the highest similarity. On the outermost ring, structural similarity of the rvv genomic region is visualized as a gradient from white to purple, with purple representing the highest structural similarity. (PDF) [file ppat.1011415.s002.pdf]

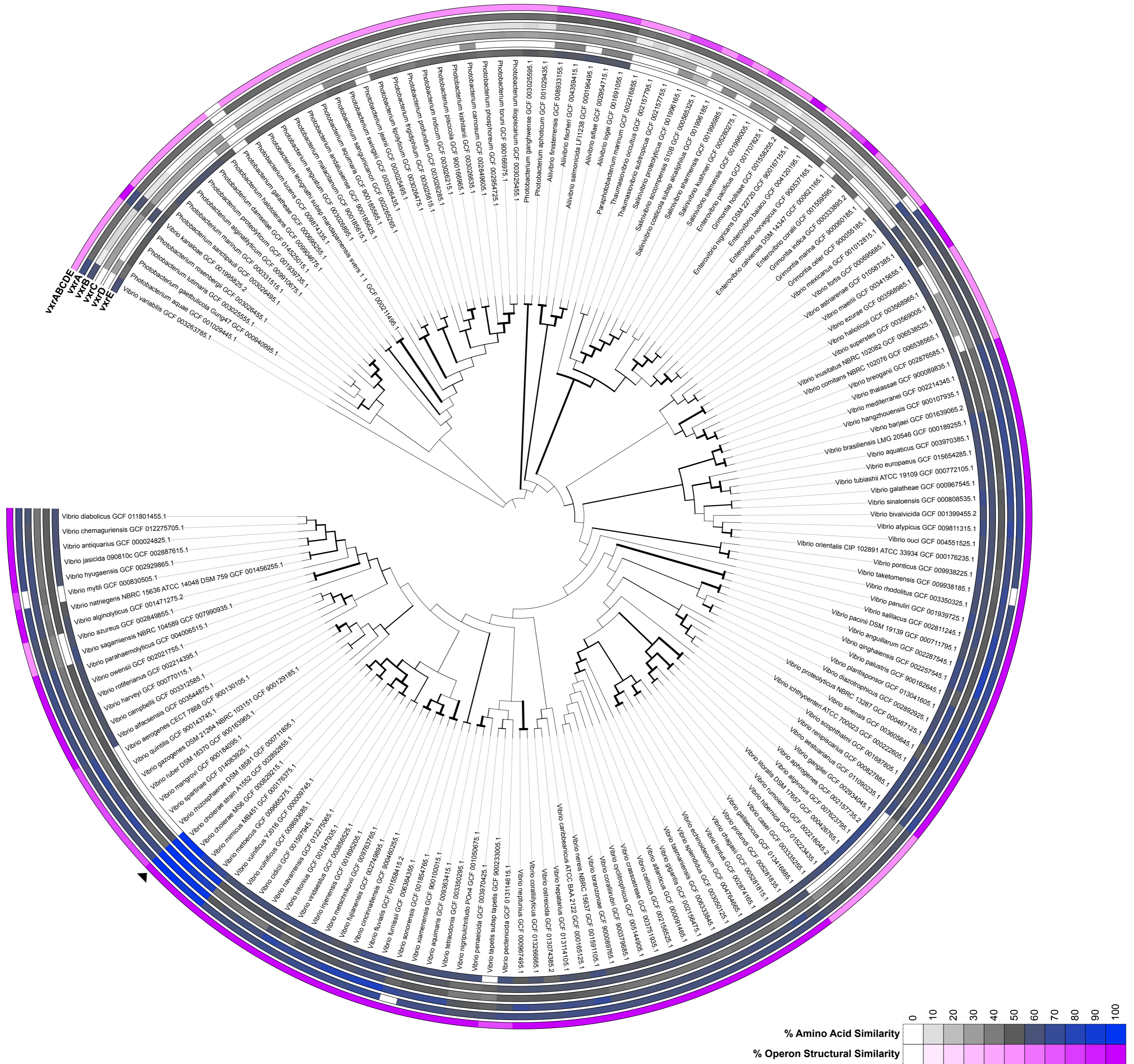

Supplement: S3 Fig — The conservation of vxrABCDE genes across the Vibrionales order was assessed and structural similarity scores and individual gene percent identities were annotated on a RecA reference phylogeny using the iTOL web service [62]. Amino acid % similarity of Rvv homologs is shown for each protein encoded in the rvv loci. % similarity is shown as a gradient from grey to blue, with blue representing the highest similarity. On the outermost ring, structural similarity of the rvv genomic region is visualized as a gradient from white to purple, with purple representing the highest structural similarity. (PDF) [file ppat.1011415.s003.pdf]

**A**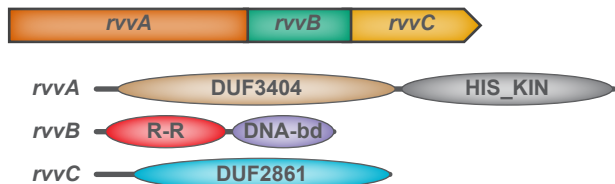**B**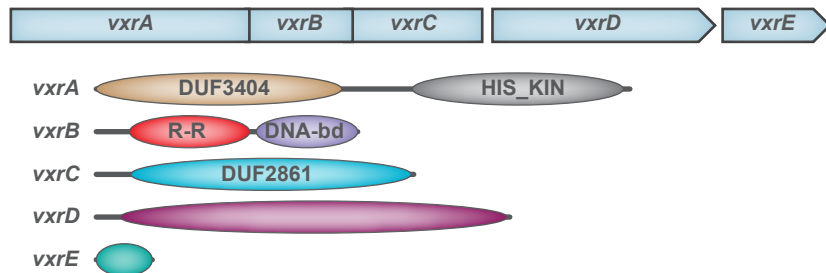**C**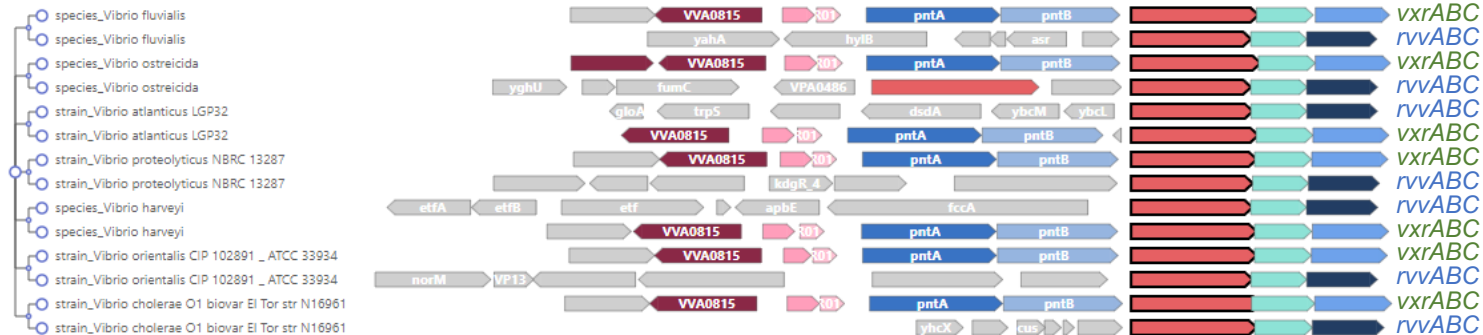

Supplement: S4 Fig — (A-B) Genomic organization (top) and predicted domains per protein (bottom) of the Rvv (A) and Vxr (B) TCSs in V. cholerae. (C) GeCoViz rendition of the surrounding genomic region of rvv and vxr in representative Vibrio species [65]. (PDF) [file ppat.1011415.s004.pdf]

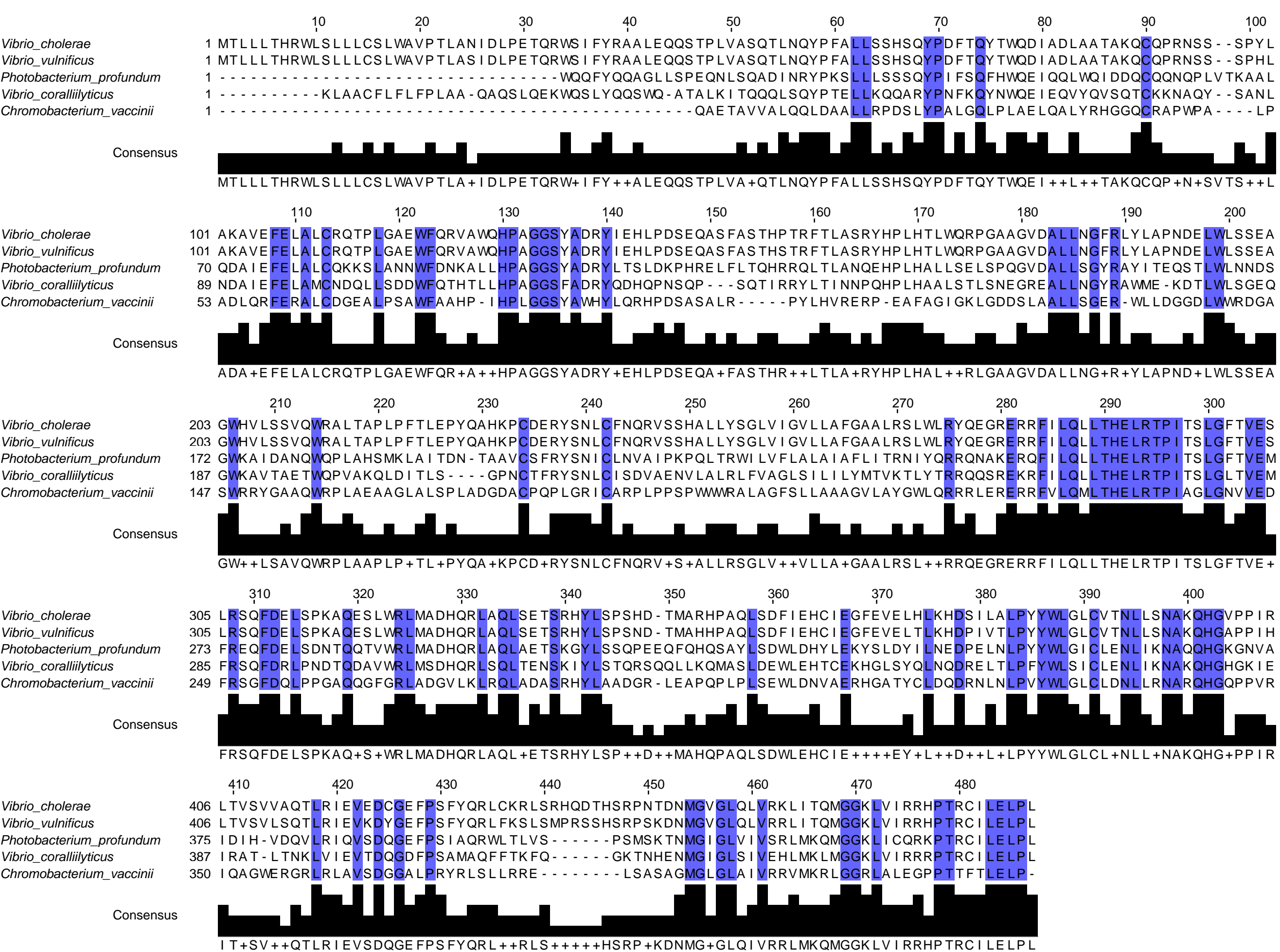

Supplement: S6 Fig — BlastP sequence alignment of RvvA and homologous proteins from selected high % identity species. Residues with 100% conservation across all selected species are shown in blue. (PDF) [file ppat.1011415.s006.pdf]

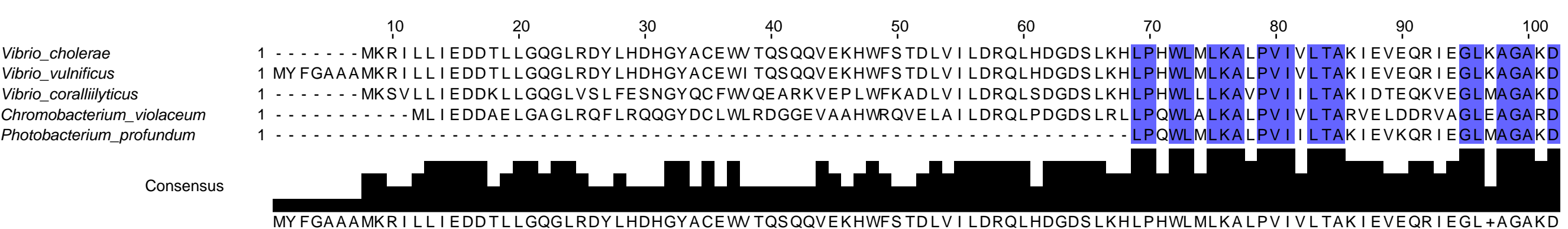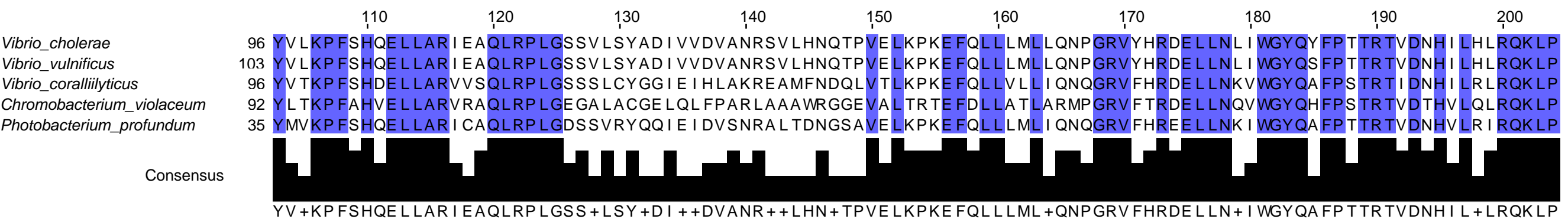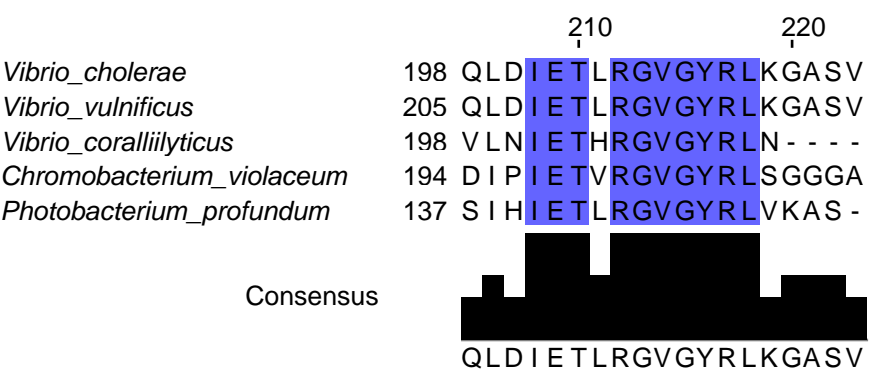

Supplement: S7 Fig — BlastP sequence alignment of RvvB and homologous proteins from selected high % identity species. Residues with 100% conservation across all selected species are shown in blue. (PDF) [file ppat.1011415.s007.pdf]

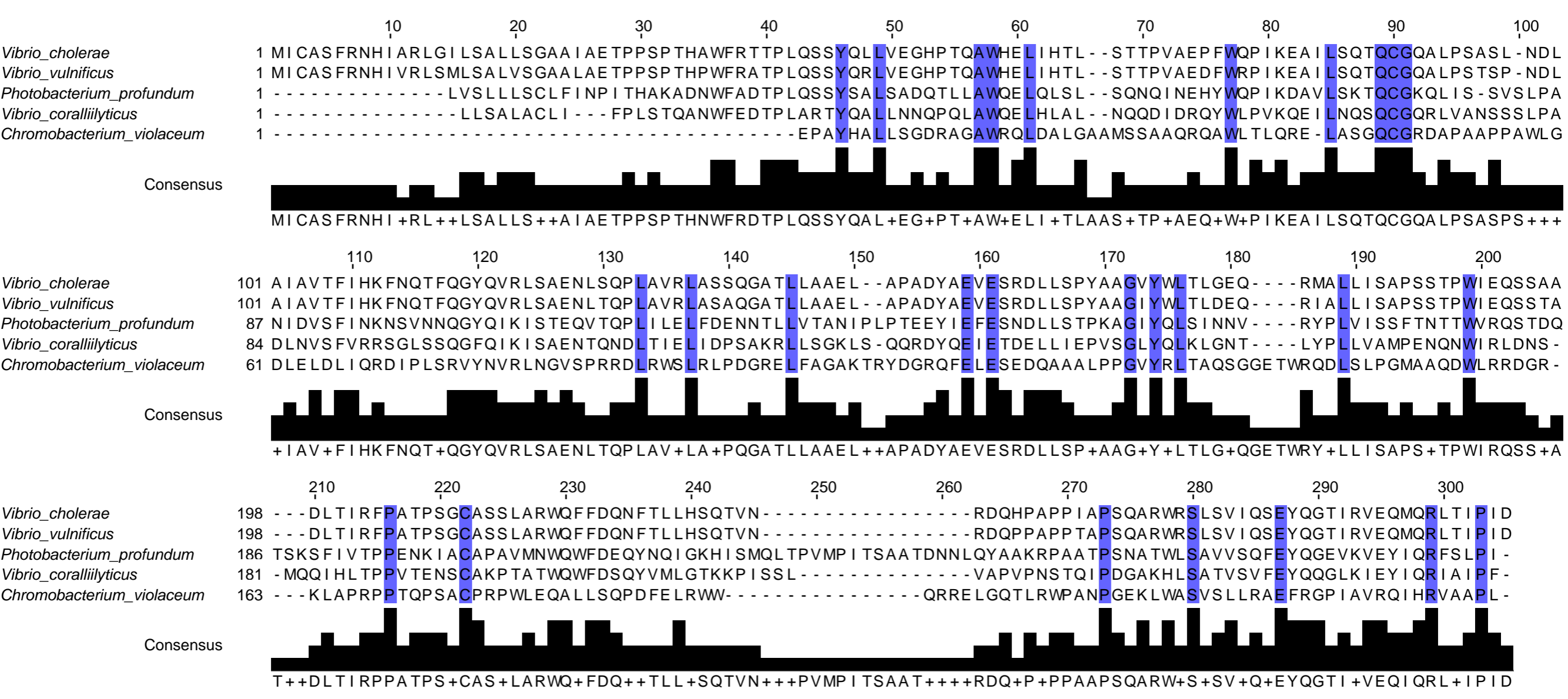

Supplement: S8 Fig — BlastP sequence alignment of RvvC and homologous proteins from selected high % identity species. Residues with 100% conservation across all selected species are shown in blue. (PDF) [file ppat.1011415.s008.pdf]
