# Supplementary material for: The Rvv two-component regulatory system regulates biofilm formation and colonization in Vibrio cholerae
Source: PLoS Pathog. 2023 May 22;19(5):e1011415. doi: 10.1371/journal.ppat.1011415 (PMC10237652; doi:10.1371/journal.ppat.1011415)
Supplement: S5 Fig — ClustalO Amino acid sequence alignment (right) and phylogenetic tree (left) of the (A) REC domain of OmpR-like response regulators in V. cholerae and (B) the HisKA DHp domain of Classic-type histidine kinases (HKs) in V. cholerae. For the HKs, the regions of the domain with conservation are shown, corresponding to regions 278–317 and 318–346 of RvvA’s amino acid sequence. Phylogenetic trees were generated from sequence alignment via neighbor-joining using BLOSUM62. (PDF) [file ppat.1011415.s005.pdf]

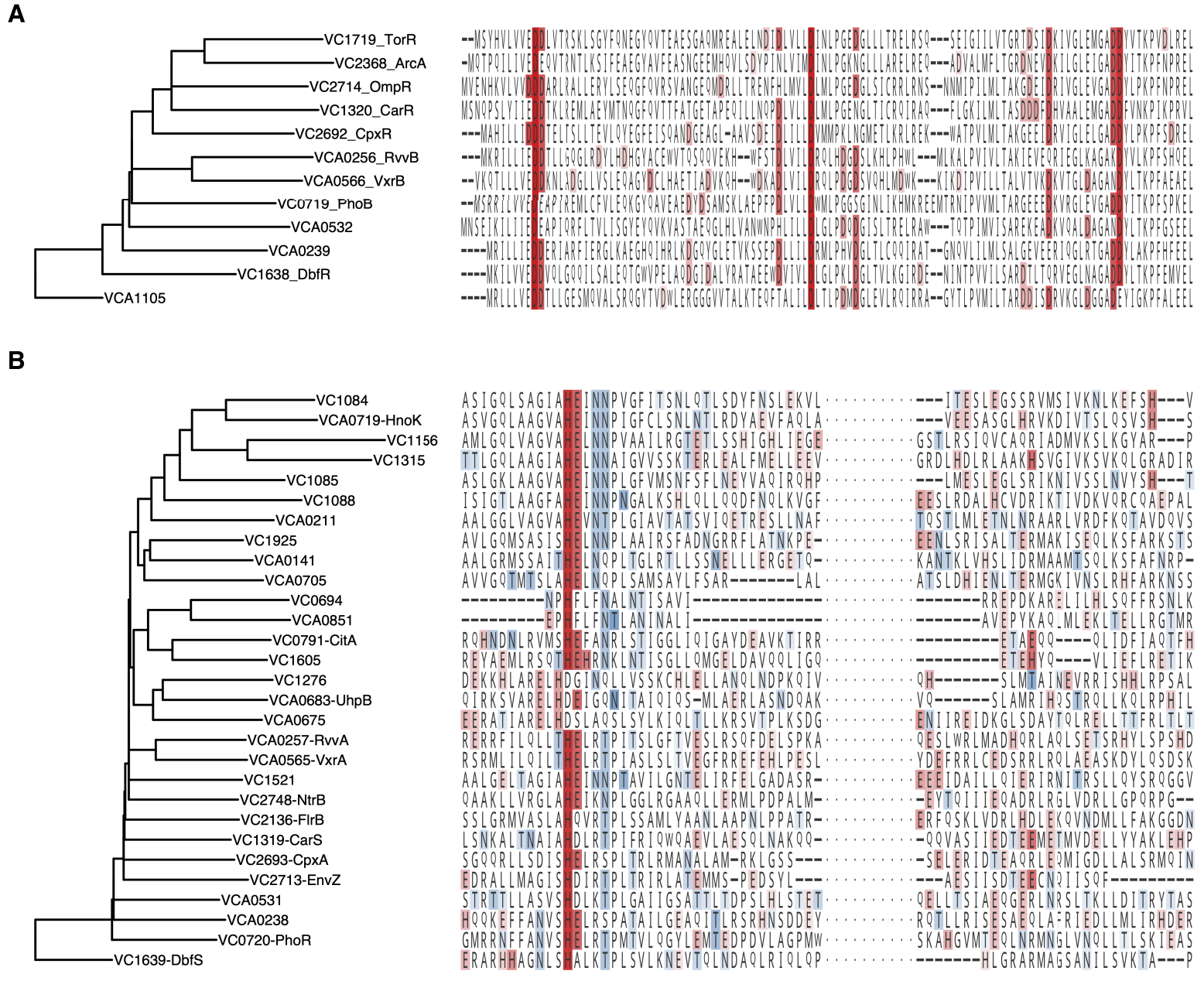
A
VC1719_TorR
VC2368_ArcA
VC2714_OmpR
VC1320_CarR
VC2692_CpxR
VCA0256_RvvB
VCA0566_VxrB
VC0719_PhoB
VCA0532
VCA0239
VC1638_DbfR
VCA1105
B
VC1084
VCA0719-HnoK
VC1156
VC1315
VC1085
VC1088
VCA0211
VC1925
VCA0141
VCA0705
VC0694
VCA0851
VC0791-CitA
VC1605
VC1276
VCA0683-UhpB
VCA0675
VCA0257-RvvA
VCA0565-VxrA
VC1521
VC2748-NtrB
VC2136-FlrB
VC1319-CarS
VC2693-CpxA
VC2713-EnvZ
VCA0531
VCA0238
VC0720-PhoR
VC1639-DbfS
