# Supplementary material for: The Rvv two-component regulatory system regulates biofilm formation and colonization in Vibrio cholerae
Source: PLoS Pathog. 2023 May 22;19(5):e1011415. doi: 10.1371/journal.ppat.1011415 (PMC10237652; doi:10.1371/journal.ppat.1011415)
Supplement: S1 Table — (PDF) [file ppat.1011415.s009.pdf]

S1 Table. Strains and plasmids used.		
**Strain or plasmid**	**Relevant properties**	**Source**
***E. coli* strains**		
CC118λ*pir*	Δ(*ara-leu*)*araD* Δ*lacX74 galE galK phoA20 thi-1 rpsE rpoB argE*(Am) *recA1* λ*pir*	[1]
DH5α-λ*pir*	*endA1 hsdR17 glnV44 (= supE44) thi-1 recA1 gyrA96 relA1 φ80dlac*Δ(*lacZ*)M15 Δ(*lacZYA-argF*)U169 *zdg-232*::Tn10 *uidA::pir+*	[2]
S17-1λ*pir*	Tp^r Sm^r *recA thi pro* r_K^- m_K^+ RP4::2-Tc::MuKm Tn*7* λ*pir*	[3]
***V. cholerae* strains**		
FY_Vc_1	*Vibrio cholerae* O1 El Tor A1552, wild type, Rif^r	[4]
FY_Vc_3	Δ*lacZ*, Rif^r	[5]
FY_Vc_237	FY_Vc_1 Tn*7::gfp* Rif^r Gm^r	[6]
FY_Vc_2272	Δ*vpsR* (VC0665), Rif^r	[7]
FY_Vc_99	Δ*vpsT* (VCA0952), Rif^r	[8]
FY_Vc_183	Δ*hapR* (VC0583), Rif^r	[9]
FY_Vc_3411	Δ*vps*-I (*vpsA-K*; VC0917-27) Δ*vps*-II (*vpsL-Q*; VC0934-39), Rif^r	[10]
FY_Vc_10884	Δ*rvvA* (VCA0257), Rif^r	This study
FY_Vc_12787	Δ*rvvA* (VCA0257) Tn7::*rvvA*, Rif^r	This study
FY_Vc_8148	Δ*rvvB* (VCA0256), Rif^r	This study
FY_Vc_12081	Δ*rvvC* (VCA0255), Rif^r	This study
FY_Vc_14853	Δ*rvvA* Δ*rvvC*, Rif^r	This study
FY_Vc_12084	Δ*rvvA* Δ*rvvB*, Rif^r	This study
FY_Vc_16140	Δ*vps*-I Δ*vps*-II Δ*rvvA*, Rif^r	This study
FY_Vc_11814	Δ*vpsR* Δ*rvvA*, Rif^r	This study
FY_Vc_13576	Δ*vpsT* Δ*rvvA*, Rif^r	This study
FY_Vc_10876	Δ*hapR* Δ*rvvA*, Rif^r	This study

FY_Vc_17945	*rvvB*^{D57A}, Rif^{r}	This study
FY_Vc_17947	*rvvB*^{D57E}, Rif^{r}	This study
FY_Vc_17738	Δ*rvvA*, *rvvB*^{D57A}, Rif^{r}	This study
FY_Vc_17740	Δ*rvvA*, *rvvB*^{D57E}, Rif^{r}	This study
FY_Vc_16721	*rvvA*^{H289A}, Rif^{r}	This study
FY_Vc_16723	*rvvA*^{E290A}, Rif^{r}	This study
FY_Vc_17949	*rvvA*^{T293A}, Rif^{r}	This study
FY_Vc_12244	Δ*rvvD* (VCA0254), Rif^{r}	This study
FY_Vc_12245	Δ*rvvE* (VCA0258), Rif^{r}	This study
FY_Vc_14715	Δ*rvvA* Δ*rvvD*, Rif^{r}	This study
FY_Vc_17364	Δ*rvvA* Δ*rvvE*, Rif^{r}	This study
**Plasmids**		
pGP704-*sacB*28	pGP704 derivative; *mob-oriT sacB*, Ap^{r}	[10]
pMCM11	pGP704::mTn7-*gfp*, Gm^{r} Ap^{r}	[10]
pUX-BF13	oriR6K helper plasmid, *mob-oriT*, provides the Tn7 transposition function in *trans*, Ap^{r}	[11]
pBBRlux	*luxCDABE*-based promoter fusion vector, Cm^{r}	[12]
pFY_3406	pBBR*lux vpsL* promoter, Cm^{r} (-607, +158)	[13]
pFY_0989	pBBR*lux vpsR* promoter, Cm^{r}	[14]
pFY_0988	pBBR*lux vpsT* promoter, Cm^{r}	[14]
pFY_1038	pBBR*lux ctxA* promoter, Cm^{r}	[14]
pFY_951	pBBR*lux tcpA* promoter, Cm^{r}	[14]
pFY_5921	pBBR*lux rvvA* promoter, Cm^{r} (-102, +9)	This study
pFY_5925	pBBR*lux rvvE* promoter, Cm^{r} (-150, +9)	This study
pFY_5927	pBBR*lux rvvD* promoter, Cm^{r} (-500, +9)	This study

References:

1. Herrero M, de Lorenzo V, Timmis KN. Transposon vectors containing non-antibiotic resistance selection markers for cloning and stable chromosomal insertion of foreign genes in gram-negative bacteria. Journal of Bacteriology 1990;172:6557–67.

2. Platt R, Drescher C, Park SK, Phillips GJ. Genetic System for Reversible Integration of DNA Constructs and lacZ Gene Fusions into the Escherichia coli Chromosome. Plasmid 2000;43:12–23.

3. Simon R, Priefer U, Pühler A. A broad host range mobilization system for in vivo genetic engineering: Transposon mutagenesis in gram negative bacteria. Bio/Technology 1983;1:784–91.

4. Yildiz FH, Schoolnik GK. *Vibrio cholerae* O1 El Tor: Identification of a gene cluster required for the rugose colony type, exopolysaccharide production, chlorine resistance, and biofilm formation. Proc. Natl. Acad. Sci. U.S.A. 1999;96:4028–33.

5. Casper-Lindley C, Yildiz FH. VpsT Is a Transcriptional Regulator Required for Expression of *vps* Biosynthesis Genes and the Development of Rugose Colonial Morphology in *Vibrio cholerae* O1 El Tor. Journal of Bacteriology 2004;186:1574–8.

6. Beyhan S, Tischler AD, Camilli A, Yildiz FH. Transcriptome and phenotypic responses of Vibrio cholerae to increased cyclic di-GMP level. Journal of bacteriology 2006;188:3600–13.

7. Yildiz FH, Dolganov NA, Schoolnik GK. VpsR, a Member of the Response Regulators of the Two-Component Regulatory Systems, Is Required for Expression of *vps* Biosynthesis Genes and EPS ^{ETr} -Associated Phenotypes in *Vibrio cholerae* O1 El Tor. J Bacteriol 2001;183:1716–26.

8. Kitts G, Giglio KM, Zamorano-Sánchez D, Park JH, Townsley L, Cooley RB, et al. A Conserved Regulatory Circuit Controls Large Adhesins in Vibrio cholerae. mBio 2019;10:e02822-19.

9. Beyhan S, Bilecen K, Salama SR, Casper-Lindley C, Yildiz FH. Regulation of Rugosity and Biofilm Formation in Vibrio cholerae: Comparison of VpsT and VpsR Regulons and Epistasis Analysis of vpsT, vpsR, and hapR. Journal of Bacteriology 2007;189:388–402.

10. Fong JCN, Yildiz FH. Interplay between Cyclic AMP-Cyclic AMP Receptor Protein and Cyclic di-GMP Signaling in *Vibrio cholerae* Biofilm Formation. J Bacteriol 2008;190:6646–59.

11. Bao Y, Lies DP, Fu H, Roberts GP. An improved Tn7-based system for the single-copy insertion of cloned genes into chromosomes of gram-negative bacteria. Gene 1991;109:167–8.

12. Lenz DH, Mok KC, Lilley BN, Kulkarni RV, Wingreen NS, Bassler BL. The Small RNA Chaperone Hfq and Multiple Small RNAs Control Quorum Sensing in Vibrio harveyi and Vibrio cholerae. Cell 2004;118:69–82.

13. Zamorano-Sánchez D, Fong JCN, Kilic S, Erill I, Yildiz FH. Identification and Characterization of VpsR and VpsT Binding Sites in *Vibrio cholerae*. J. Bacteriol. 2015;197:1221–35.

14. Rogers A, Townsley L, Gallego-Hernandez AL, Beyhan S, Kwuan L, Yildiz FH. The LonA Protease Regulates Biofilm Formation, Motility, Virulence, and the Type VI Secretion System in Vibrio cholerae. J Bacteriol 2016;198:973–85.
