# Supplementary material for: The Rvv two-component regulatory system regulates biofilm formation and colonization in Vibrio cholerae
Source: PLoS Pathog. 2023 May 22;19(5):e1011415. doi: 10.1371/journal.ppat.1011415 (PMC10237652; doi:10.1371/journal.ppat.1011415)
Supplement: S1 Data — (PDF) [file ppat.1011415.s010.pdf]

**Supplementary Data 1:** Operon Structure Similarity Calculation

Consider the reference operon, $O_{ref}\colon\{ABCDE\}$ ,where *A*, *B*, *C*, *D* and *E* are genes.

Consider also the following detected operons in a target *GenomeAssembly*:

*Operon*, $O_j$, from *NucleotideRecord*, $f_{i=1}$, from *GenomeAssembly*, $S_{k=1}$:

$$O_{j=1}\colon\{ABC\}\ and\ O_{j=2}\colon\{DE\}$$

*Operon*, $O_j$, from *NucleotideRecord*, $f_{i=2}$, from *GenomeAssembly*, $S_{k=1}$:

$$O_{j=1}\colon\{DE\}$$

The gene-pairs, $P_{ref}$, in the reference operon $O_{ref}$ are:

$$P_{ref}\colon[AB, BC, CD, DE]$$

The observed gene-pairs, $P_i$, for the nucleotide records *NucleotideRecord*, *i*, mapping to genome assembly *GenomeAssembly*, $S_{k=1}$ are:

$$P_{i=1}\colon[AB, BC, DE]$$

$$P_{i=2}\colon[DE]$$

The maximum number of times each reference pair could *possibly* occur based off the number of occurrences of each gene, $M$, in *GenomeAssembly*, $S_{k=1}$ is:

$$M_{[AB]} \to 1$$

$$M_{[BC]} \to 1$$

$$M_{[CD]} \to 1$$

$$M_{[DE]} \to 2$$

The *observed* number of occurrences, $T$, for each gene-pair $P$, in *GenomeAssembly*, $S_{k=1}$:

$$T_{[AB]} \to 1$$

$$T_{[BC]} \to 1$$

$$T_{[CD]} \to 0$$

$$T_{[DE]} \to 2$$

Therefore, the relative number of occurrences, $R$, for each pair is $T/M$:

$$R_{[AB]} = \frac{T_{[AB]}}{M_{[AB]}} = 1$$

$$R_{[BC]} = \frac{T_{[BC]}}{M_{[BC]}} = 1$$

$$R_{[CD]} = \frac{T_{[CD]}}{M_{[CD]}} = 0$$

$$R_{[DE]} = \frac{T_{[DE]}}{M_{[DE]}} = 1$$

The total number of relative occurrences is then:

$$\sum R = R_{[AB]} + R_{[BC]} + R_{[CD]} + R_{[DE]} = 3$$

The proportion of relative occurrences to the number of gene-pairs $P_{ref}$ in the reference operon is the structural conservation score $Y$:

$$Y = \frac{\sum R}{P_{ref}} = \frac{3}{4} = 0.75$$
