# Supplementary material for: The Rvv two-component regulatory system regulates biofilm formation and colonization in Vibrio cholerae
Source: PLoS Pathog. 2023 May 22;19(5):e1011415. doi: 10.1371/journal.ppat.1011415 (PMC10237652; doi:10.1371/journal.ppat.1011415)

Vibrio_cholerae_MS6_GCF_000829215.1								
**Structural Similarity**	**Average Percent Amino Acid Identity**	**WP_001911723.1**	**WP_000815041.1**	**WP_000240569.1**	**WP_000822678.1**	**WP_000459082.1**	**Taxonomic ID**	**Genome Assembly Accession**
100.0%	100.0%	100.0%	100.0%	100.0%	100.0%	100.0%	1420885	GCF_000829215.1

Other Gene ■ VxrA ■ VxrB ■ VxrC ■ VxrD ■ VxrE ■

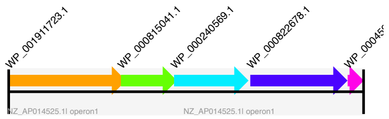

Vibrio_metoecus_GCF_009665275.1								
**Structural Similarity**	**Average Percent Amino Acid Identity**	**WP_001911723.1**	**WP_000815041.1**	**WP_000240569.1**	**WP_000822678.1**	**WP_000459082.1**	**Taxonomic ID**	**Genome Assembly Accession**
100.0%	87.83267536640349%	94.3733004363774%	98.62385321100918%	83.53965795197922%	93.31150722937834%	69.3150580032734%	1481663	GCF_009665275.1

Other Gene ■ VxrA ■ VxrB ■ VxrC ■ VxrD ■ VxrE ■

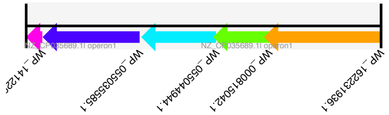

Vibrio_mimicus_MB451_GCF_000176375.1								
**Structural Similarity**	**Average Percent Amino Acid Identity**	**WP_001911723.1**	**WP_000815041.1**	**WP_000240569.1**	**WP_000822678.1**	**WP_000459082.1**	**Taxonomic ID**	**Genome Assembly Accession**
100.0%	89.75775088145619%	90.21956087824351%	98.62385321100918%	87.4704807815503%	91.61765022532133%	80.85720931115667%	675806	GCF_000176375.1
Other Gene ■ VxrA ■ VxrB ■ VxrC ■ VxrD ■ VxrE ■								

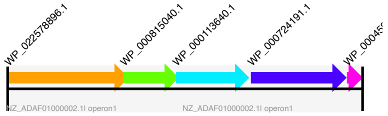

Vibrio_fluvialis_GCF_001558415.2								
**Structural Similarity**	**Average Percent Amino Acid Identity**	**WP_001911723.1**	**WP_000815041.1**	**WP_000240569.1**	**WP_000822678.1**	**WP_000459082.1**	**Taxonomic ID**	**Genome Assembly Accession**
100.0%	70.27394976713853%	70.04614245037479%	92.17048366431774%	61.49253731343284%	63.36222893459242%	64.29835647297489%	676	GCF_001558415.2

Other Gene VxrA VxrB VxrC VxrD VxrE

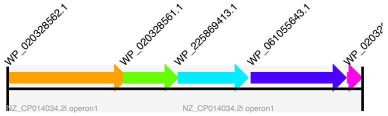

Vibrio_furnissii_GCF_006364355.1								
**Structural Similarity**	**Average Percent Amino Acid Identity**	**WP_001911723.1**	**WP_000815041.1**	**WP_000240569.1**	**WP_000822678.1**	**WP_000459082.1**	**Taxonomic ID**	**Genome Assembly Accession**
100.0%	70.39871755512428%	69.67747854274123%	93.27354260089686%	59.940652818991104%	63.191615834459334%	65.91029797853292%	29494	GCF_006364355.1

Other Gene | VxrA | VxrB | VxrC | VxrD | VxrE

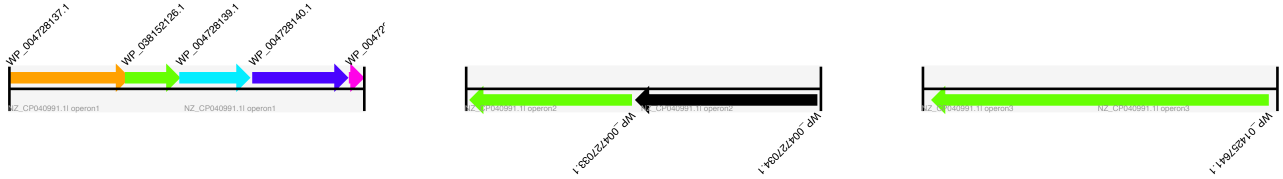

Vibrio_anguillarum_GCF_002287545.1								
**Structural Similarity**	**Average Percent Amino Acid Identity**	**WP_001911723.1**	**WP_000815041.1**	**WP_000240569.1**	**WP_000822678.1**	**WP_000459082.1**	**Taxonomic ID**	**Genome Assembly Accession**
100.0%	68.24683796438033%	70.62146892655367%	91.51968117062526%	51.665384812620964%	62.729341143438056%	64.69831376866375%	55601	GCF_002287545.1

Other Gene VxrA VxrB VxrC VxrD VxrE

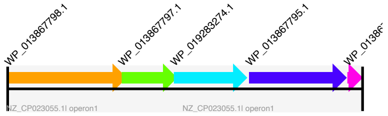

Vibrio_qinghaiensis_GCF_002257545.1								
**Structural Similarity**	**Average Percent Amino Acid Identity**	**WP_001911723.1**	**WP_000815041.1**	**WP_000240569.1**	**WP_000822678.1**	**WP_000459082.1**	**Taxonomic ID**	**Genome Assembly Accession**
100.0%	68.05727458292849%	70.18096538205748%	91.27653826953154%	50.2352722739450%	63.89528322044463%	64.69831376866375%	2025808	GCF_002257545.1
Other Gene	VxrA	VxrB	VxrC	VxrD	VxrE			

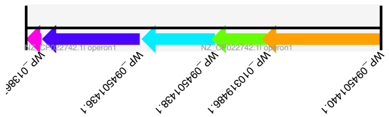

Vibrio_aestuarianus_GCF_011090235.1								
**Structural Similarity**	**Average Percent Amino Acid Identity**	**WP_001911723.1**	**WP_000815041.1**	**WP_000240569.1**	**WP_000822678.1**	**WP_000459082.1**	**Taxonomic ID**	**Genome Assembly Accession**
100.0%	66.1443194413904%	70.64831204067382%	83.68200836820083%	52.46398850749381%	63.91895264179224%	60.00833564879132%	28171	GCF_011090235.1

Other Gene | VxrA | VxrB | VxrC | VxrD | VxrE

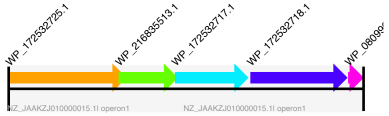

Vibrio_ordalii_ATCC_33509_GCF_000257205.1								
**Structural Similarity**	**Average Percent Amino Acid Identity**	**WP_001911723.1**	**WP_000815041.1**	**WP_000240569.1**	**WP_000822678.1**	**WP_000459082.1**	**Taxonomic ID**	**Genome Assembly Accession**
100.0%	67.6007290450855%	69.80666690001985%	91.27653826953154%	50.235272273945%	64.11849861832941%	62.56666916360175%	990998	GCF_000257205.1

Other Gene ■ VxrA ■ VxrB ■ VxrC ■ VxrD ■ VxrE ■

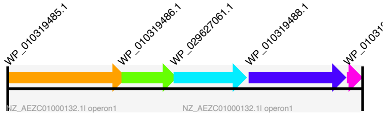

Vibrio_plantisponsor_GCF_013041605.1								
**Structural Similarity**	**Average Percent Amino Acid Identity**	**WP_001911723.1**	**WP_000815041.1**	**WP_000240569.1**	**WP_000822678.1**	**WP_000459082.1**	**Taxonomic ID**	**Genome Assembly Accession**
100.0%	66.56999555506911%	69.25954340447296%	86.07594936708861%	52.453568870852216%	67.34941380442982%	57.71150232850197%	664643	GCF_013041605.1

Other Gene VxrA VxrB VxrC VxrD VxrE

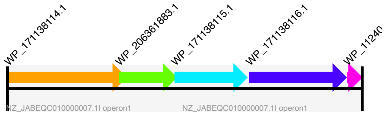

Vibrio_diazotrophicus_GCF_002892925.1								
**Structural Similarity**	**Average Percent Amino Acid Identity**	**WP_001911723.1**	**WP_000815041.1**	**WP_000240569.1**	**WP_000822678.1**	**WP_000459082.1**	**Taxonomic ID**	**Genome Assembly Accession**
100.0%	67.35120612794577%	68.5714815123637%	90.62680623237524%	51.87895382872251%	66.31838667123682%	59.3604023950305076%	685	GCF_002892925.1

Other Gene VxrA VxrB VxrC VxrD VxrE

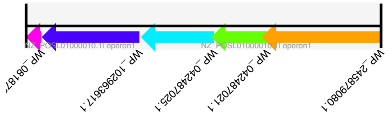

Vibrio_injenensis_GCF_001895205.1								
**Structural Similarity**	**Average Percent Amino Acid Identity**	**WP_001911723.1**	**WP_000815041.1**	**WP_000240569.1**	**WP_000822678.1**	**WP_000459082.1**	**Taxonomic ID**	**Genome Assembly Accession**
100.0%	65.7817531720291%	67.77163904235728%	86.71690046530617%	52.25858867223769%	58.46153846153847%	63.70009921870591%	1307414	GCF_001895205.1

Other Gene VxrA VxrB VxrC VxrD VxrE

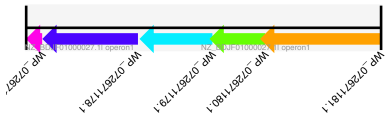

Vibrio_metschnikovii_GCF_009763765.1								
**Structural Similarity**	**Average Percent Amino Acid Identity**	**WP_001911723.1**	**WP_000815041.1**	**WP_000240569.1**	**WP_000822678.1**	**WP_000459082.1**	**Taxonomic ID**	**Genome Assembly Accession**
100.0%	64.89101185627277%	68.02218114602589%	86.65323132028455%	52.0777621333551%	57.86026200873362%	59.841622672964704%	28172	GCF_009763765.1
Other Gene VxrA VxrB VxrC VxrD VxrE								

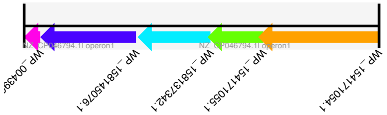

Vibrio_tritonius_GCF_001547935.1								
**Structural Similarity**	**Average Percent Amino Acid Identity**	**WP_001911723.1**	**WP_000815041.1**	**WP_000240569.1**	**WP_000822678.1**	**WP_000459082.1**	**Taxonomic ID**	**Genome Assembly Accession**
100.0%	65.41342991718568%	66.30036630036629%	89.04850170421226%	56.69515669515669%	53.48016755130123%	61.54295733489189%	1435069	GCF_001547935.1

Other Gene VxrA VxrB VxrC VxrD VxrE

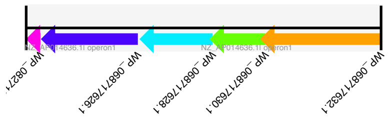

Vibrio_cincinnatiensis_GCF_900460255.1								
**Structural Similarity**	**Average Percent Amino Acid Identity**	**WP_001911723.1**	**WP_000815041.1**	**WP_000240569.1**	**WP_000822678.1**	**WP_000459082.1**	**Taxonomic ID**	**Genome Assembly Accession**
100.0%	65.88595247785234%	65.11627906976744%	91.07142857142857%	55.88856121175843%	59.88093038285576%	57.47256315345153%	675	GCF_900460255.1

Other Gene ■ VxrA ■ VxrB ■ VxrC ■ VxrD ■ VxrE ■

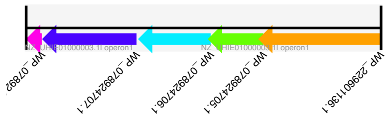

Vibrio_proteolyticus_NBRC_13287_GCF_000467125.1								
**Structural Similarity**	**Average Percent Amino Acid Identity**	**WP_001911723.1**	**WP_000815041.1**	**WP_000240569.1**	**WP_000822678.1**	**WP_000459082.1**	**Taxonomic ID**	**Genome Assembly Accession**
100.0%	65.62092789570556%	67.62426198094329%	87.32066367744493%	49.276383291057826%	62.866381376539394%	61.016949152542374%	1219065	GCF_000467125.1

Other Gene VxrA VxrB VxrC VxrD VxrE

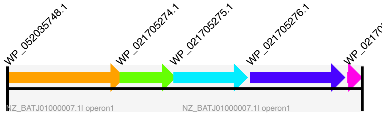

Vibrio_bivalvicida_GCF_001399455.2								
**Structural Similarity**	**Average Percent Amino Acid Identity**	**WP_001911723.1**	**WP_000815041.1**	**WP_000240569.1**	**WP_000822678.1**	**WP_000459082.1**	**Taxonomic ID**	**Genome Assembly Accession**
100.0%	64.09539061628301%	66.57481414562204%	84.90275133293665%	49.95376782599774%	59.71506259782103%	59.33055717903761%	1276888	GCF_001399455.2
Other Gene	VxrA	VxrB	VxrC	VxrD	VxrE			

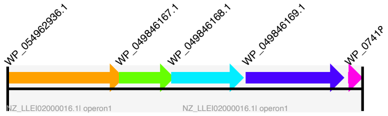

Vibrio_fujianensis_GCF_002749895.1								
**Structural Similarity**	**Average Percent Amino Acid Identity**	**WP_001911723.1**	**WP_000815041.1**	**WP_000240569.1**	**WP_000822678.1**	**WP_000459082.1**	**Taxonomic ID**	**Genome Assembly Accession**
100.0%	64.93184464284226%	66.78765880217786%	90.86904761904762%	52.974716813911414%	60.78813805054708%	53.23966192852735%	1974215	GCF_002749895.1

Other Gene | VxrA | VxrB | VxrC | VxrD | VxrE

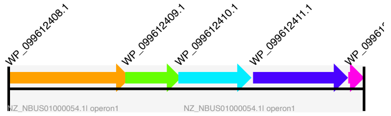

Vibrio_atypicus_GCF_009811315.1

Structural Similarity	Average Percent Amino Acid Identity	WP_001911723.1	WP_000815041.1	WP_000240569.1	WP_000822678.1	WP_000459082.1	Taxonomic ID	Genome Assembly Accession
100.0%	65.88755095218288%	68.11749605792423%	88.05395934314883%	51.05199383124034%	57.915949055626136%	64.29835647297489%	558271	GCF_009811315.1

Other Gene | VxrA | VxrB | VxrC | VxrD | VxrE

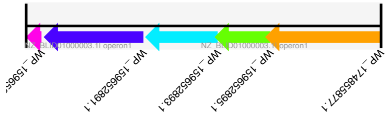

Vibrio_europaeus_GCF_015654285.1								
**Structural Similarity**	**Average Percent Amino Acid Identity**	**WP_001911723.1**	**WP_000815041.1**	**WP_000240569.1**	**WP_000822678.1**	**WP_000459082.1**	**Taxonomic ID**	**Genome Assembly Accession**
100.0%	64.32786604696281%	64.20317801625502%	85.71552784229979%	50.58566867164653%	61.97202304664786%	59.16293265796482%	300876	GCF_015654285.1

Other Gene | VxrA | VxrB | VxrC | VxrD | VxrE

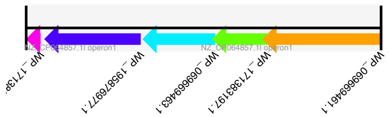

Vibrio_tubiashii_ATCC_19109_GCF_000772105.1								
**Structural Similarity**	**Average Percent Amino Acid Identity**	**WP_001911723.1**	**WP_000815041.1**	**WP_000240569.1**	**WP_000822678.1**	**WP_000459082.1**	**Taxonomic ID**	**Genome Assembly Accession**
100.0%	64.02062627612034%	63.854914647939175%	85.27596103285207%	50.4203658740503%	60.04341524948901%	60.508474576271176%	1051646	GCF_000772105.1

Other Gene VxrA VxrB VxrC VxrD VxrE

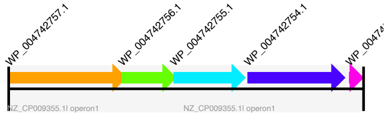

Vibrio_xiamenensis_GCF_900100015.1								
**Structural Similarity**	**Average Percent Amino Acid Identity**	**WP_001911723.1**	**WP_000815041.1**	**WP_000240569.1**	**WP_000822678.1**	**WP_000459082.1**	**Taxonomic ID**	**Genome Assembly Accession**
100.0%	62.63658492570933%	64.8946466377659%	85.12667715288596%	50.9196832648905%	52.533665280700845%	59.7082522923034%	861298	GCF_900100015.1

Other Gene VxrA VxrB VxrC VxrD VxrE

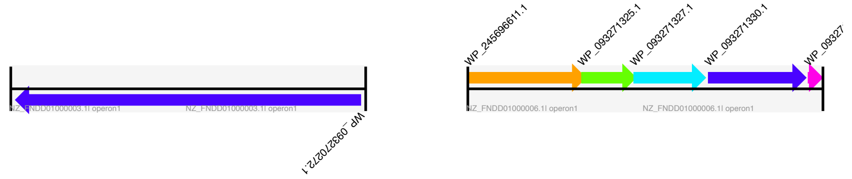

Vibrio_galatheae_GCF_000967545.1								
**Structural Similarity**	**Average Percent Amino Acid Identity**	**WP_001911723.1**	**WP_000815041.1**	**WP_000240569.1**	**WP_000822678.1**	**WP_000459082.1**	**Taxonomic ID**	**Genome Assembly Accession**
100.0%	63.280264301515295%	66.26347879406316%	86.47264595596911%	47.36128365387708%	56.97335592462949%	59.33055717903761%	579748	GCF_000967545.1

Other Gene | VxrA | VxrB | VxrC | VxrD | VxrE

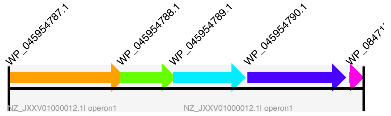

Vibrio_aquaticus_GCF_003970385.1								
**Structural Similarity**	**Average Percent Amino Acid Identity**	**WP_001911723.1**	**WP_000815041.1**	**WP_000240569.1**	**WP_000822678.1**	**WP_000459082.1**	**Taxonomic ID**	**Genome Assembly Accession**
100.0%	64.11180065069742%	65.93834369912203%	85.74049708763995%	49.15985343264982%	60.389751855037666%	59.330557179037626%	2496559	GCF_003970385.1

Other Gene VxrA VxrB VxrC VxrD VxrE

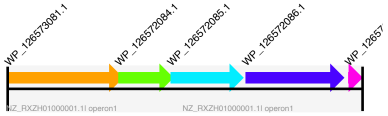

Vibrio_aestivus_GCF_003263845.1								
**Structural Similarity**	**Average Percent Amino Acid Identity**	**WP_001911723.1**	**WP_000815041.1**	**WP_000240569.1**	**WP_000822678.1**	**WP_000459082.1**	**Taxonomic ID**	**Genome Assembly Accession**
100.0%	62.698223584853096%	64.7912885662432%	85.17792202972456%	47.86676957069063%	57.79994650077654%	57.855191256830594%	1117706	GCF_003263845.1

Other Gene VxrA VxrB VxrC VxrD VxrE

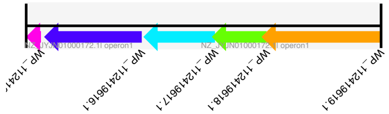

Vibrio_neptunius_GCF_000967495.1								
**Structural Similarity**	**Average Percent Amino Acid Identity**	**WP_001911723.1**	**WP_000815041.1**	**WP_000240569.1**	**WP_000822678.1**	**WP_000459082.1**	**Taxonomic ID**	**Genome Assembly Accession**
100.0%	61.170863540262005%	63.50626118067979%	86.38332672669854%	49.427489310010785%	56.12073645061363%	50.4165040333073%	170651	GCF_000967495.1

Other Gene | VxrA | VxrB | VxrC | VxrD | VxrE

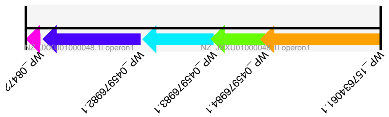

Vibrio_brasiliensis_LMG_20546_GCF_000189255.1								
**Structural Similarity**	**Average Percent Amino Acid Identity**	**WP_001911723.1**	**WP_000815041.1**	**WP_000240569.1**	**WP_000822678.1**	**WP_000459082.1**	**Taxonomic ID**	**Genome Assembly Accession**
100.0%	65.4949012921493%	63.76811594202898%	86.66971276868468%	50.25703367252605%	62.79368805165396%	63.98595602585275%	945543	GCF_000189255.1

Other Gene | VxrA | VxrB | VxrC | VxrD | VxrE

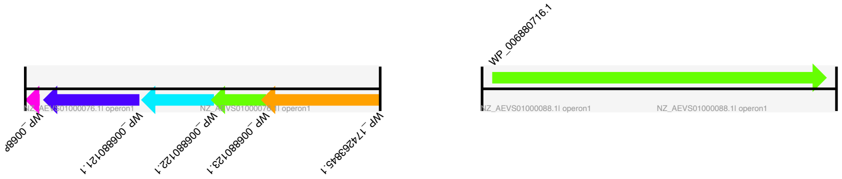

Vibrio_harveyi_GCF_000770115.1								
**Structural Similarity**	**Average Percent Amino Acid Identity**	**WP_001911723.1**	**WP_000815041.1**	**WP_000240569.1**	**WP_000822678.1**	**WP_000459082.1**	**Taxonomic ID**	**Genome Assembly Accession**
100.0%	59.04162262554803%	65.01831501831502%	83.1912008308446%	41.6156607167689%	48.92397892622788%	56.458957635583786%	669	GCF_000770115.1

Other Gene | VxrA | VxrB | VxrC | VxrD | VxrE

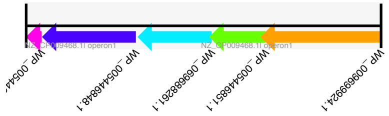

Vibrio_owensii_GCF_002021755.1								
**Structural Similarity**	**Average Percent Amino Acid Identity**	**WP_001911723.1**	**WP_000815041.1**	**WP_000240569.1**	**WP_000822678.1**	**WP_000459082.1**	**Taxonomic ID**	**Genome Assembly Accession**
100.0%	59.15023355286926%	65.01831501831502%	83.62224332219625%	41.283185583151685%	49.82383961129268%	56.00358422939068%	696485	GCF_002021755.1

Other Gene | VxrA | VxrB | VxrC | VxrD | VxrE

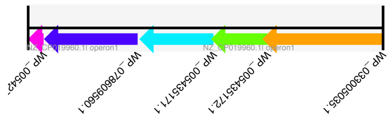

Vibrio_sinaloensis_GCF_000808535.1								
**Structural Similarity**	**Average Percent Amino Acid Identity**	**WP_001911723.1**	**WP_000815041.1**	**WP_000240569.1**	**WP_000822678.1**	**WP_000459082.1**	**Taxonomic ID**	**Genome Assembly Accession**
100.0%	66.19852770924092%	65.74844846195359%	86.78029177379308%	51.812665594707674%	59.97430332330362%	66.67692939244662%	379097	GCF_000808535.1

Other Gene | VxrA | VxrB | VxrC | VxrD | VxrE

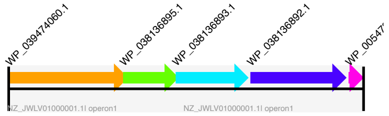

Vibrio_campbellii_GCF_003312585.1								
**Structural Similarity**	**Average Percent Amino Acid Identity**	**WP_001911723.1**	**WP_000815041.1**	**WP_000240569.1**	**WP_000822678.1**	**WP_000459082.1**	**Taxonomic ID**	**Genome Assembly Accession**
100.0%	58.36762161769629%	63.934426229508205%	82.76015833949319%	42.593859988096156%	48.14618170854718%	54.403481822836675%	680	GCF_003312585.1

Other Gene ■ VxrA ■ VxrB ■ VxrC ■ VxrD ■ VxrE ■

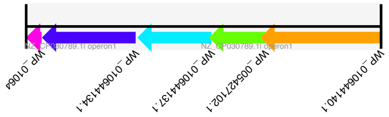

Vibrio_toranzoniae_GCF_900089765.1								
**Structural Similarity**	**Average Percent Amino Acid Identity**	**WP_001911723.1**	**WP_000815041.1**	**WP_000240569.1**	**WP_000822678.1**	**WP_000459082.1**	**Taxonomic ID**	**Genome Assembly Accession**
100.0%	59.25565194615626%	62.23021582733813%	81.13948927116674%	43.48991087344029%	56.92482233067901%	52.49382142815718%	1194427	GCF_900089765.1

Other Gene VxrA VxrB VxrC VxrD VxrE

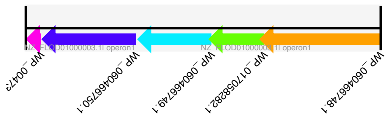

Vibrio_aquimaris_GCF_009363415.1								
**Structural Similarity**	**Average Percent Amino Acid Identity**	**WP_001911723.1**	**WP_000815041.1**	**WP_000240569.1**	**WP_000822678.1**	**WP_000459082.1**	**Taxonomic ID**	**Genome Assembly Accession**
100.0%	63.17497610125116%	63.68613138686131%	86.70689910616385%	49.76446172969925%	54.93113282233977%	60.78625546119163%	2587862	GCF_009363415.1

Other Gene VxrA VxrB VxrC VxrD VxrE

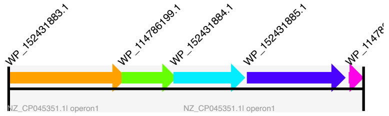

Vibrio_tetraodonis_GCF_003350295.1								
**Structural Similarity**	**Average Percent Amino Acid Identity**	**WP_001911723.1**	**WP_000815041.1**	**WP_000240569.1**	**WP_000822678.1**	**WP_000459082.1**	**Taxonomic ID**	**Genome Assembly Accession**
100.0%	62.67378730340273%	63.68613138686131%	86.70689910616385%	49.6241479005862%	52.56550266221062%	60.78625546119163%	2231647	GCF_003350295.1
Other Gene ■ VxrA ■ VxrB ■ VxrC ■ VxrD ■ VxrE ■								

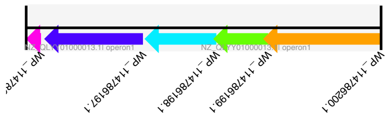

Vibrio_kanaloae_GCF_001995825.2								
**Structural Similarity**	**Average Percent Amino Acid Identity**	**WP_001911723.1**	**WP_000815041.1**	**WP_000240569.1**	**WP_000822678.1**	**WP_000459082.1**	**Taxonomic ID**	**Genome Assembly Accession**
100.0%	59.35177236556781%	62.34234234234234%	81.13948927116674%	43.640283687943274%	57.14292509822949%	52.49382142815718%	170673	GCF_001995825.2

Other Gene | VxrA | VxrB | VxrC | VxrD | VxrE

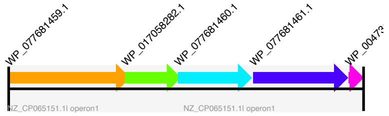

Vibrio_cyclitrophicus_GCF_005144905.1								
**Structural Similarity**	**Average Percent Amino Acid Identity**	**WP_001911723.1**	**WP_000815041.1**	**WP_000240569.1**	**WP_000822678.1**	**WP_000459082.1**	**Taxonomic ID**	**Genome Assembly Accession**
100.0%	59.319278218272906%	62.05035971223022%	82.22735776383844%	42.39077905846411%	56.92482233067901%	53.00307222615276%	47951	GCF_005144905.1

Other Gene ■ VxrA ■ VxrB ■ VxrC ■ VxrD ■ VxrE ■

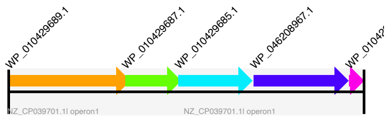

Vibrio_hyugaensis_GCF_002929865.1								
**Structural Similarity**	**Average Percent Amino Acid Identity**	**WP_001911723.1**	**WP_000815041.1**	**WP_000240569.1**	**WP_000822678.1**	**WP_000459082.1**	**Taxonomic ID**	**Genome Assembly Accession**
100.0%	59.52676477451248%	64.46886446886447%	83.62224332219625%	41.91237699353137%	50.02665245202558%	57.60368663594472%	1534743	GCF_002929865.1

Other Gene | VxrA | VxrB | VxrC | VxrD | VxrE

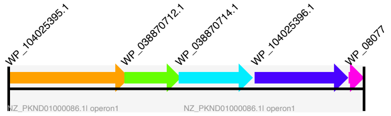

Vibrio_pacinii_DSM_19139_GCF_000711795.1								
**Structural Similarity**	**Average Percent Amino Acid Identity**	**WP_001911723.1**	**WP_000815041.1**	**WP_000240569.1**	**WP_000822678.1**	**WP_000459082.1**	**Taxonomic ID**	**Genome Assembly Accession**
100.0%	63.4380123686178%	64.41605839416059%	88.64274449784476%	49.45914866494676%	57.51714147296075%	57.154968813176076%	1123494	GCF_000711795.1

Other Gene VxrA VxrB VxrC VxrD VxrE

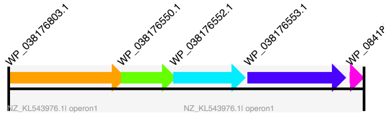

Vibrio_coralliilyticus_GCF_013266665.1								
**Structural Similarity**	**Average Percent Amino Acid Identity**	**WP_001911723.1**	**WP_000815041.1**	**WP_000240569.1**	**WP_000822678.1**	**WP_000459082.1**	**Taxonomic ID**	**Genome Assembly Accession**
100.0%	62.91579230946011%	62.87744227353463%	84.39682741395673%	50.67116657592844%	58.762511462442404%	57.871013821438424%	190893	GCF_013266665.1

Other Gene ■ VxrA ■ VxrB ■ VxrC ■ VxrD ■ VxrE ■

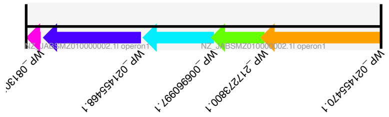

Vibrio_pectenicida_GCF_013114615.1								
**Structural Similarity**	**Average Percent Amino Acid Identity**	**WP_001911723.1**	**WP_000815041.1**	**WP_000240569.1**	**WP_000822678.1**	**WP_000459082.1**	**Taxonomic ID**	**Genome Assembly Accession**
75.0%	62.22012169520011%	64.88484774350506%	80.41666666666667%	49.97639436512408%	54.79698374512322%	61.025715955581525%	62763	GCF_013114615.1

Other Gene VxrA VxrB VxrC VxrD VxrE

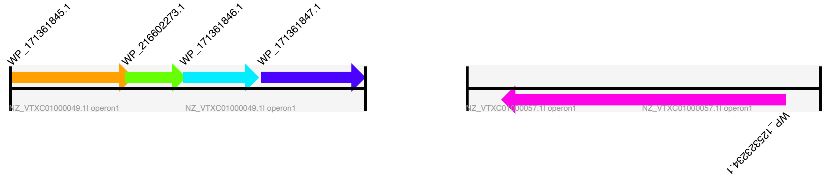

Vibrio_jasicida_090810c_GCF_002887615.1								
**Structural Similarity**	**Average Percent Amino Acid Identity**	**WP_001911723.1**	**WP_000815041.1**	**WP_000240569.1**	**WP_000822678.1**	**WP_000459082.1**	**Taxonomic ID**	**Genome Assembly Accession**
100.0%	59.44195139519449%	64.46886446886447%	83.62224332219625%	41.91237699353137%	49.6025855554356%	57.60368663594472%	1280002	GCF_002887615.1

Other Gene ■ VxrA ■ VxrB ■ VxrC ■ VxrD ■ VxrE ■

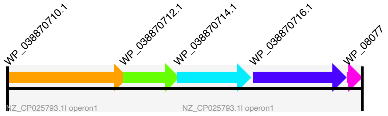

Vibrio_orientalis_CIP_102891_ATCC_33934_GCF_000176235.1								
**Structural Similarity**	**Average Percent Amino Acid Identity**	**WP_001911723.1**	**WP_000815041.1**	**WP_000240569.1**	**WP_000822678.1**	**WP_000459082.1**	**Taxonomic ID**	**Genome Assembly Accession**
100.0%	62.57516508427926%	63.1578947368421%	83.94450659424099%	49.12145724616545%	59.17940369069623%	57.47256315345153%	675816	GCF_000176235.1

Other Gene VxrA VxrB VxrC VxrD VxrE

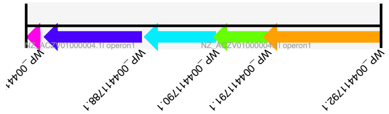

**Vibrio_coralliirubri_GCF_900379685.1**								
**Structural Similarity**	**Average Percent Amino Acid Identity**	**WP_001911723.1**	**WP_000815041.1**	**WP_000240569.1**	**WP_000822678.1**	**WP_000459082.1**	**Taxonomic ID**	**Genome Assembly Accession**
100.0%	58.94949707617966%	61.87050359712231%	82.22735776383844%	42.077304964539%	56.46043572573798%	52.11188332966056%	1516159	GCF_900379685.1
Other Gene ■ VxrA ■ VxrB ■ VxrC ■ VxrD ■ VxrE ■								

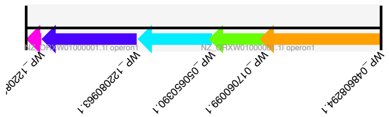

Vibrio_ponticus_GCF_009938225.1								
**Structural Similarity**	**Average Percent Amino Acid Identity**	**WP_001911723.1**	**WP_000815041.1**	**WP_000240569.1**	**WP_000822678.1**	**WP_000459082.1**	**Taxonomic ID**	**Genome Assembly Accession**
100.0%	63.19484124410836%	62.824427147487135%	84.5703426108016%	46.9104825072413%	58.40722258831331%	63.261731366698434%	265668	GCF_009938225.1

Other Gene VxrA VxrB VxrC VxrD VxrE

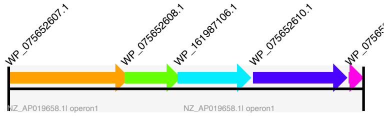

Vibrio_gigantis_GCF_002156475.1								
**Structural Similarity**	**Average Percent Amino Acid Identity**	**WP_001911723.1**	**WP_000815041.1**	**WP_000240569.1**	**WP_000822678.1**	**WP_000459082.1**	**Taxonomic ID**	**Genome Assembly Accession**
100.0%	59.86190351519447%	61.87050359712231%	82.3283510062036%	43.25907218240308%	59.3577693620862%	52.49382142815718%	296199	GCF_002156475.1
Other Gene	VxrA	VxrB	VxrC	VxrD	VxrE			

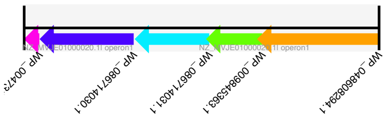

Vibrio_parahaemolyticus_GCF_004006515.1								
**Structural Similarity**	**Average Percent Amino Acid Identity**	**WP_001911723.1**	**WP_000815041.1**	**WP_000240569.1**	**WP_000822678.1**	**WP_000459082.1**	**Taxonomic ID**	**Genome Assembly Accession**
100.0%	59.54548450376457%	64.33823529411765%	83.9124846228383%	41.21552343405311%	50.34523648190812%	57.915942685905655%	670	GCF_004006515.1

Other Gene | VxrA | VxrB | VxrC | VxrD | VxrE

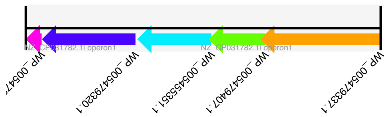

Vibrio_hepatarius_GCF_013114105.1								
**Structural Similarity**	**Average Percent Amino Acid Identity**	**WP_001911723.1**	**WP_000815041.1**	**WP_000240569.1**	**WP_000822678.1**	**WP_000459082.1**	**Taxonomic ID**	**Genome Assembly Accession**
100.0%	62.97820969722657%	64.98716505767453%	84.83639422340438%	50.04688095283393%	57.87371559685277%	57.14689265536723%	171383	GCF_013114105.1

Other Gene VxrA VxrB VxrC VxrD VxrE

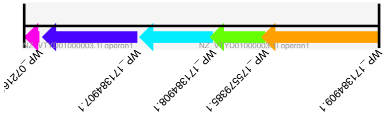

Vibrio_ouci_GCF_004551525.1								
**Structural Similarity**	**Average Percent Amino Acid Identity**	**WP_001911723.1**	**WP_000815041.1**	**WP_000240569.1**	**WP_000822678.1**	**WP_000459082.1**	**Taxonomic ID**	**Genome Assembly Accession**
100.0%	62.823146830987085%	63.1578947368421%	84.31202368349439%	49.95402431813434%	59.21922826301304%	57.47256315345153%	2499078	GCF_004551525.1

Other Gene ■ VxrA ■ VxrB ■ VxrC ■ VxrD ■ VxrE ■

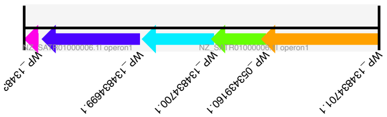

Vibrio_alfacsensis_GCF_003544875.1								
**Structural Similarity**	**Average Percent Amino Acid Identity**	**WP_001911723.1**	**WP_000815041.1**	**WP_000240569.1**	**WP_000822678.1**	**WP_000459082.1**	**Taxonomic ID**	**Genome Assembly Accession**
100.0%	59.407023508639725%	63.503649635036496%	82.83414386768372%	41.35227045906784%	50.14126453891181%	59.20378904249872%	1074311	GCF_003544875.1
Other Gene	VxrA	VxrB	VxrC	VxrD	VxrE			

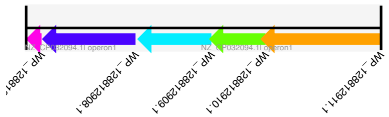

Vibrio_renipiscarius_GCF_000827885.1								
**Structural Similarity**	**Average Percent Amino Acid Identity**	**WP_001911723.1**	**WP_000815041.1**	**WP_000240569.1**	**WP_000822678.1**	**WP_000459082.1**	**Taxonomic ID**	**Genome Assembly Accession**
100.0%	62.790789049353485%	63.55228208070255%	84.59989273744857%	45.52845528455284%	57.55244041193802%	62.720874732125445%	1461322	GCF_000827885.1

Other Gene VxrA VxrB VxrC VxrD VxrE

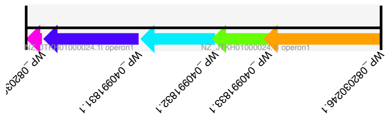

Vibrio_salilacus_GCF_002811245.1								
**Structural Similarity**	**Average Percent Amino Acid Identity**	**WP_001911723.1**	**WP_000815041.1**	**WP_000240569.1**	**WP_000822678.1**	**WP_000459082.1**	**Taxonomic ID**	**Genome Assembly Accession**
100.0%	64.34777865982517%	64.29229106339824%	88.64274449784476%	49.473461710033135%	62.65585680399124%	56.674539223858474%	1323749	GCF_002811245.1

Other Gene VxrA VxrB VxrC VxrD VxrE

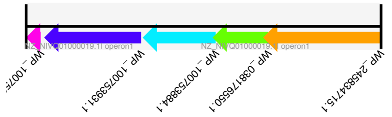

Vibrio_scophthalmi_GCF_001687805.1								
**Structural Similarity**	**Average Percent Amino Acid Identity**	**WP_001911723.1**	**WP_000815041.1**	**WP_000240569.1**	**WP_000822678.1**	**WP_000459082.1**	**Taxonomic ID**	**Genome Assembly Accession**
100.0%	63.03976744594083%	63.264722040526344%	84.5335075174066%	47.361490685126064%	58.01533616843149%	62.02378081821366%	45658	GCF_001687805.1

Other Gene | VxrA | VxrB | VxrC | VxrD | VxrE

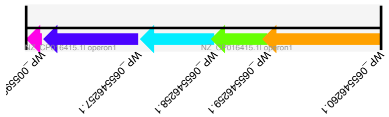

Vibrio_panuliri_GCF_001939725.1								
**Structural Similarity**	**Average Percent Amino Acid Identity**	**WP_001911723.1**	**WP_000815041.1**	**WP_000240569.1**	**WP_000822678.1**	**WP_000459082.1**	**Taxonomic ID**	**Genome Assembly Accession**
100.0%	64.000073716381%	63.52087114337568%	84.38431095933703%	48.860305234493914%	57.14292722170986%	66.0919540229885%	1381081	GCF_001939725.1

Other Gene VxrA VxrB VxrC VxrD VxrE

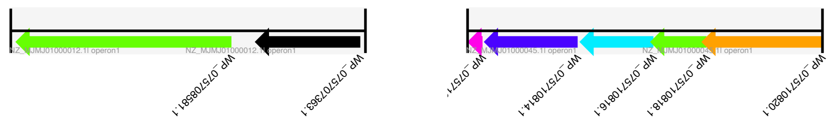

Vibrio_ostreicida_GCF_013074385.2								
**Structural Similarity**	**Average Percent Amino Acid Identity**	**WP_001911723.1**	**WP_000815041.1**	**WP_000240569.1**	**WP_000822678.1**	**WP_000459082.1**	**Taxonomic ID**	**Genome Assembly Accession**
100.0%	59.3984208404799%	61.741472561202016%	82.97101453801304%	50.55903128398942%	55.55580920898938%	46.16477661020564%	526588	GCF_013074385.2

Other Gene VxrA VxrB VxrC VxrD VxrE

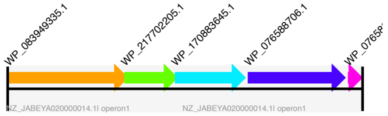

Vibrio_rotiferianus_GCF_002214395.1								
**Structural Similarity**	**Average Percent Amino Acid Identity**	**WP_001911723.1**	**WP_000815041.1**	**WP_000240569.1**	**WP_000822678.1**	**WP_000459082.1**	**Taxonomic ID**	**Genome Assembly Accession**
100.0%	58.9293732746573%	63.58595194085028%	82.81039159352349%	40.633245382585756%	51.613693226936306%	56.00358422939068%	190895	GCF_002214395.1

Other Gene | VxrA | VxrB | VxrC | VxrD | VxrE

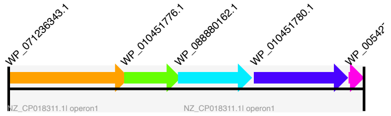

Vibrio_tasmaniensis_GCF_006333845.1								
**Structural Similarity**	**Average Percent Amino Acid Identity**	**WP_001911723.1**	**WP_000815041.1**	**WP_000240569.1**	**WP_000822678.1**	**WP_000459082.1**	**Taxonomic ID**	**Genome Assembly Accession**
100.0%	59.425955678688446%	61.759425493716336%	82.3283510062036%	41.86418552978752%	58.683994935577566%	52.49382142815718%	212663	GCF_006333845.1

Other Gene VxrA VxrB VxrC VxrD VxrE

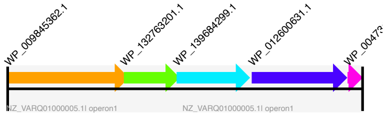

Vibrio_chagasii_GCF_005281815.1								
**Structural Similarity**	**Average Percent Amino Acid Identity**	**WP_001911723.1**	**WP_000815041.1**	**WP_000240569.1**	**WP_000822678.1**	**WP_000459082.1**	**Taxonomic ID**	**Genome Assembly Accession**
100.0%	58.59184333226833%	61.98198198198198%	81.79909027548514%	41.88905525846703%	56.488616795578025%	50.80047234982948%	170679	GCF_005281815.1
Other Gene ■ VxrA ■ VxrB ■ VxrC ■ VxrD ■ VxrE ■								

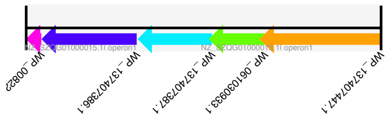

Vibrio_rhodolitus_GCF_003350325.1								
**Structural Similarity**	**Average Percent Amino Acid Identity**	**WP_001911723.1**	**WP_000815041.1**	**WP_000240569.1**	**WP_000822678.1**	**WP_000459082.1**	**Taxonomic ID**	**Genome Assembly Accession**
100.0%	62.93198089588019%	63.40120533402227%	84.91777304080587%	49.93016842428211%	57.08020050125313%	59.33055717903761%	2231649	GCF_003350325.1

Other Gene | VxrA | VxrB | VxrC | VxrD | VxrE

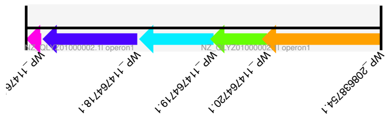

Vibrio_atlanticus_GCF_000091465.1								
**Structural Similarity**	**Average Percent Amino Acid Identity**	**WP_001911723.1**	**WP_000815041.1**	**WP_000240569.1**	**WP_000822678.1**	**WP_000459082.1**	**Taxonomic ID**	**Genome Assembly Accession**
100.0%	59.47861503155609%	61.759425493716336%	82.3283510062036%	42.1274822941258%	58.683994935577566%	52.49382142815718%	693153	GCF_000091465.1

Other Gene | VxrA | VxrB | VxrC | VxrD | VxrE

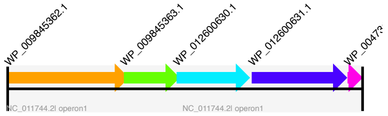

Vibrio_chemaguriensis_GCF_012275705.1								
**Structural Similarity**	**Average Percent Amino Acid Identity**	**WP_001911723.1**	**WP_000815041.1**	**WP_000240569.1**	**WP_000822678.1**	**WP_000459082.1**	**Taxonomic ID**	**Genome Assembly Accession**
100.0%	58.96873140209544%	63.78676470588235%	79.86914022856133%	42.38130617817726%	49.07460493782922%	59.731840960027014%	2527672	GCF_012275705.1
Other Gene VxrA VxrB VxrC VxrD VxrE								

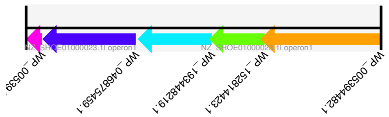

Vibrio_diabolicus_GCF_011801455.1								
**Structural Similarity**	**Average Percent Amino Acid Identity**	**WP_001911723.1**	**WP_000815041.1**	**WP_000240569.1**	**WP_000822678.1**	**WP_000459082.1**	**Taxonomic ID**	**Genome Assembly Accession**
100.0%	59.30918242492081%	63.78676470588235%	81.66373545350663%	42.07743759779926%	49.286133407388824%	59.731840960027014%	50719	GCF_011801455.1

Other Gene | VxrA | VxrB | VxrC | VxrD | VxrE

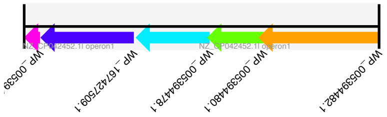

Vibrio_antiquarius_GCF_000024825.1								
**Structural Similarity**	**Average Percent Amino Acid Identity**	**WP_001911723.1**	**WP_000815041.1**	**WP_000240569.1**	**WP_000822678.1**	**WP_000459082.1**	**Taxonomic ID**	**Genome Assembly Accession**
100.0%	59.30918242492081%	63.78676470588235%	81.66373545350663%	42.07743759779926%	49.286133407388824%	59.731840960027014%	150340	GCF_000024825.1

Other Gene | VxrA | VxrB | VxrC | VxrD | VxrE

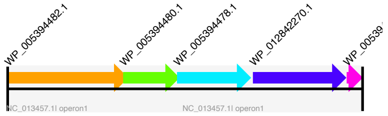

Vibrio_echinoideorum_GCF_004764665.1								
**Structural Similarity**	**Average Percent Amino Acid Identity**	**WP_001911723.1**	**WP_000815041.1**	**WP_000240569.1**	**WP_000822678.1**	**WP_000459082.1**	**Taxonomic ID**	**Genome Assembly Accession**
100.0%	60.03978618343966%	61.469534050179206%	83.01113659908243%	43.558654772375114%	57.51358073172329%	54.6460247638383%	2100116	GCF_004764665.1

Other Gene VxrA VxrB VxrC VxrD VxrE

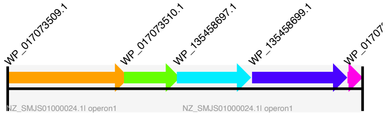

Vibrio_celticus_GCF_002156525.1								
**Structural Similarity**	**Average Percent Amino Acid Identity**	**WP_001911723.1**	**WP_000815041.1**	**WP_000240569.1**	**WP_000822678.1**	**WP_000459082.1**	**Taxonomic ID**	**Genome Assembly Accession**
100.0%	59.279867046607535%	61.29032258064516%	82.22735776383844%	42.10077580140158%	58.28705765899531%	52.49382142815718%	446372	GCF_002156525.1
Other Gene, VxrA, VxrB, VxrC, VxrD, VxrE								

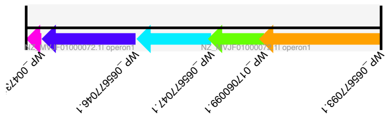

Vibrio_alginolyticus_GCF_001471275.2								
**Structural Similarity**	**Average Percent Amino Acid Identity**	**WP_001911723.1**	**WP_000815041.1**	**WP_000240569.1**	**WP_000822678.1**	**WP_000459082.1**	**Taxonomic ID**	**Genome Assembly Accession**
100.0%	58.93458389828049%	63.853211009174316%	81.8980733567901%	42.287404964807756%	50.13113736060462%	56.503092800025655%	663	GCF_001471275.2

Other Gene | VxrA | VxrB | VxrC | VxrD | VxrE

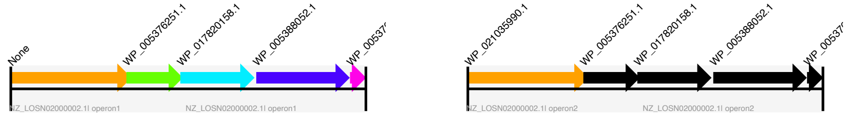

Vibrio_palustris_GCF_900162645.1								
**Structural Similarity**	**Average Percent Amino Acid Identity**	**WP_001911723.1**	**WP_000815041.1**	**WP_000240569.1**	**WP_000822678.1**	**WP_000459082.1**	**Taxonomic ID**	**Genome Assembly Accession**
100.0%	61.02729863494885%	65.03327031691761%	84.89936808190014%	49.5400628704854%	48.67697457964074%	56.98681732580037%	1918946	GCF_900162645.1

Other Gene VxrA VxrB VxrC VxrD VxrE

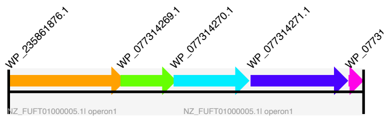

Vibrio_nereis_NBRC_15637_GCF_001591105.1

Structural Similarity	Average Percent Amino Acid Identity	WP_001911723.1	WP_000815041.1	WP_000240569.1	WP_000822678.1	WP_000459082.1	Taxonomic ID	Genome Assembly Accession
100.0%	64.1474710554596%	64.19320769402074%	87.42813952644403%	48.968385088068460%	57.95835743204164%	62.18926553672315%	1219068	GCF_001591105.1

Other Gene ■ VxrA ■ VxrB ■ VxrC ■ VxrD ■ VxrE ■

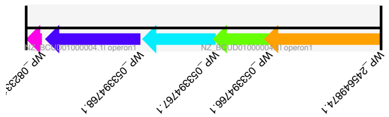

Vibrio_lentus_GCF_002874165.1								
**Structural Similarity**	**Average Percent Amino Acid Identity**	**WP_001911723.1**	**WP_000815041.1**	**WP_000240569.1**	**WP_000822678.1**	**WP_000459082.1**	**Taxonomic ID**	**Genome Assembly Accession**
100.0%	59.457341103777274%	62.04379562043796%	81.79909027548514%	44.05525142502401%	56.894746769782124%	52.49382142815718%	136468	GCF_002874165.1

Other Gene | VxrA | VxrB | VxrC | VxrD | VxrE

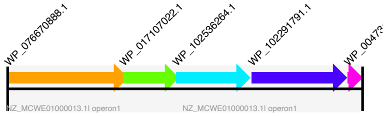

Vibrio_natriegens_NBRC_15636_ATCC_14048_DSM_759_GCF_001456255.1								
**Structural Similarity**	**Average Percent Amino Acid Identity**	**WP_001911723.1**	**WP_000815041.1**	**WP_000240569.1**	**WP_000822678.1**	**WP_000459082.1**	**Taxonomic ID**	**Genome Assembly Accession**
100.0%	57.81919366794135%	64.15441176470588%	82.89373868336631%	39.85638577482913%	50.80048475570864%	51.39094736109675%	1219067	GCF_001456255.1

Other Gene | VxrA | VxrB | VxrC | VxrD | VxrE

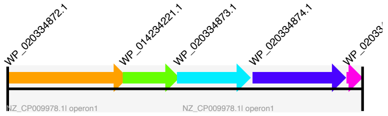

Vibrio_variabilis_GCF_003263785.1								
**Structural Similarity**	**Average Percent Amino Acid Identity**	**WP_001911723.1**	**WP_000815041.1**	**WP_000240569.1**	**WP_000822678.1**	**WP_000459082.1**	**Taxonomic ID**	**Genome Assembly Accession**
50.0%	40.81971437605822%	63.21407525318574%	81.72156396914058%	0%	0%	59.16293265796481%	990271	GCF_003263785.1

Other Gene | VxrA | VxrB | VxrC | VxrD | VxrE

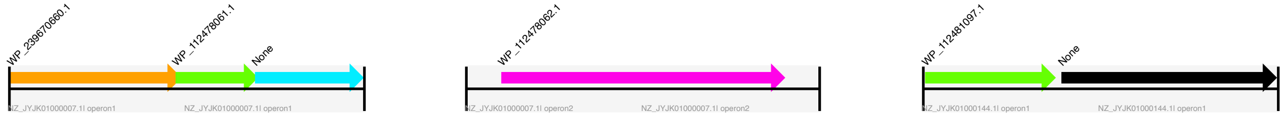

Vibrio_hangzhouensis_GCF_900107935.1								
**Structural Similarity**	**Average Percent Amino Acid Identity**	**WP_001911723.1**	**WP_000815041.1**	**WP_000240569.1**	**WP_000822678.1**	**WP_000459082.1**	**Taxonomic ID**	**Genome Assembly Accession**
100.0%	59.29883857989214%	62.734560437303664%	83.44283807303741%	45.19063697034316%	48.13647901103882%	56.98967840773764%	462991	GCF_900107935.1

Other Gene | VxrA | VxrB | VxrC | VxrD | VxrE

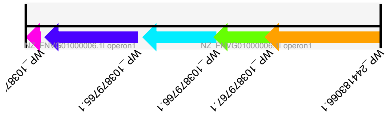

Vibrio_gallaecicus_GCF_013416885.1								
**Structural Similarity**	**Average Percent Amino Acid Identity**	**WP_001911723.1**	**WP_000815041.1**	**WP_000240569.1**	**WP_000822678.1**	**WP_000459082.1**	**Taxonomic ID**	**Genome Assembly Accession**
100.0%	59.79158701183161%	62.80384802857497%	79.83539094650206%	42.72294016629397%	56.34924753329567%	57.2465083844914%	552386	GCF_013416885.1

Other Gene | VxrA | VxrB | VxrC | VxrD | VxrE

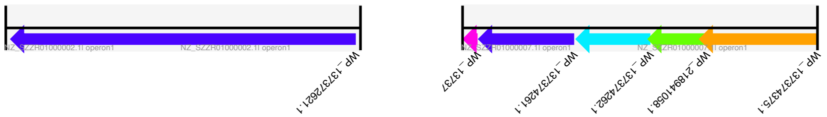

Vibrio_vulnificus_CMCP6_GCF_000039765.1								
**Structural Similarity**	**Average Percent Amino Acid Identity**	**WP_001911723.1**	**WP_000815041.1**	**WP_000240569.1**	**WP_000822678.1**	**WP_000459082.1**	**Taxonomic ID**	**Genome Assembly Accession**
100.0%	58.92144390199597%	63.33938294010889%	79.29962123414293%	42.496754895874496%	50.64377682403433%	58.827683615819204%	216895	GCF_000039765.1

Other Gene | VxrA | VxrB | VxrC | VxrD | VxrE

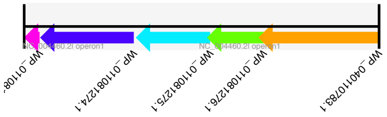

Vibrio_vulnificus_GCF_004319645.1								
**Structural Similarity**	**Average Percent Amino Acid Identity**	**WP_001911723.1**	**WP_000815041.1**	**WP_000240569.1**	**WP_000822678.1**	**WP_000459082.1**	**Taxonomic ID**	**Genome Assembly Accession**
100.0%	58.92144390199597%	63.33938294010889%	79.29962123414293%	42.4967548958744496%	50.64377682403433%	58.82768361581922%	672	GCF_004319645.1

Other Gene | VxrA | VxrB | VxrC | VxrD | VxrE

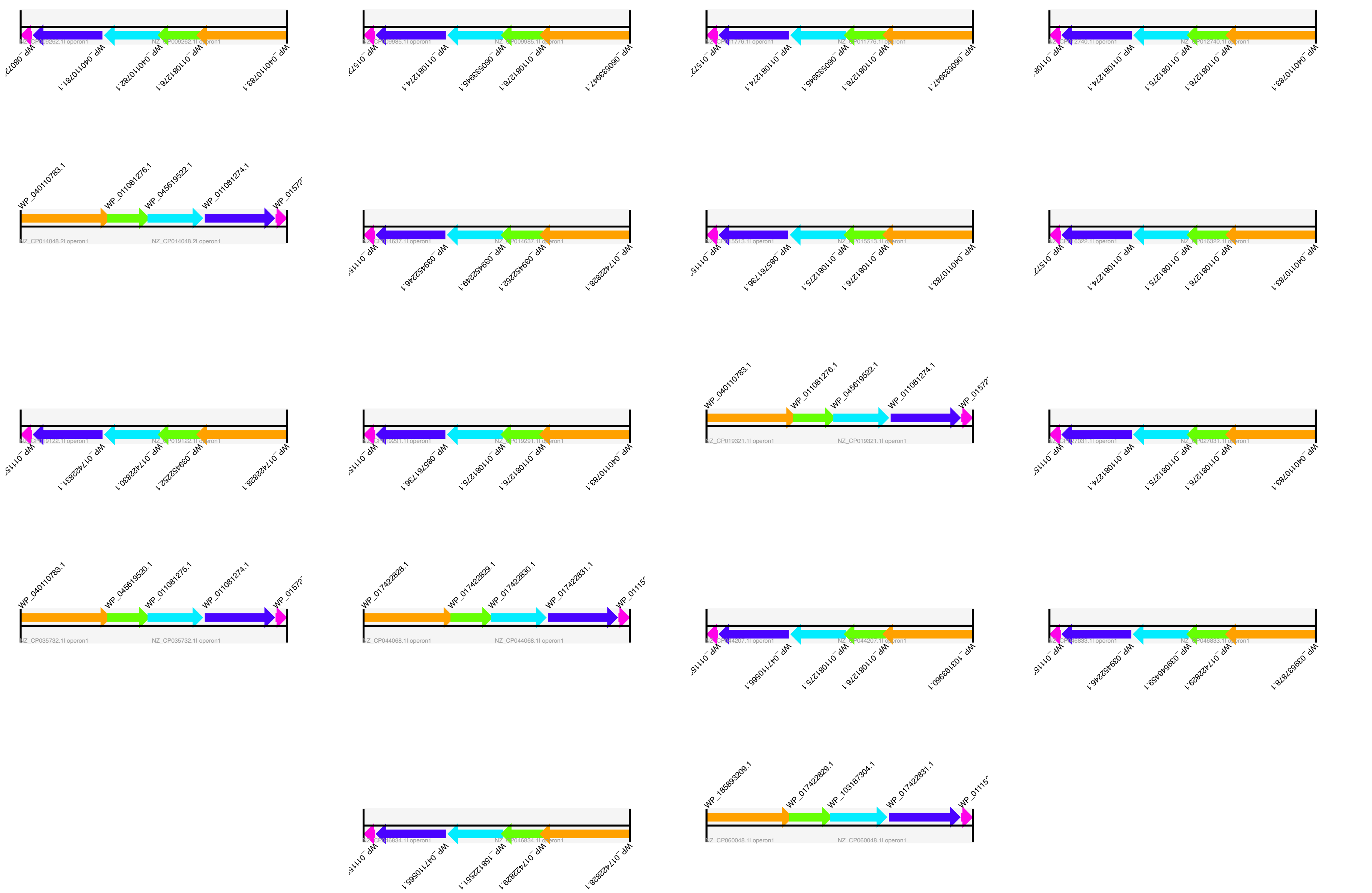

Vibrio_vulnificus_GCF_001433435.1								
Structural Similarity	Average Percent Amino Acid Identity	WP_001911723.1	WP_000815041.1	WP_000240569.1	WP_000822678.1	WP_000459082.1	Taxonomic ID	Genome Assembly Accession
100.0%	58.959654853042125%	63.33938294010889%	79.29962123414293%	42.68780965110526%	50.64377682403433%	58.827683615819204%	672	GCF_001433435.1

Other Gene | VxrA | VxrB | VxrC | VxrD | VxrE

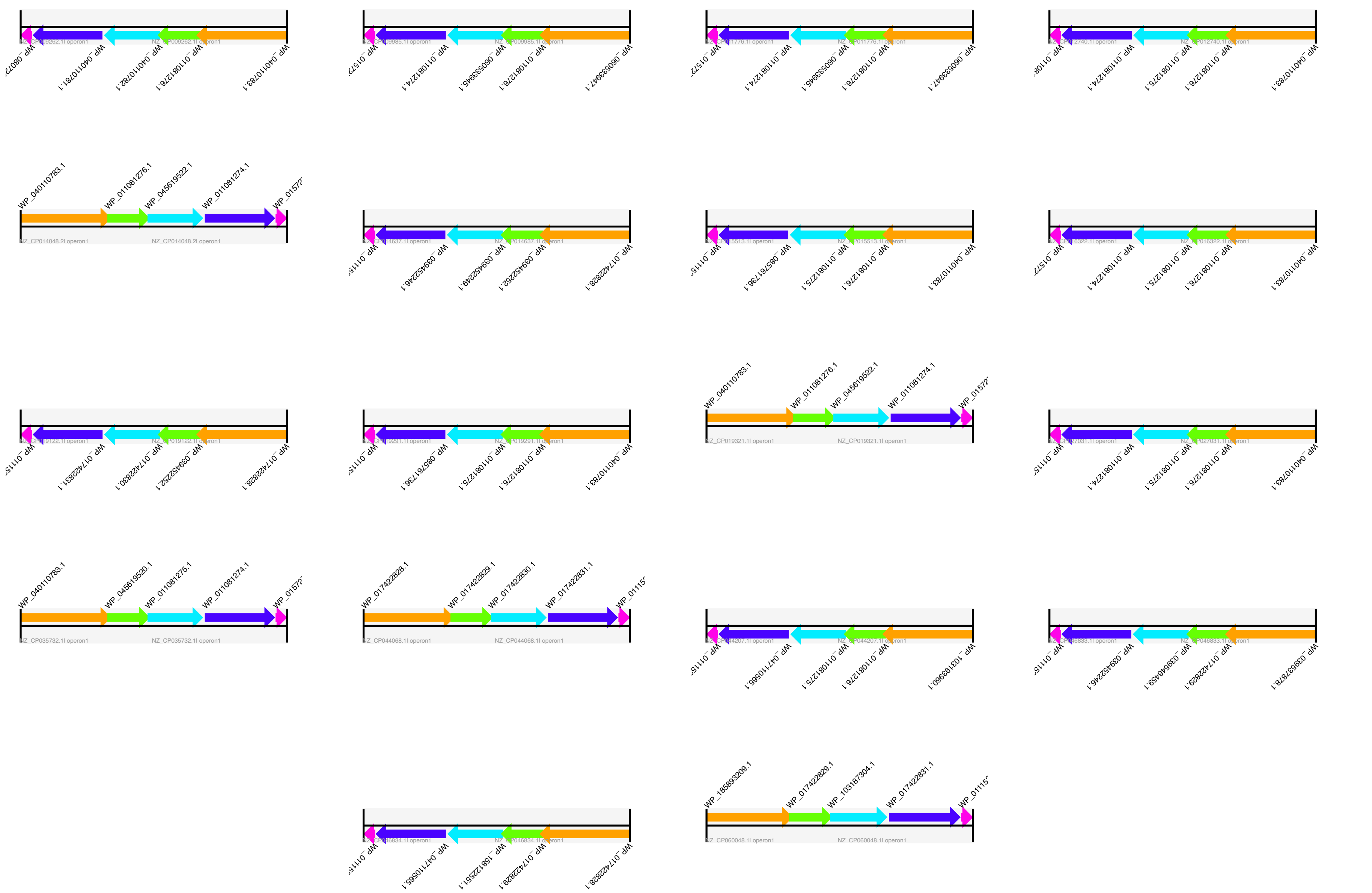

Vibrio_vulnificus_GCF_001653775.1								
Structural Similarity	Average Percent Amino Acid Identity	WP_001911723.1	WP_000815041.1	WP_000240569.1	WP_000822678.1	WP_000459082.1	Taxonomic ID	Genome Assembly Accession
100.0%	58.959654853042125%	63.33938294010889%	79.29962123414293%	42.68780965110526%	50.64377682403433%	58.827683615819204%	672	GCF_001653775.1

Other Gene VxrA VxrB VxrC VxrD VxrE

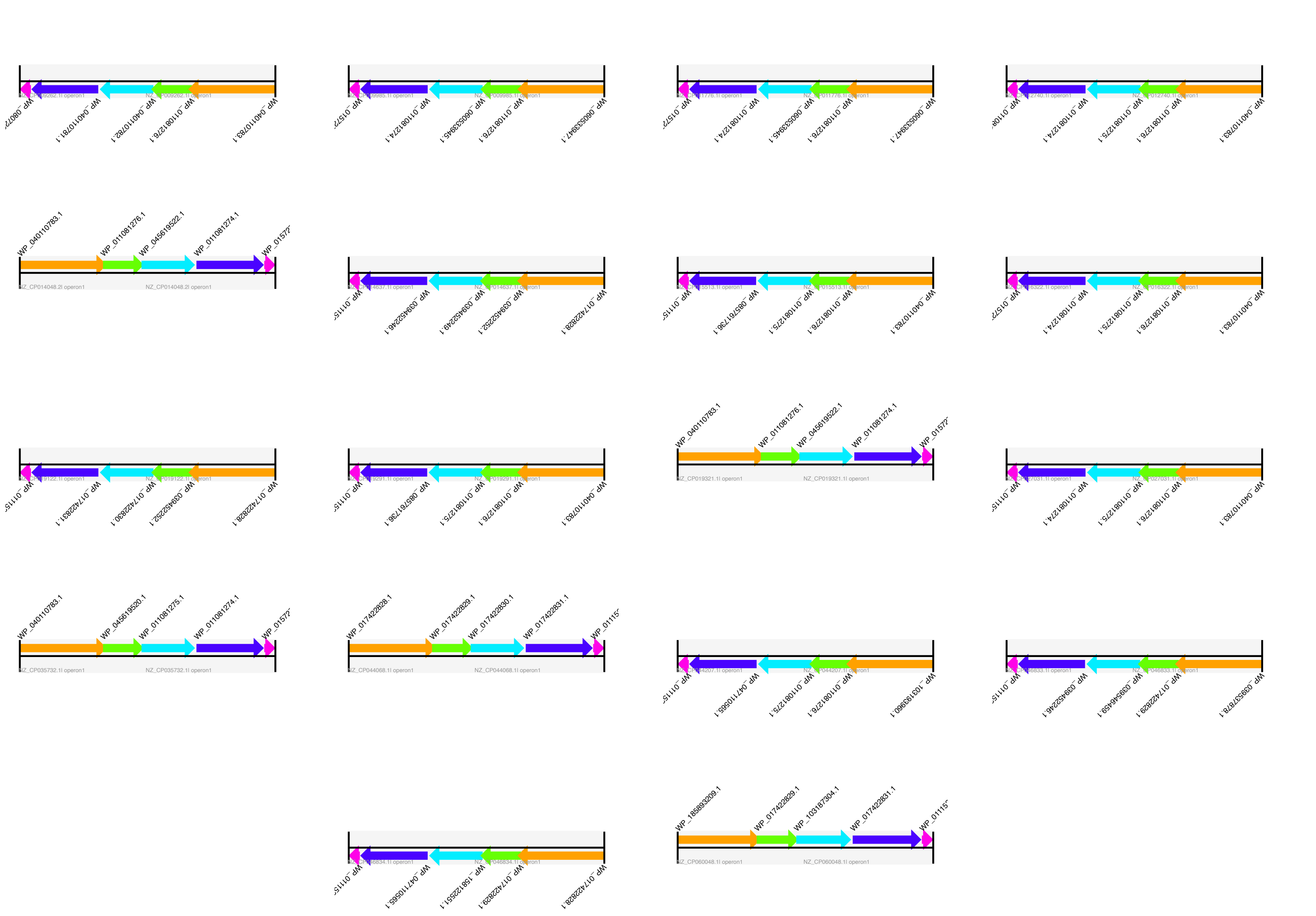

Vibrio_vulnificus_GCF_001558515.2								
**Structural Similarity**	**Average Percent Amino Acid Identity**	**WP_001911723.1**	**WP_000815041.1**	**WP_000240569.1**	**WP_000822678.1**	**WP_000459082.1**	**Taxonomic ID**	**Genome Assembly Accession**
100.0%	58.94407873842391%	63.33938294010889%	79.29962123414293%	42.60992907801418%	50.64377682403433%	58.827683615819204%	672	GCF_001558515.2

Other Gene, VxrA, VxrB, VxrC, VxrD, VxrE

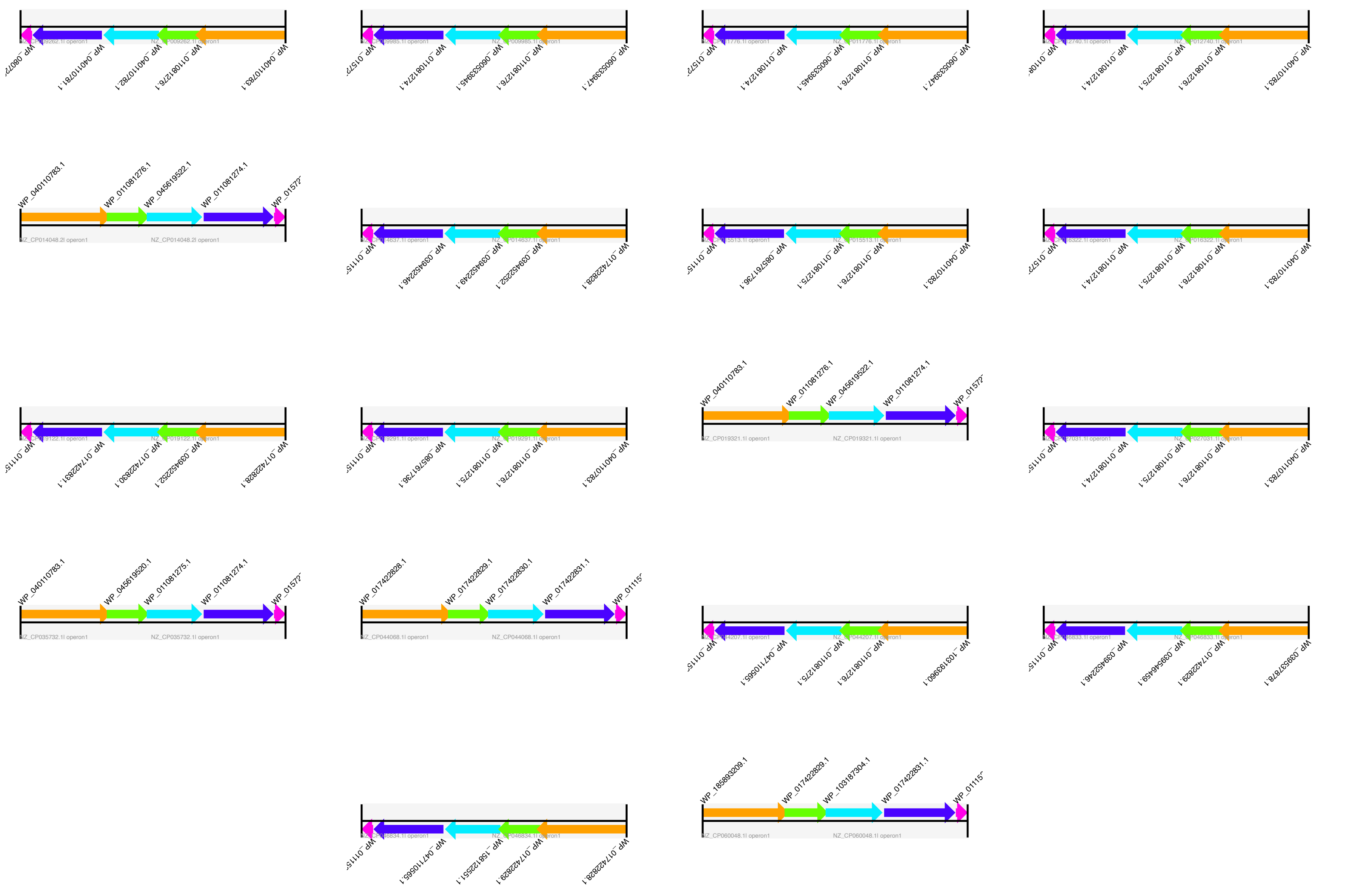

Vibrio_vulnificus_GCF_001675245.1								
Structural Similarity	Average Percent Amino Acid Identity	WP_001911723.1	WP_000815041.1	WP_000240569.1	WP_000822678.1	WP_000459082.1	Taxonomic ID	Genome Assembly Accession
100.0%	58.92144390199597%	63.33938294010889%	79.29962123414293%	42.4967548958744 96%	50.64377682403433%	58.827683615819204%	672	GCF_001675245.1

Other Gene | VxrA | VxrB | VxrC | VxrD | VxrE

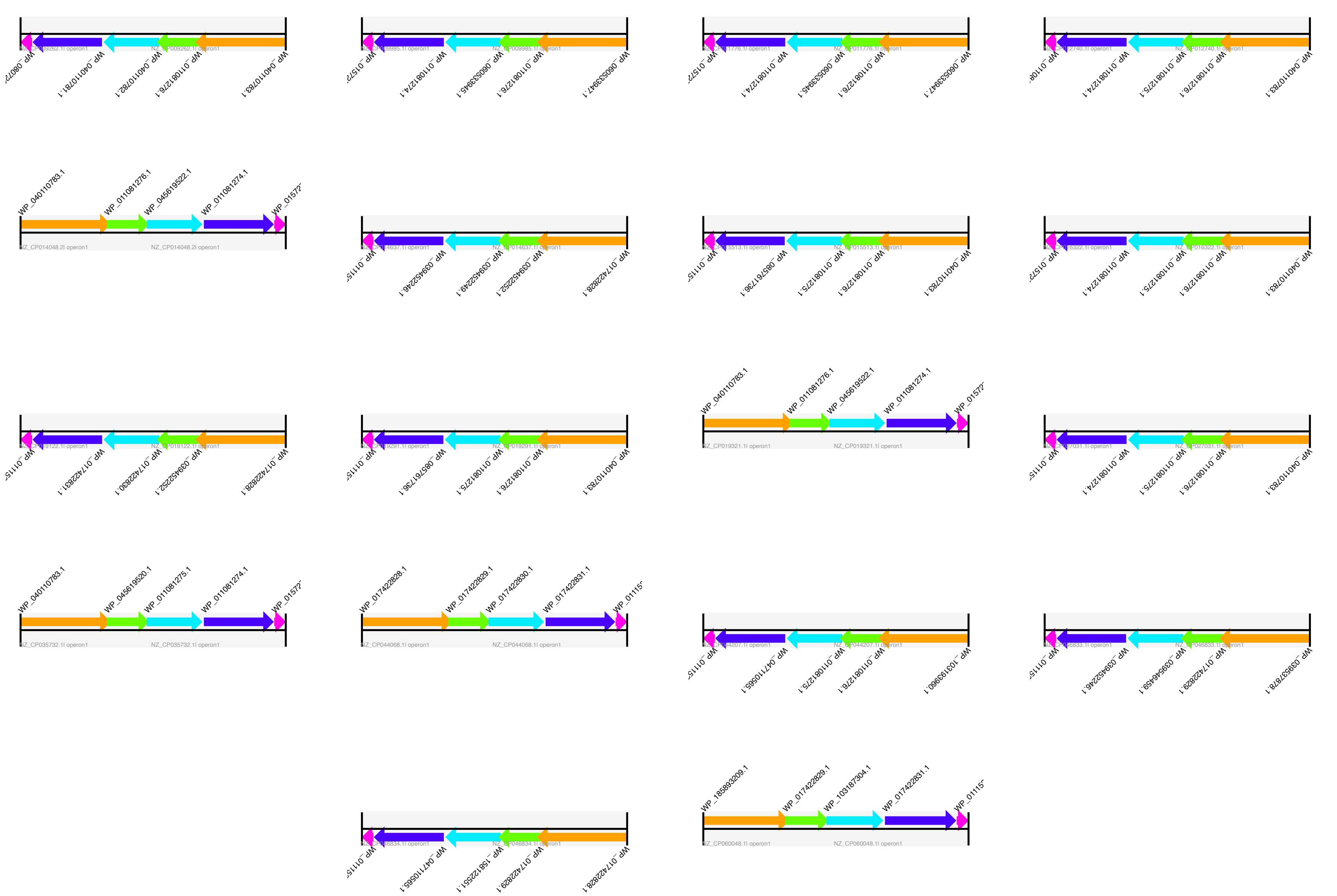

Vibrio_vulnificus_GCF_002117205.1								
**Structural Similarity**	**Average Percent Amino Acid Identity**	**WP_001911723.1**	**WP_000815041.1**	**WP_000240569.1**	**WP_000822678.1**	**WP_000459082.1**	**Taxonomic ID**	**Genome Assembly Accession**
100.0%	59.2048360426121%	63.33938294010889%	79.29962123414293%	42.496754895874496%	52.060737527114966%	58.82768361581922%	672	GCF_002117205.1

Other Gene | VxrA | VxrB | VxrC | VxrD | VxrE

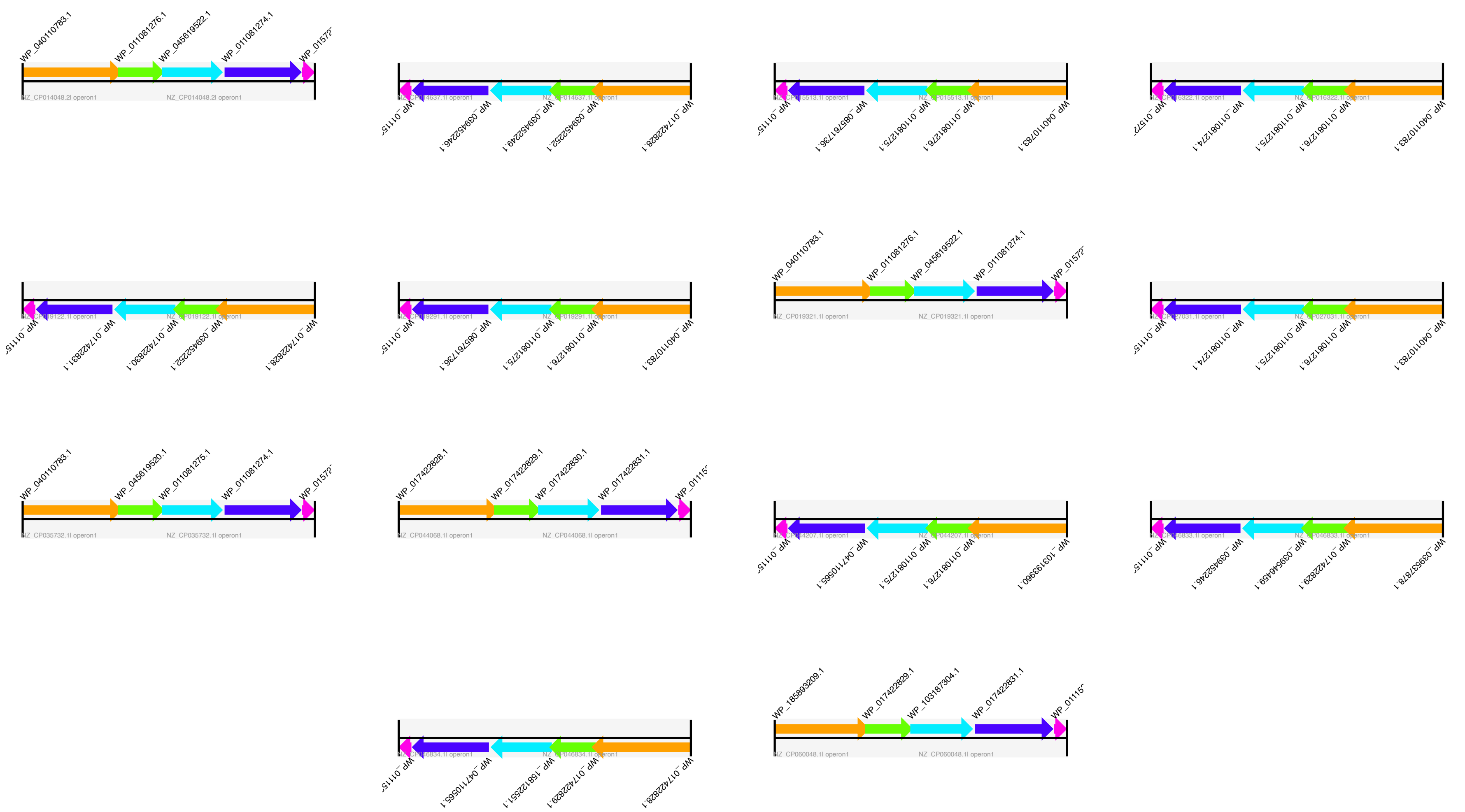

Vibrio_vulnificus_GCF_003522555.1								
**Structural Similarity**	**Average Percent Amino Acid Identity**	**WP_001911723.1**	**WP_000815041.1**	**WP_000240569.1**	**WP_000822678.1**	**WP_000459082.1**	**Taxonomic ID**	**Genome Assembly Accession**
100.0%	59.2048360426121%	63.33938294010889%	79.29962123414293%	42.496754895874496%	52.060737527114966%	58.82768361581922%	672	GCF_003522555.1

Other Gene | VxrA | VxrB | VxrC | VxrD | VxrE

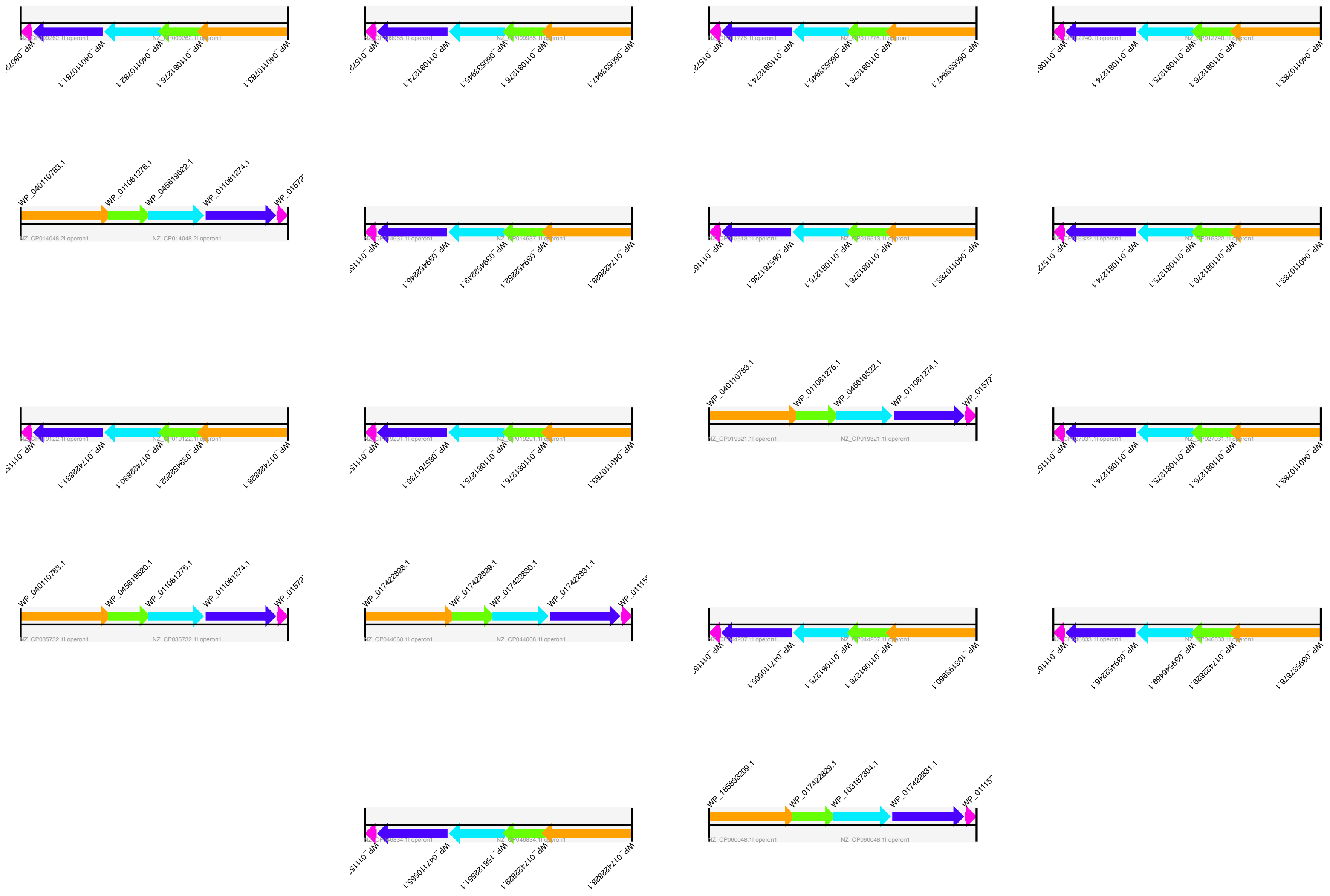

Vibrio_vulnificus_GCF_009665475.1								
Structural Similarity	Average Percent Amino Acid Identity	WP_001911723.1	WP_000815041.1	WP_000240569.1	WP_000822678.1	WP_000459082.1	Taxonomic ID	Genome Assembly Accession
100.0%	58.92144390199597%	63.33938294010889%	79.29962123414293%	42.496754895874496%	50.64377682403433%	58.827683615819204%	672	GCF_009665475.1

Other Gene | VxrA | VxrB | VxrC | VxrD | VxrE

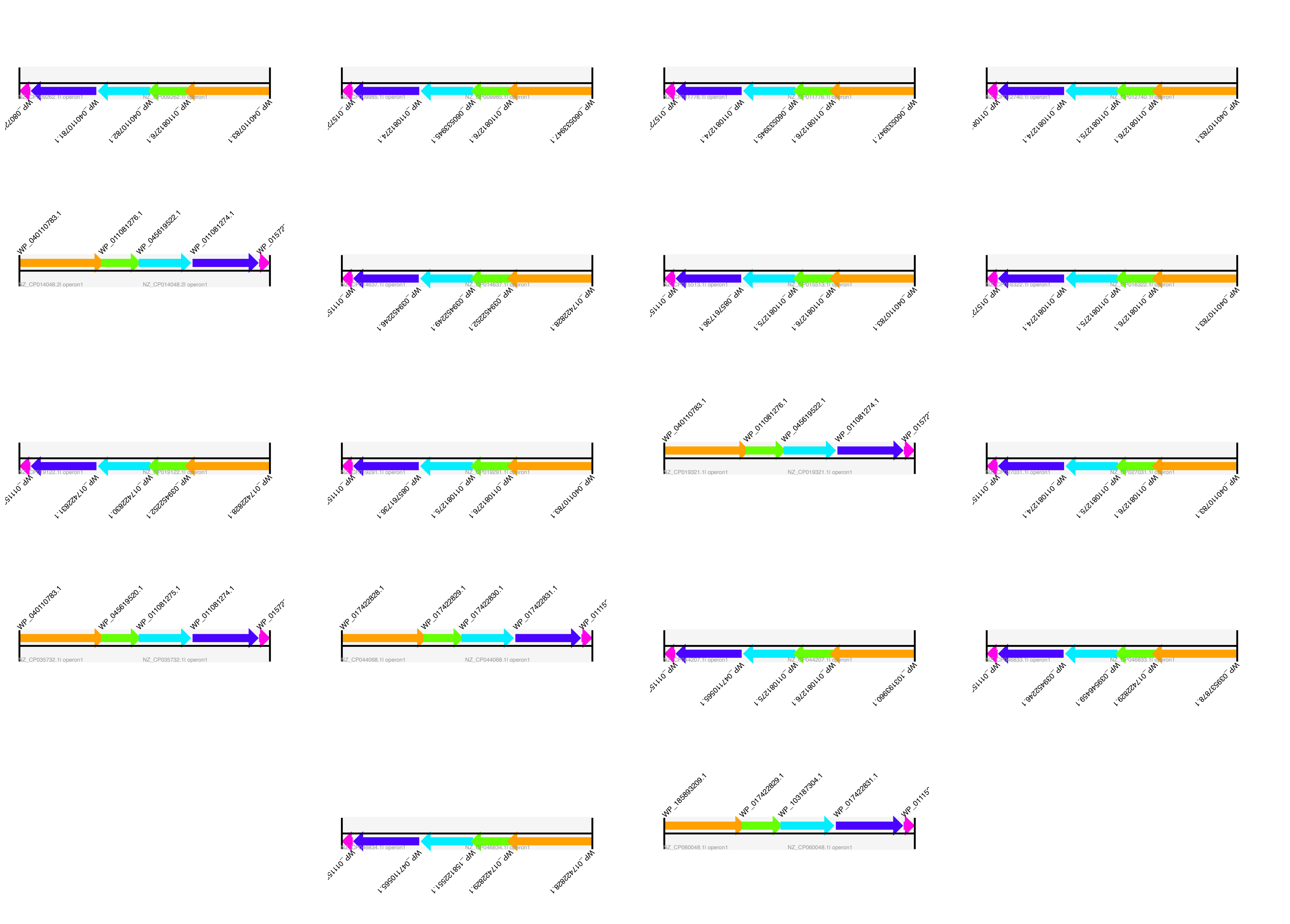

Vibrio_vulnificus_GCF_002850455.1								
**Structural Similarity**	**Average Percent Amino Acid Identity**	**WP_001911723.1**	**WP_000815041.1**	**WP_000240569.1**	**WP_000822678.1**	**WP_000459082.1**	**Taxonomic ID**	**Genome Assembly Accession**
100.0%	58.94407873842391%	63.33938294010889%	79.29962123414293%	42.60992907801418%	50.64377682403433%	58.827683615819204%	672	GCF_002850455.1

Other Gene VxrA VxrB VxrC VxrD VxrE

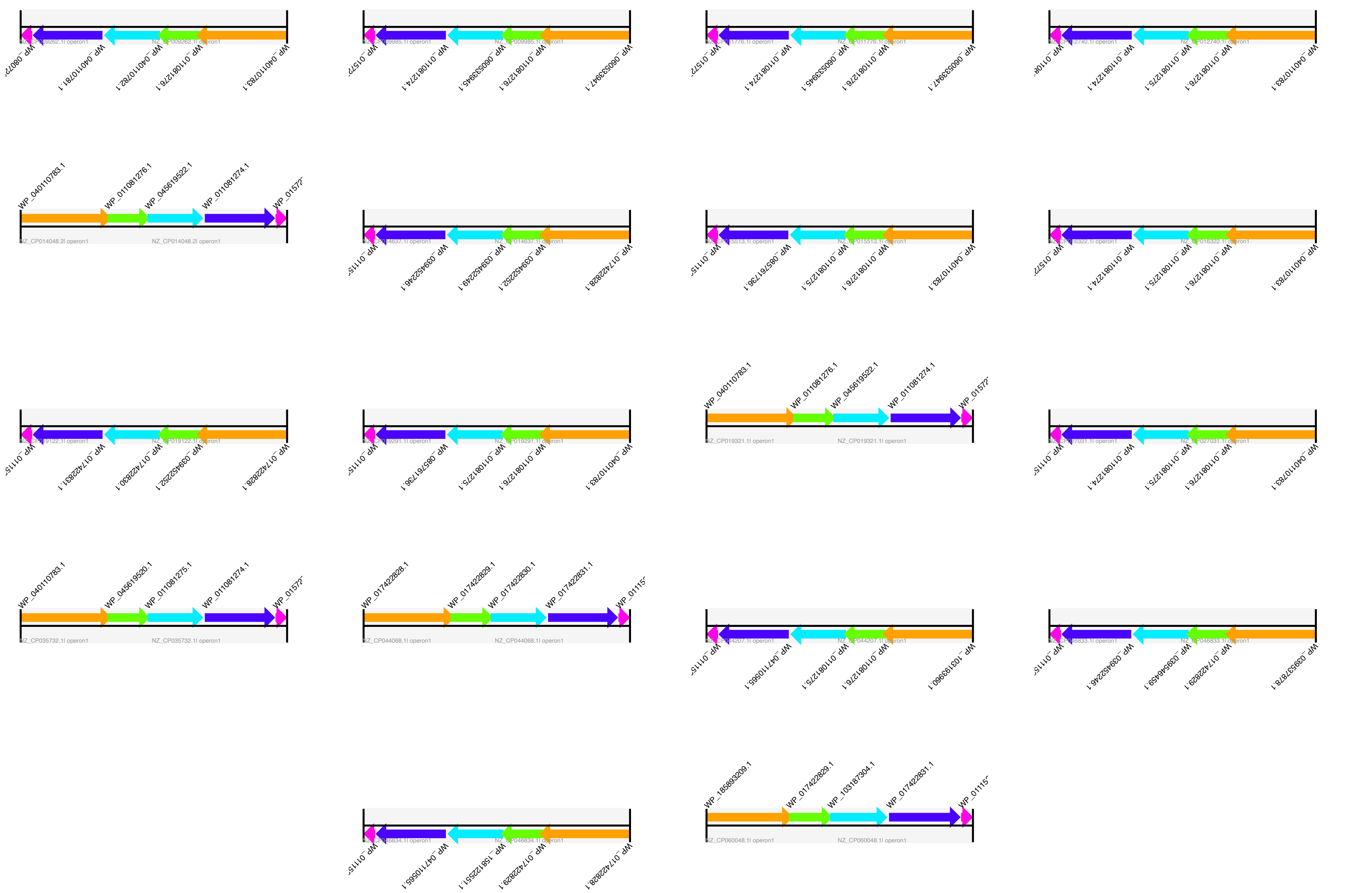

Vibrio_vulnificus_GCF_000746665.1								
Structural Similarity	Average Percent Amino Acid Identity	WP_001911723.1	WP_000815041.1	WP_000240569.1	WP_000822678.1	WP_000459082.1	Taxonomic ID	Genome Assembly Accession
100.0%	58.80672046405615%	63.33938294010889%	79.29962123414293%	42.876241134751766%	51.84381778741866%	56.6745392238584 74%	672	GCF_000746665.1

Other Gene | VxrA | VxrB | VxrC | VxrD | VxrE

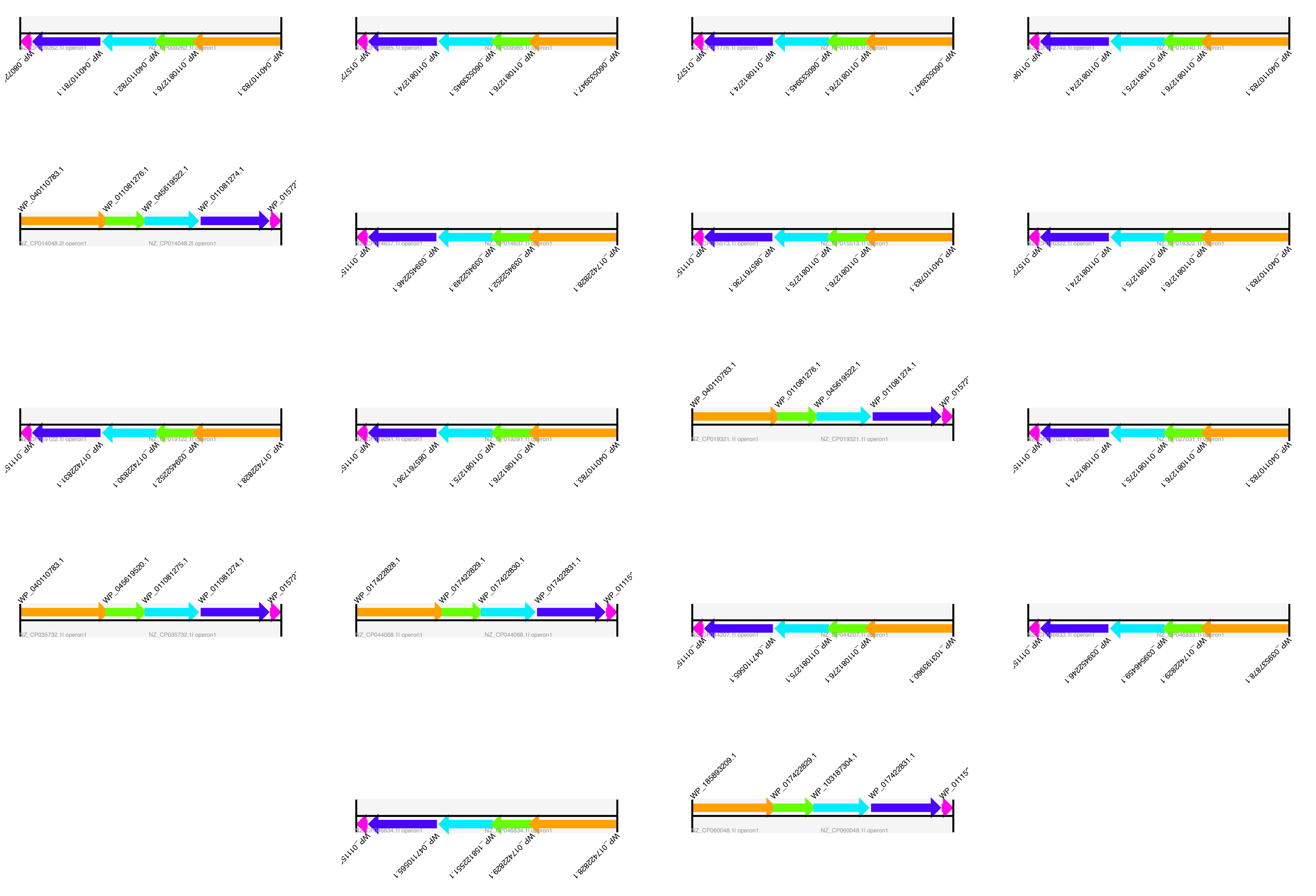

Vibrio_vulnificus_GCF_002204915.1								
Structural Similarity	Average Percent Amino Acid Identity	WP_001911723.1	WP_000815041.1	WP_000240569.1	WP_000822678.1	WP_000459082.1	Taxonomic ID	Genome Assembly Accession
100.0%	58.92144390199597%	63.33938294010889%	79.29962123414293%	42.4967548958744 96%	50.64377682403433%	58.827683615819204%	672	GCF_002204915.1

Other Gene | VxrA | VxrB | VxrC | VxrD | VxrE

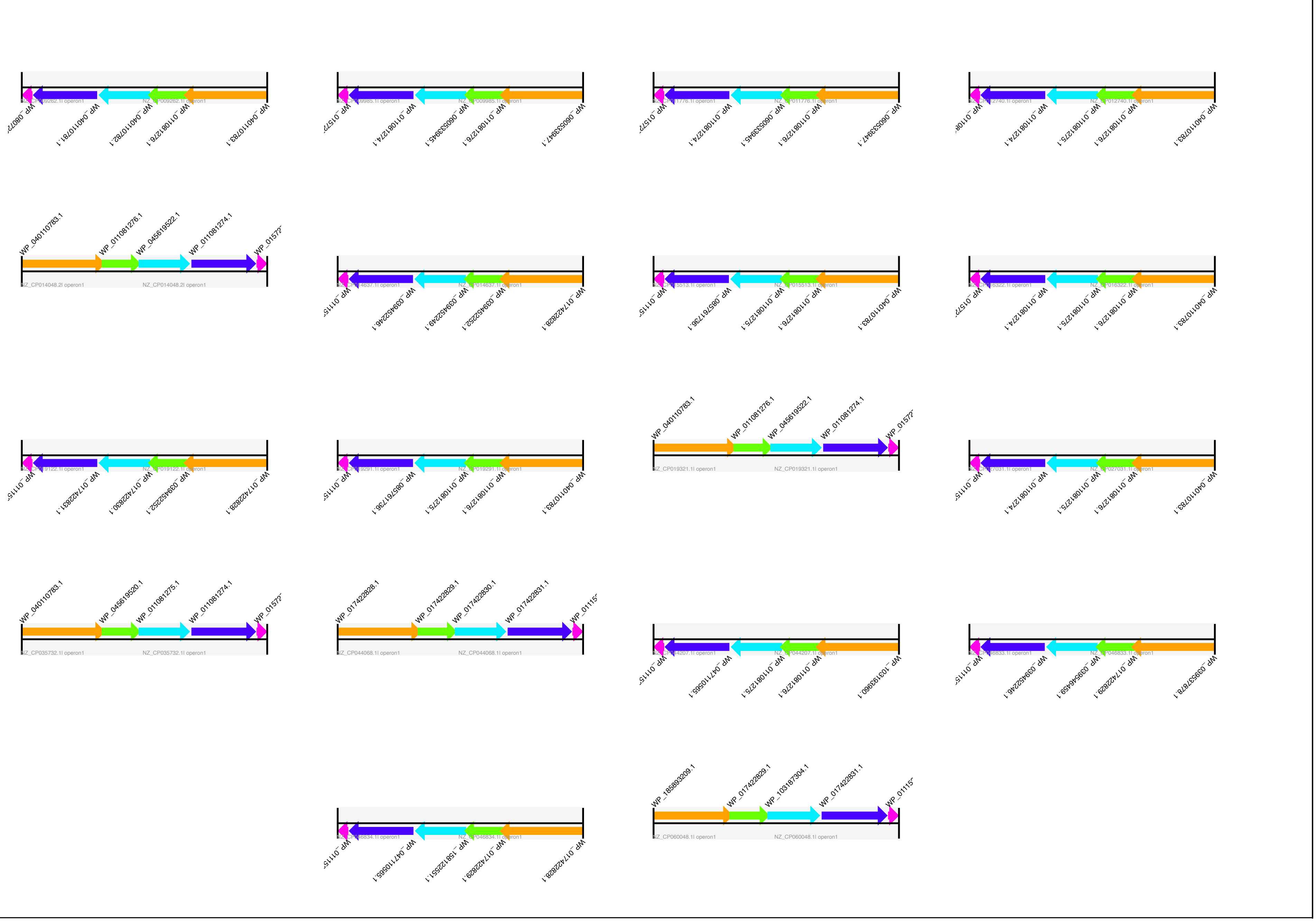

Vibrio_caribbeanicus_ATCC_BAA_2122_GCF_000165125.1								
**Structural Similarity**	**Average Percent Amino Acid Identity**	**WP_001911723.1**	**WP_000815041.1**	**WP_000240569.1**	**WP_000822678.1**	**WP_000459082.1**	**Taxonomic ID**	**Genome Assembly Accession**
100.0%	61.80535917964852%	64.57625645629098%	84.09705809239775%	48.435463807605714%	57.38595425383546%	54.53206328811267%	796620	GCF_000165125.1

Other Gene | VxrA | VxrB | VxrC | VxrD | VxrE

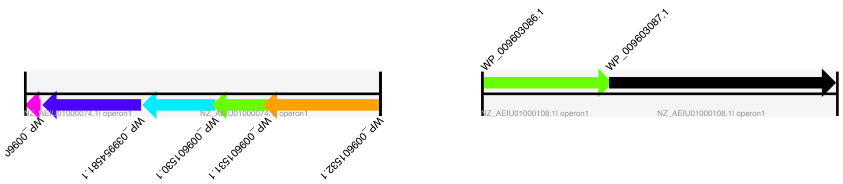

Vibrio_vulnificus_GCF_014107515.1								
**Structural Similarity**	**Average Percent Amino Acid Identity**	**WP_001911723.1**	**WP_000815041.1**	**WP_000240569.1**	**WP_000822678.1**	**WP_000459082.1**	**Taxonomic ID**	**Genome Assembly Accession**
100.0%	58.943226171597715%	63.33938294010889%	79.29962123414293%	42.496754895874496%	50.752688172043015%	58.82768361581922%	672	GCF_014107515.1

Other Gene | VxrA | VxrB | VxrC | VxrD | VxrE

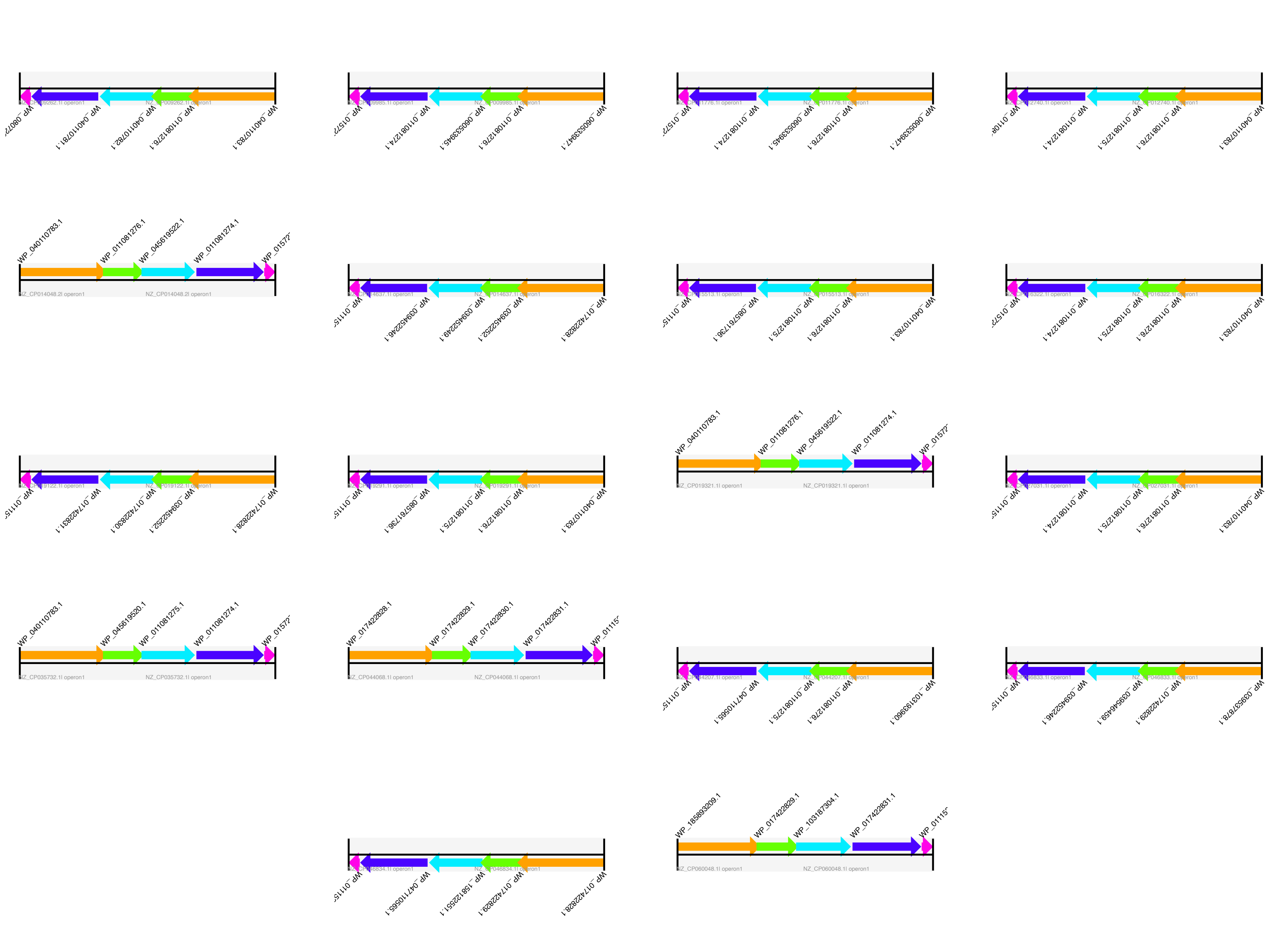

Vibrio_ichthyoenteri_ATCC_700023_GCF_000222605.1								
**Structural Similarity**	**Average Percent Amino Acid Identity**	**WP_001911723.1**	**WP_000815041.1**	**WP_000240569.1**	**WP_000822678.1**	**WP_000459082.1**	**Taxonomic ID**	**Genome Assembly Accession**
100.0%	63.44963832914768%	63.22463768115942%	85.21643965302394%	47.01017022084288%	60.79593923173597%	61.00100485897618%	870968	GCF_000222605.1

Other Gene VxrA VxrB VxrC VxrD VxrE

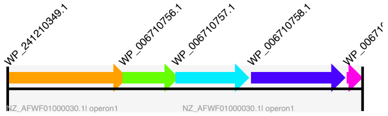

Vibrio_sinensis_GCF_003605645.1								
**Structural Similarity**	**Average Percent Amino Acid Identity**	**WP_001911723.1**	**WP_000815041.1**	**WP_000240569.1**	**WP_000822678.1**	**WP_000459082.1**	**Taxonomic ID**	**Genome Assembly Accession**
100.0%	61.24229577814327%	63.20582877959927%	86.19129582719573%	50.823347679764055%	57.665310232162646%	48.325696371994596%	2302434	GCF_003605645.1

Other Gene | VxrA | VxrB | VxrC | VxrD | VxrE

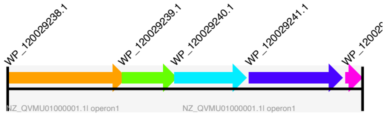

Vibrio_vulnificus_YJ016_GCF_000009745.1								
**Structural Similarity**	**Average Percent Amino Acid Identity**	**WP_001911723.1**	**WP_000815041.1**	**WP_000240569.1**	**WP_000822678.1**	**WP_000459082.1**	**Taxonomic ID**	**Genome Assembly Accession**
100.0%	58.84585233874602%	63.1578947368421%	78.87781473821667%	42.60992907801418%	50.75593952483801%	58.82768361581922%	196600	GCF_000009745.1

Other Gene, VxrA, VxrB, VxrC, VxrD, VxrE

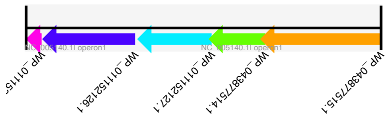

Vibrio_vulnificus_MO6_24_O_GCF_000186585.1								
**Structural Similarity**	**Average Percent Amino Acid Identity**	**WP_001911723.1**	**WP_000815041.1**	**WP_000240569.1**	**WP_000822678.1**	**WP_000459082.1**	**Taxonomic ID**	**Genome Assembly Accession**
100.0%	58.88514626134262%	63.1578947368421%	79.29962123414293%	42.496754895874496%	50.64377682403433%	58.827683615819204%	914127	GCF_000186585.1

Other Gene | VxrA | VxrB | VxrC | VxrD | VxrE

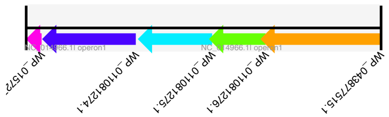

Vibrio_navarrensis_GCF_012275065.1								
**Structural Similarity**	**Average Percent Amino Acid Identity**	**WP_001911723.1**	**WP_000815041.1**	**WP_000240569.1**	**WP_000822678.1**	**WP_000459082.1**	**Taxonomic ID**	**Genome Assembly Accession**
100.0%	59.738278446050444%	62.35404853854798%	83.4814172756695%	40.56168831168831%	52.12765957446809%	60.16657852987836%	29495	GCF_012275065.1

Other Gene | VxrA | VxrB | VxrC | VxrD | VxrE

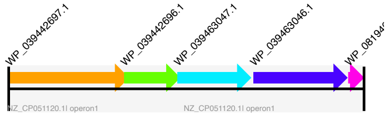

**Vibrio_fortis_GCF_000695685.1**								
**Structural Similarity**	**Average Percent Amino Acid Identity**	**WP_001911723.1**	**WP_000815041.1**	**WP_000240569.1**	**WP_000822678.1**	**WP_000459082.1**	**Taxonomic ID**	**Genome Assembly Accession**
100.0%	59.90306334471917%	62.0253164556962%	81.3708227871318%	40.92100014505367%	56.728624398725344%	58.469552936988855%	212667	GCF_000695685.1
Other Gene VxrA VxrB VxrC VxrD VxrE								

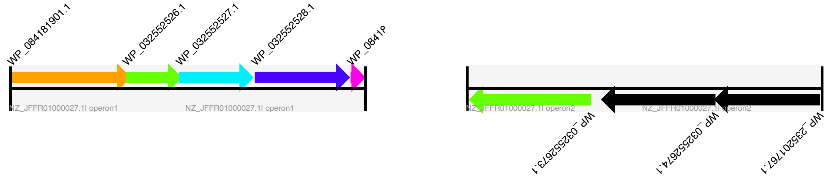

Vibrio_crassostreae_GCF_003751935.1								
**Structural Similarity**	**Average Percent Amino Acid Identity**	**WP_001911723.1**	**WP_000815041.1**	**WP_000240569.1**	**WP_000822678.1**	**WP_000459082.1**	**Taxonomic ID**	**Genome Assembly Accession**
100.0%	59.07211433791351%	61.8018018018018%	82.3283510062036%	42.6151881324295%	56.12140932097548%	52.49382142815718%	246167	GCF_003751935.1

Other Gene | VxrA | VxrB | VxrC | VxrD | VxrE

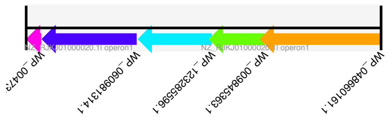

Vibrio_vulnificus_GCF_008693685.1								
Structural Similarity	Average Percent Amino Acid Identity	WP_001911723.1	WP_000815041.1	WP_000240569.1	WP_000822678.1	WP_000459082.1	Taxonomic ID	Genome Assembly Accession
100.0%	59.517984704053035%	62.794918330308526%	80.7379217151359%	42.83809551117546%	52.391304347826086%	58.82768361581922%	672	GCF_008693685.1

Other Gene | VxrA | VxrB | VxrC | VxrD | VxrE

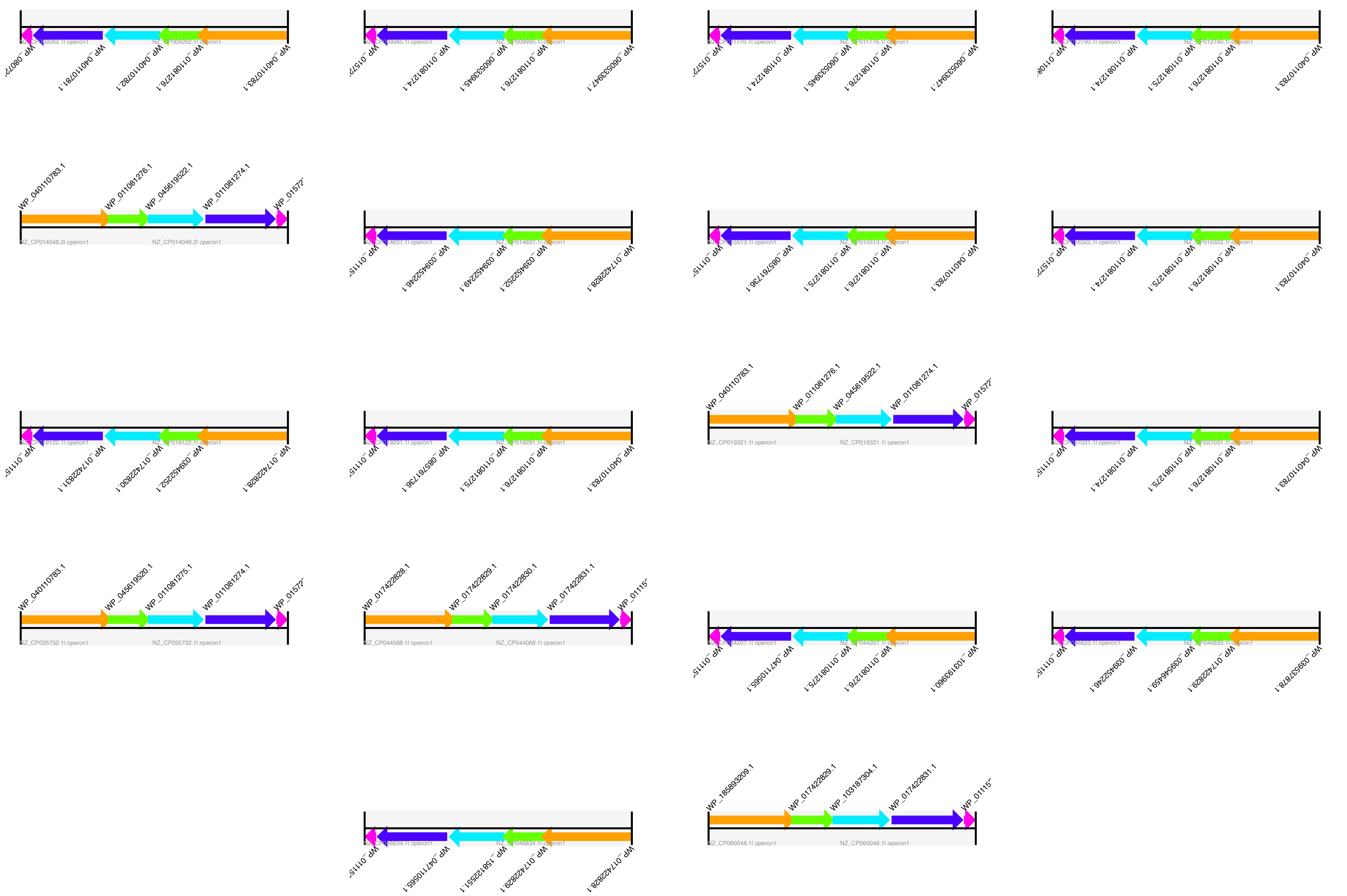

Vibrio_profundi_GCF_005281835.1								
**Structural Similarity**	**Average Percent Amino Acid Identity**	**WP_001911723.1**	**WP_000815041.1**	**WP_000240569.1**	**WP_000822678.1**	**WP_000459082.1**	**Taxonomic ID**	**Genome Assembly Accession**
100.0%	59.63897322783212%	61.950677085713416%	84.63221159001206%	42.799966229078365%	53.33807091568583%	55.47394031867091%	1774960	GCF_005281835.1

Other Gene VxrA VxrB VxrC VxrD VxrE

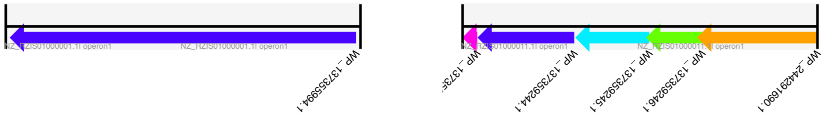

Vibrio_vulnificus_Env1_GCF_003047125.1

Structural Similarity	Average Percent Amino Acid Identity	WP_001911723.1	WP_000815041.1	WP_000240569.1	WP_000822678.1	WP_000459082.1	Taxonomic ID	Genome Assembly Accession
100.0%	59.517984704053035%	62.794918330308526%	80.7379217151359%	42.83809551117546%	52.391304347826086%	58.82768361581922%	1246305	GCF_003047125.1

Other Gene | VxrA | VxrB | VxrC | VxrD | VxrE

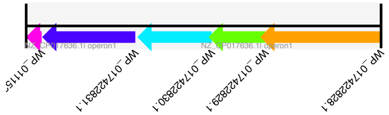

Vibrio_vulnificus_GCF_009764095.1								
**Structural Similarity**	**Average Percent Amino Acid Identity**	**WP_001911723.1**	**WP_000815041.1**	**WP_000240569.1**	**WP_000822678.1**	**WP_000459082.1**	**Taxonomic ID**	**Genome Assembly Accession**
100.0%	59.400337212916305%	62.794918330308526%	80.7379217151359%	42.684640664187484%	51.95652173913044%	58.82768361581922%	672	GCF_009764095.1

Other Gene | VxrA | VxrB | VxrC | VxrD | VxrE

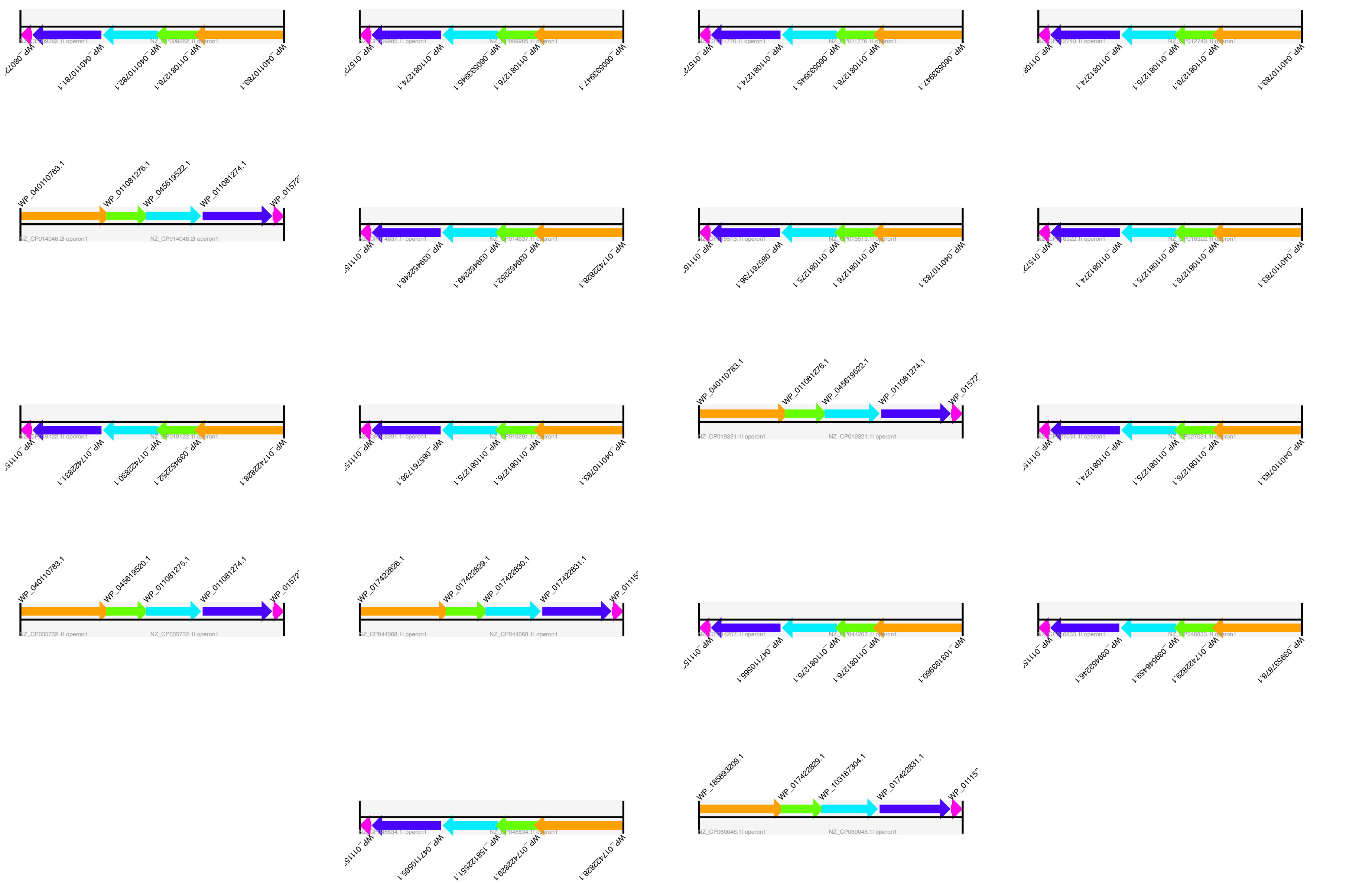

Vibrio_vulnificus_GCF_002863725.1

Structural Similarity	Average Percent Amino Acid Identity	WP_001911723.1	WP_000815041.1	WP_000240569.1	WP_000822678.1	WP_000459082.1	Taxonomic ID	Genome Assembly Accession
100.0%	59.517984704053035%	62.794918330308526%	80.7379217151359%	42.83809551117546%	52.391304347826086%	58.82768361581922%	672	GCF_002863725.1

Other Gene VxrA VxrB VxrC VxrD VxrE

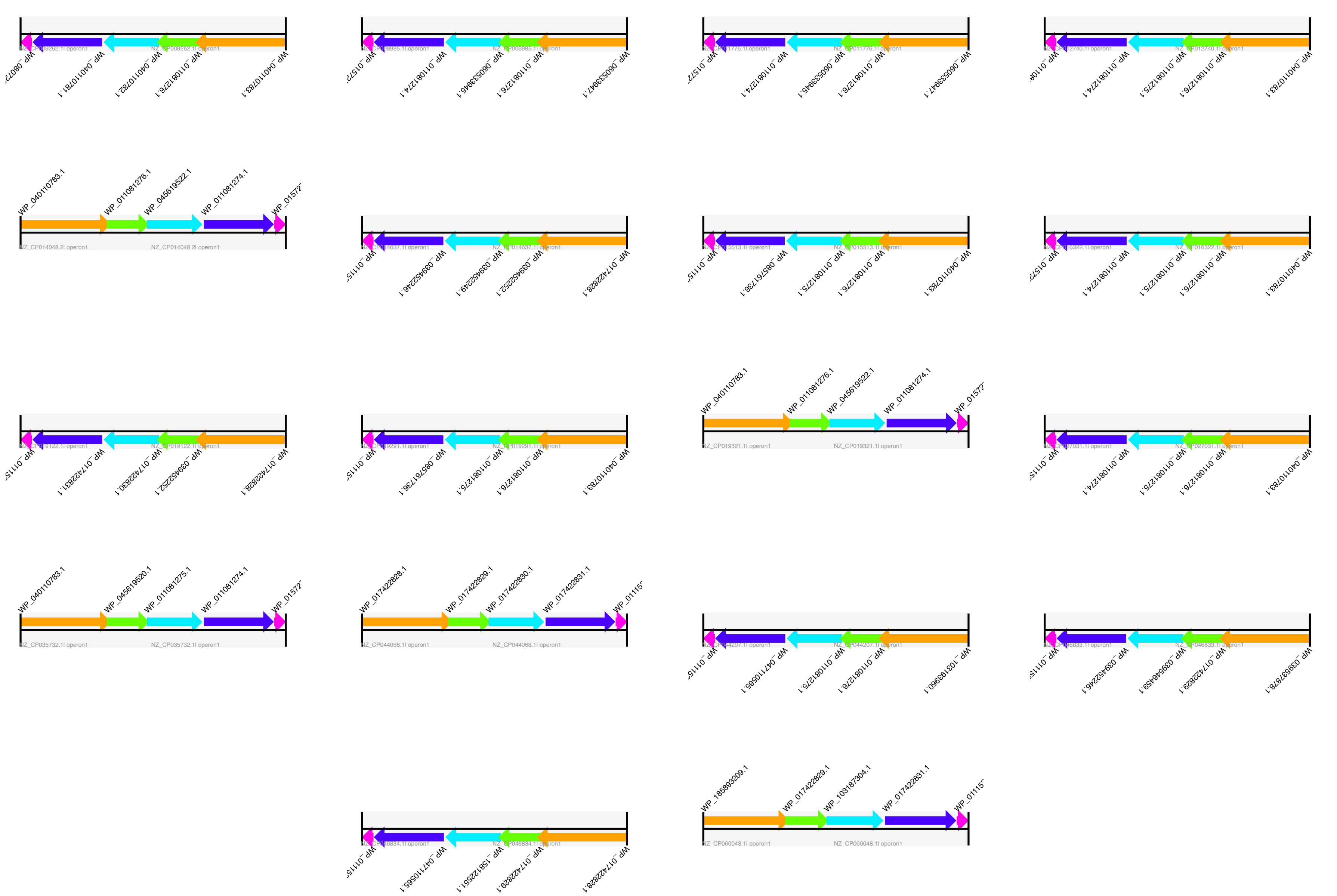

Vibrio_vulnificus_GCF_002215135.1								
Structural Similarity	Average Percent Amino Acid Identity	WP_001911723.1	WP_000815041.1	WP_000240569.1	WP_000822678.1	WP_000459082.1	Taxonomic ID	Genome Assembly Accession
100.0%	59.48457580170289%	62.794918330308526%	80.7379217151359%	43.105833608120314%	51.95652173913044%	58.82768361581922%	672	GCF_002215135.1

Other Gene VxrA VxrB VxrC VxrD VxrE

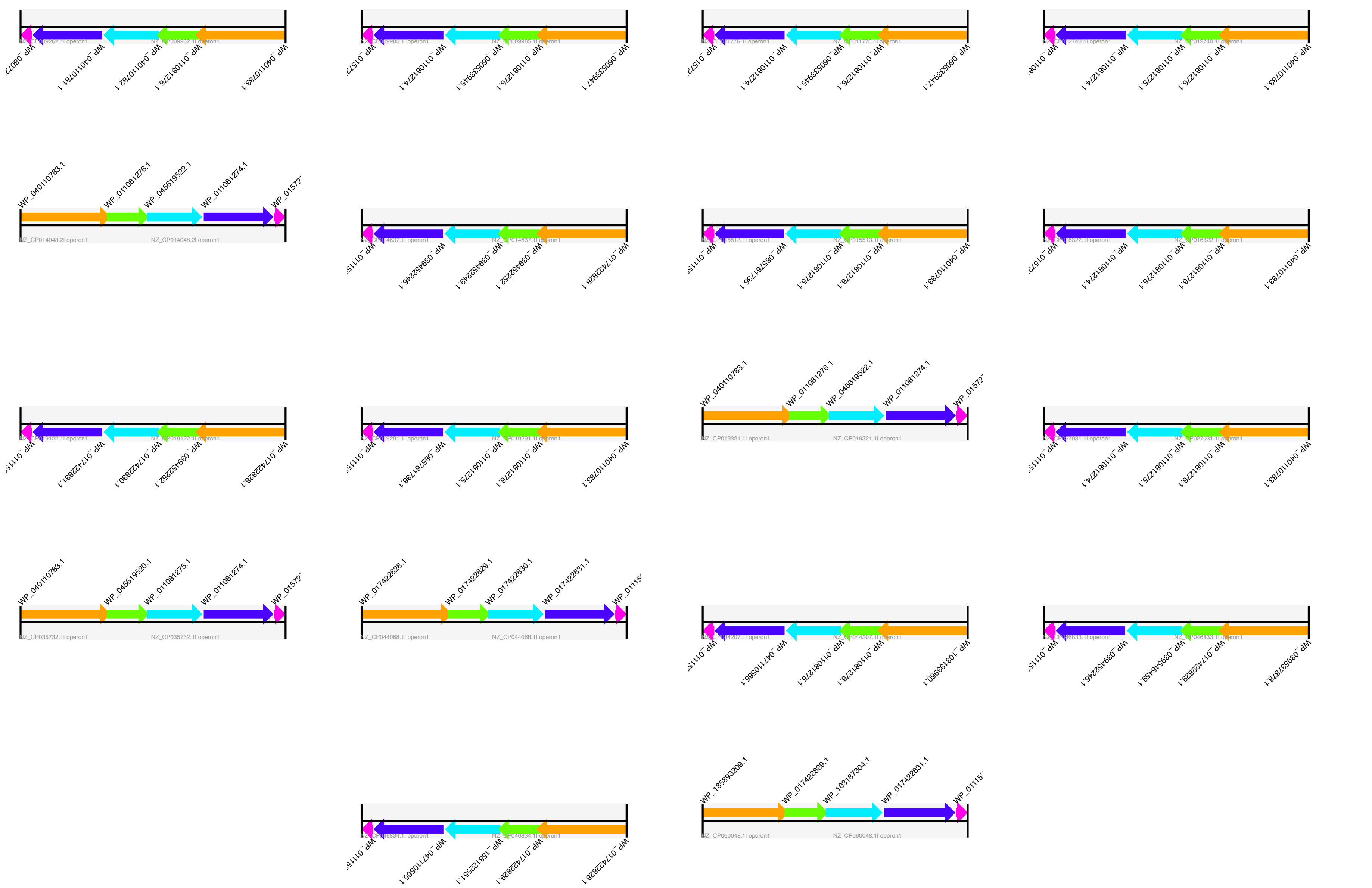

Vibrio_vulnificus_GCF_009764115.1

Structural Similarity	Average Percent Amino Acid Identity	WP_001911723.1	WP_000815041.1	WP_000240569.1	WP_000822678.1	WP_000459082.1	Taxonomic ID	Genome Assembly Accession
100.0%	59.136713849507466%	62.794918330308526%	80.7379217151359%	42.57035741423062%	50.752688172043015%	58.82768361581922%	672	GCF_009764115.1

Other Gene VxrA VxrB VxrC VxrD VxrE

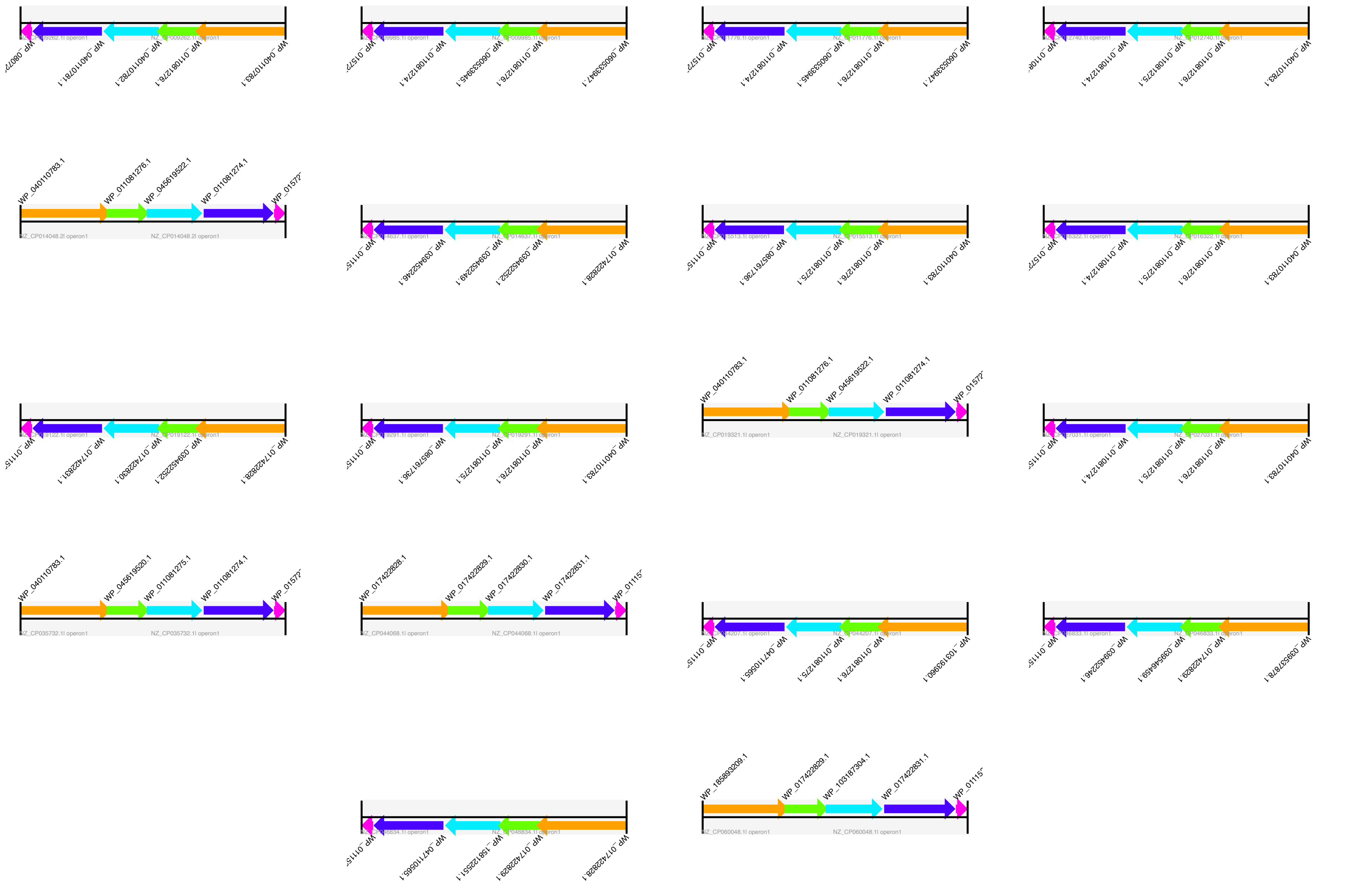

Vibrio_vulnificus_NBRC_15645_ATCC_27562_GCF_002224265.1								
**Structural Similarity**	**Average Percent Amino Acid Identity**	**WP_001911723.1**	**WP_000815041.1**	**WP_000240569.1**	**WP_000822678.1**	**WP_000459082.1**	**Taxonomic ID**	**Genome Assembly Accession**
100.0%	59.517984704053035%	62.794918330308526%	80.7379217151359%	42.83809551117546%	52.391304347826086%	58.82768361581922%	1219061	GCF_002224265.1
Other Gene	VxrA	VxrB	VxrC	VxrD	VxrE			

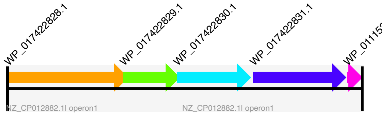

Vibrio_mediterranei_GCF_002214345.1								
**Structural Similarity**	**Average Percent Amino Acid Identity**	**WP_001911723.1**	**WP_000815041.1**	**WP_000240569.1**	**WP_000822678.1**	**WP_000459082.1**	**Taxonomic ID**	**Genome Assembly Accession**
100.0%	58.101246120387394%	60.14362657091562%	84.41637541618802%	44.553284408540975%	46.53812342274138%	54.854820783551006%	689	GCF_002214345.1

Other Gene VxrA VxrB VxrC VxrD VxrE

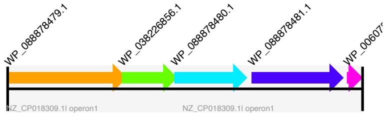

Vibrio_cidicii_GCF_001597945.1								
**Structural Similarity**	**Average Percent Amino Acid Identity**	**WP_001911723.1**	**WP_000815041.1**	**WP_000240569.1**	**WP_000822678.1**	**WP_000459082.1**	**Taxonomic ID**	**Genome Assembly Accession**
100.0%	59.59382011097223%	62.13075704823642%	82.86607015423648%	40.67803524804178%	52.12765957446809%	60.16657852987836%	1763883	GCF_001597945.1

Other Gene VxrA VxrB VxrC VxrD VxrE

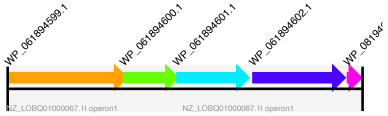

Vibrio_barjaei_GCF_001639065.2								
**Structural Similarity**	**Average Percent Amino Acid Identity**	**WP_001911723.1**	**WP_000815041.1**	**WP_000240569.1**	**WP_000822678.1**	**WP_000459082.1**	**Taxonomic ID**	**Genome Assembly Accession**
100.0%	58.20210066401795%	60.251798561151084%	84.41637541618802%	44.681009086291546%	47.0193619013234%	54.64195835513571%	1676683	GCF_001639065.2

Other Gene VxrA VxrB VxrC VxrD VxrE

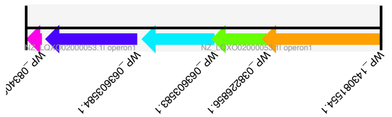

Vibrio_splendidus_GCF_003050125.1								
**Structural Similarity**	**Average Percent Amino Acid Identity**	**WP_001911723.1**	**WP_000815041.1**	**WP_000240569.1**	**WP_000822678.1**	**WP_000459082.1**	**Taxonomic ID**	**Genome Assembly Accession**
100.0%	59.66456772856094%	61.69064748201439%	82.3283510062036%	43.3741843971631%	56.28363099358531%	54.6460247638383%	29497	GCF_003050125.1
Other Gene	VxrA	VxrB	VxrC	VxrD	VxrE			

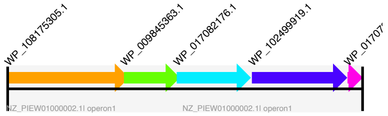

Vibrio_thalassae_GCF_900089835.1								
**Structural Similarity**	**Average Percent Amino Acid Identity**	**WP_001911723.1**	**WP_000815041.1**	**WP_000240569.1**	**WP_000822678.1**	**WP_000459082.1**	**Taxonomic ID**	**Genome Assembly Accession**
100.0%	58.17019055857993%	60.215053763440864%	84.41637541618802%	44.59286987522281%	46.64301683656824%	54.983636901479684%	1243014	GCF_900089835.1

Other Gene VxrA VxrB VxrC VxrD VxrE

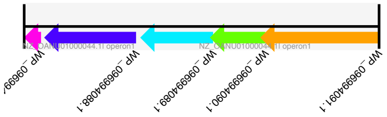

Vibrio_rhizosphaerae_DSM_18581_GCF_000711805.1								
**Structural Similarity**	**Average Percent Amino Acid Identity**	**WP_001911723.1**	**WP_000815041.1**	**WP_000240569.1**	**WP_000822678.1**	**WP_000459082.1**	**Taxonomic ID**	**Genome Assembly Accession**
75.0%	49.26656385077021%	63.00297112179399%	84.53309326805149%	44.870775744470844%	53.92597911929716%	0%	1123497	GCF_000711805.1

Other Gene | VxrA | VxrB | VxrC | VxrD | VxrE

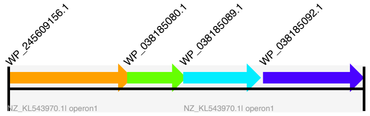

Vibrio_viridaestus_GCF_003856525.1								
**Structural Similarity**	**Average Percent Amino Acid Identity**	**WP_001911723.1**	**WP_000815041.1**	**WP_000240569.1**	**WP_000822678.1**	**WP_000459082.1**	**Taxonomic ID**	**Genome Assembly Accession**
100.0%	61.17554229914667%	61.63636363636363%	86.5946614611954%	46.45189482670061%	48.581361444431984%	62.61343012704176%	2487322	GCF_003856525.1

Other Gene | VxrA | VxrB | VxrC | VxrD | VxrE

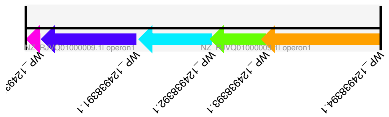

Vibrio_ruber_DSM_16370_GCF_900163965.1								
**Structural Similarity**	**Average Percent Amino Acid Identity**	**WP_001911723.1**	**WP_000815041.1**	**WP_000240569.1**	**WP_000822678.1**	**WP_000459082.1**	**Taxonomic ID**	**Genome Assembly Accession**
75.0%	48.84959514973507%	61.386188346619996%	85.71593000471962%	43.56522472820557%	53.58063266913021%	0%	1123498	GCF_900163965.1

Other Gene VxrA VxrB VxrC VxrD VxrE

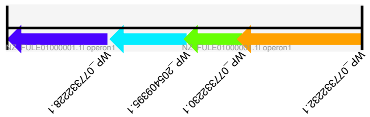

Vibrio_mytili_GCF_000830505.1								
**Structural Similarity**	**Average Percent Amino Acid Identity**	**WP_001911723.1**	**WP_000815041.1**	**WP_000240569.1**	**WP_000822678.1**	**WP_000459082.1**	**Taxonomic ID**	**Genome Assembly Accession**
75.0%	35.22510597777031%	0%	82.4638979277038%	43.3128868905159%	50.34874507063187%	0%	50718	GCF_000830505.1

Other Gene VxrA VxrB VxrC VxrD VxrE

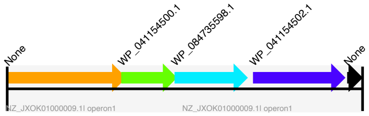

Vibrio_spartinae_GCF_014083925.1								
**Structural Similarity**	**Average Percent Amino Acid Identity**	**WP_001911723.1**	**WP_000815041.1**	**WP_000240569.1**	**WP_000822678.1**	**WP_000459082.1**	**Taxonomic ID**	**Genome Assembly Accession**
75.0%	48.61058599335204%	60.649350649350644%	84.89476081273057%	43.79768392370572%	53.71113458097327%	0%	1918945	GCF_014083925.1

Other Gene VxrA VxrB VxrC VxrD VxrE

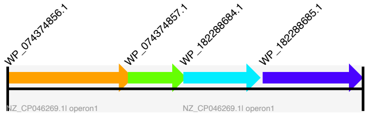

Vibrio_gazogenes_DSM_21264_NBRC_103151_GCF_900129185.1								
**Structural Similarity**	**Average Percent Amino Acid Identity**	**WP_001911723.1**	**WP_000815041.1**	**WP_000240569.1**	**WP_000822678.1**	**WP_000459082.1**	**Taxonomic ID**	**Genome Assembly Accession**
75.0%	49.316547990517805%	61.79173181084573%	84.82768202539607%	46.03734699705007%	53.92597911929716%	0%	1123492	GCF_900129185.1

Other Gene | VxrA | VxrB | VxrC | VxrD | VxrE

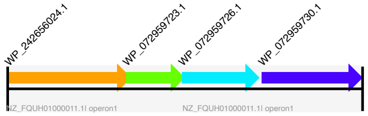

Vibrio_aerogenes_CECT_7868_GCF_900130105.1								
**Structural Similarity**	**Average Percent Amino Acid Identity**	**WP_001911723.1**	**WP_000815041.1**	**WP_000240569.1**	**WP_000822678.1**	**WP_000459082.1**	**Taxonomic ID**	**Genome Assembly Accession**
75.0%	48.09203062270896%	59.96376811594203%	86.34117817194623%	42.932929139825696%	51.222277685830875%	0%	1216006	GCF_900130105.1

Other Gene VxrA VxrB VxrC VxrD VxrE

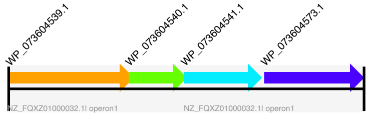

Vibrio_quintilis_GCF_900143745.1								
**Structural Similarity**	**Average Percent Amino Acid Identity**	**WP_001911723.1**	**WP_000815041.1**	**WP_000240569.1**	**WP_000822678.1**	**WP_000459082.1**	**Taxonomic ID**	**Genome Assembly Accession**
75.0%	48.63969670735928%	59.92779783393502%	87.35416970167026%	43.064804344487726%	52.851711656703436%	0%	1117707	GCF_900143745.1

Other Gene | VxrA | VxrB | VxrC | VxrD | VxrE

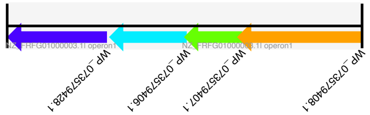

Vibrio_taketomensis_GCF_009938185.1								
**Structural Similarity**	**Average Percent Amino Acid Identity**	**WP_001911723.1**	**WP_000815041.1**	**WP_000240569.1**	**WP_000822678.1**	**WP_000459082.1**	**Taxonomic ID**	**Genome Assembly Accession**
100.0%	50.33320935453904%	0%	85.96647416225694%	48.85371493113782%	57.17421973972131%	59.671637939579135%	2572923	GCF_009938185.1

Other Gene | VxrA | VxrB | VxrC | VxrD | VxrE

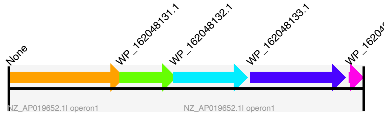

Vibrio_azureus_GCF_002849855.1								
**Structural Similarity**	**Average Percent Amino Acid Identity**	**WP_001911723.1**	**WP_000815041.1**	**WP_000240569.1**	**WP_000822678.1**	**WP_000459082.1**	**Taxonomic ID**	**Genome Assembly Accession**
50.0%	45.944220799998256%	58.67620751341681%	78.79309883835737%	35.75995615637561%	0%	56.49184149184149%	512649	GCF_002849855.1

Other Gene | VxrA | VxrB | VxrC | VxrD | VxrE

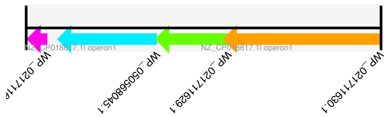

Vibrio_mangrovi_GCF_900184095.1								
**Structural Similarity**	**Average Percent Amino Acid Identity**	**WP_001911723.1**	**WP_000815041.1**	**WP_000240569.1**	**WP_000822678.1**	**WP_000459082.1**	**Taxonomic ID**	**Genome Assembly Accession**
75.0%	50.10931886280463%	61.15107913669063%	87.93654995302835%	46.730230898785344%	54.728734325518815%	0%	474394	GCF_900184095.1

Other Gene VxrA VxrB VxrC VxrD VxrE

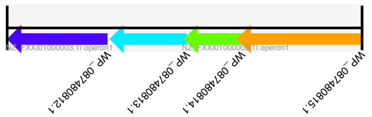

Vibrio_astriarenae_GCF_010587385.1								
**Structural Similarity**	**Average Percent Amino Acid Identity**	**WP_001911723.1**	**WP_000815041.1**	**WP_000240569.1**	**WP_000822678.1**	**WP_000459082.1**	**Taxonomic ID**	**Genome Assembly Accession**
100.0%	56.44363743925733%	59.430604982206404%	81.00341319629062%	40.12330386106497%	46.496920084711306%	55.16394507201334%	1481923	GCF_010587385.1
Other Gene: black; VxrA: orange; VxrB: green; VxrC: cyan; VxrD: blue; VxrE: magenta								

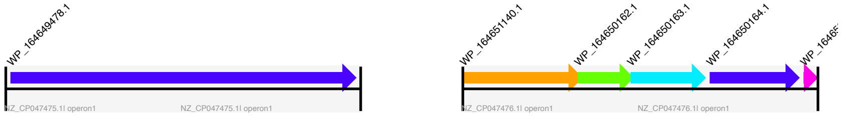

Vibrio_sagamiensis_NBRC_104589_GCF_007990935.1								
**Structural Similarity**	**Average Percent Amino Acid Identity**	**WP_001911723.1**	**WP_000815041.1**	**WP_000240569.1**	**WP_000822678.1**	**WP_000459082.1**	**Taxonomic ID**	**Genome Assembly Accession**
50.0%	47.2616533929839%	58.69565217391305%	79.77237780435001%	35.821006217425655%	0%	62.019230769230774%	1219064	GCF_007990935.1

Other Gene ■ VxrA ■ VxrB ■ VxrC ■ VxrD ■ VxrE ■

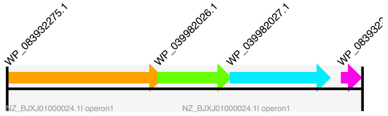

Vibrio_tapetis_subsp_tapetis_GCF_900233005.1								
**Structural Similarity**	**Average Percent Amino Acid Identity**	**WP_001911723.1**	**WP_000815041.1**	**WP_000240569.1**	**WP_000822678.1**	**WP_000459082.1**	**Taxonomic ID**	**Genome Assembly Accession**
75.0%	44.37790045485222%	59.00000383963799%	77.0219739490385%	38.27472505736213%	47.59279942822246%	0%	1671868	GCF_900233005.1

Other Gene VxrA VxrB VxrC VxrD VxrE

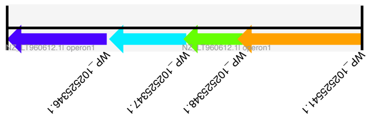

Vibrio_penaeicida_GCF_003970425.1								
**Structural Similarity**	**Average Percent Amino Acid Identity**	**WP_001911723.1**	**WP_000815041.1**	**WP_000240569.1**	**WP_000822678.1**	**WP_000459082.1**	**Taxonomic ID**	**Genome Assembly Accession**
100.0%	56.5294038235068%	57.24258289703316%	78.31898771119684%	41.48604966041993%	44.53528650724093%	61.06411234164315%	104609	GCF_003970425.1

Other Gene | VxrA | VxrB | VxrC | VxrD | VxrE

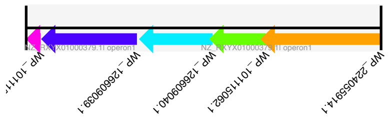

Vibrio_nigripulchritudo_POn4_GCF_001050675.1

Structural Similarity	Average Percent Amino Acid Identity	WP_001911723.1	WP_000815041.1	WP_000240569.1	WP_000822678.1	WP_000459082.1	Taxonomic ID	Genome Assembly Accession
100.0%	56.32187211026287%	57.965937465518245%	79.08965806994084%	40.7409036742127745%	44.8082193502967%	59.00464199134583%	1238445	GCF_001050675.1

Other Gene | VxrA | VxrB | VxrC | VxrD | VxrE

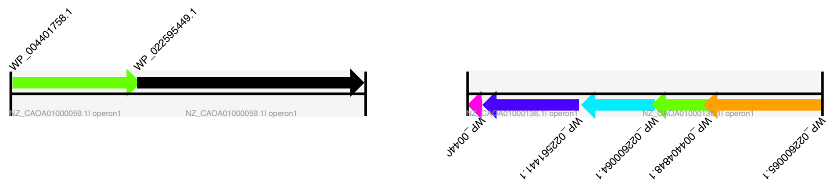

Vibrio_maerlii_GCF_003415655.1								
**Structural Similarity**	**Average Percent Amino Acid Identity**	**WP_001911723.1**	**WP_000815041.1**	**WP_000240569.1**	**WP_000822678.1**	**WP_000459082.1**	**Taxonomic ID**	**Genome Assembly Accession**
100.0%	57.585071756796914%	54.91589782213017%	82.27043337364147%	37.769824006327866%	50.42553191489362%	62.543671666991415%	2231648	GCF_003415655.1

Other Gene | VxrA | VxrB | VxrC | VxrD | VxrE

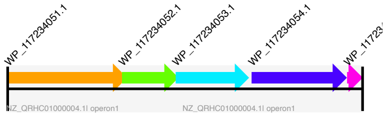

Vibrio_aphrogenes_GCF_002157735.2								
**Structural Similarity**	**Average Percent Amino Acid Identity**	**WP_001911723.1**	**WP_000815041.1**	**WP_000240569.1**	**WP_000822678.1**	**WP_000459082.1**	**Taxonomic ID**	**Genome Assembly Accession**
50.0%	47.234431488535606%	55.45774647887324%	73.12098360441793%	39.361965083006226%	0%	68.23146227638061%	1891186	GCF_002157735.2

Other Gene VxrA VxrB VxrC VxrD VxrE

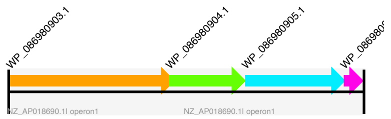

Vibrio_vulnificus_GCF_014211935.1								
**Structural Similarity**	**Average Percent Amino Acid Identity**	**WP_001911723.1**	**WP_000815041.1**	**WP_000240569.1**	**WP_000822678.1**	**WP_000459082.1**	**Taxonomic ID**	**Genome Assembly Accession**
100.0%	58.49765313428717%	57.846715328467155%	80.7379217151359%	42.684640664187484%	52.391304347826086%	58.82768361581922%	672	GCF_014211935.1

Other Gene | VxrA | VxrB | VxrC | VxrD | VxrE

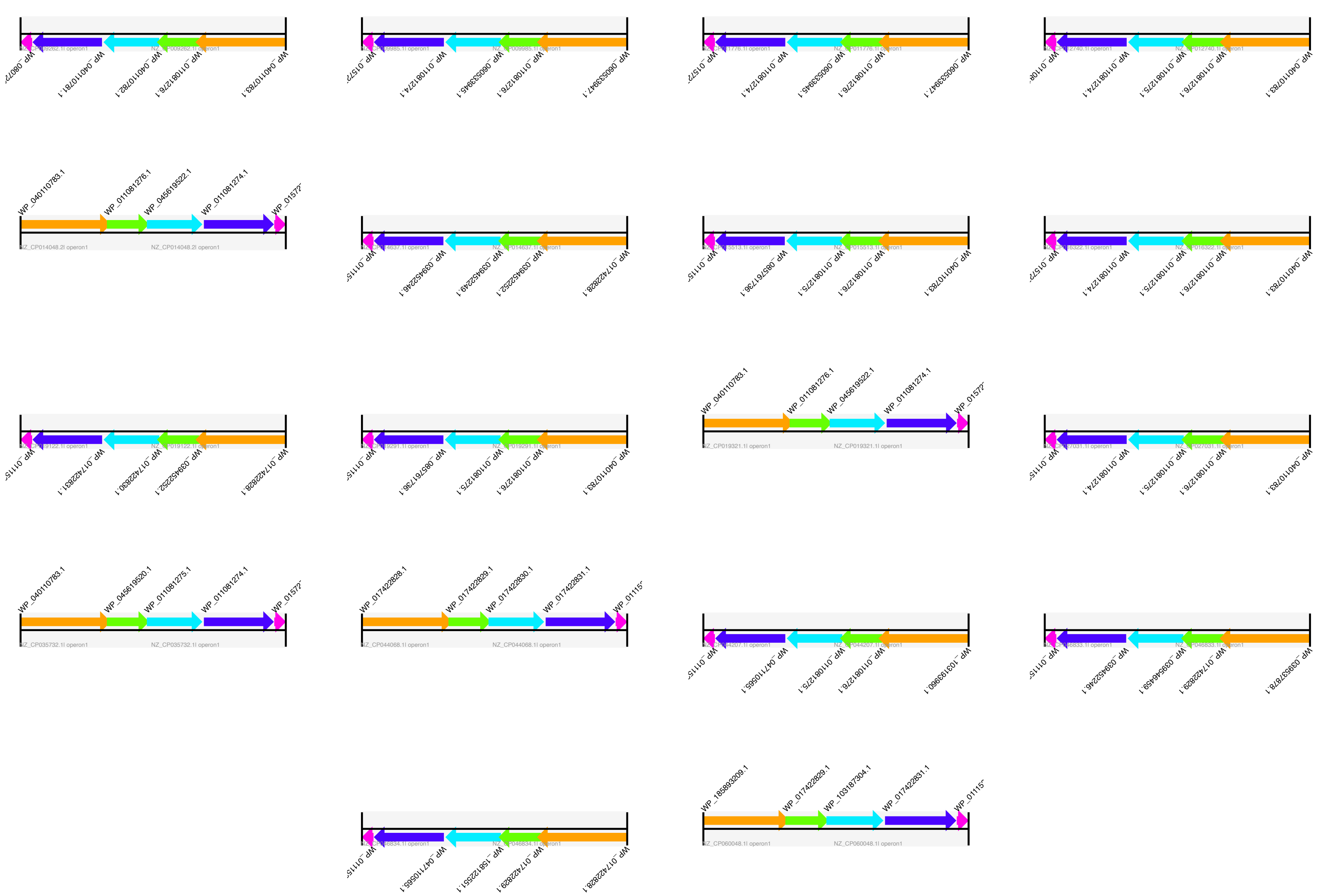

Vibrio_casei_GCF_003335255.1								
**Structural Similarity**	**Average Percent Amino Acid Identity**	**WP_001911723.1**	**WP_000815041.1**	**WP_000240569.1**	**WP_000822678.1**	**WP_000459082.1**	**Taxonomic ID**	**Genome Assembly Accession**
50.0%	45.118225590513234%	56.91202872531418%	75.73729419683931%	34.60847169707929%	0%	58.333333333333336%	673372	GCF_003335255.1

Other Gene | VxrA | VxrB | VxrC | VxrD | VxrE

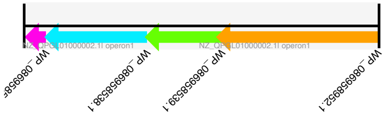

Vibrio_litoralis_DSM_17657_GCF_000426765.1								
**Structural Similarity**	**Average Percent Amino Acid Identity**	**WP_001911723.1**	**WP_000815041.1**	**WP_000240569.1**	**WP_000822678.1**	**WP_000459082.1**	**Taxonomic ID**	**Genome Assembly Accession**
50.0%	45.715715927759106%	54.34195087233347%	72.82848721837502%	34.926203945459825%	0%	66.48193760262726%	1123493	GCF_000426765.1

Other Gene | VxrA | VxrB | VxrC | VxrD | VxrE

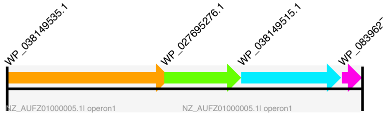

Vibrio_gangliei_GCF_002934045.1								
**Structural Similarity**	**Average Percent Amino Acid Identity**	**WP_001911723.1**	**WP_000815041.1**	**WP_000240569.1**	**WP_000822678.1**	**WP_000459082.1**	**Taxonomic ID**	**Genome Assembly Accession**
50.0%	45.7954956280045%	55.834829443447035%	71.91697041438613%	36.80964477351999%	0%	64.41603350866939%	2077090	GCF_002934045.1

Other Gene | VxrA | VxrB | VxrC | VxrD | VxrE

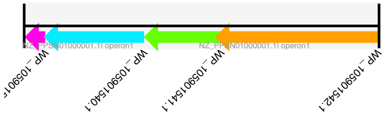

Vibrio_algivorus_GCF_007623795.1								
**Structural Similarity**	**Average Percent Amino Acid Identity**	**WP_001911723.1**	**WP_000815041.1**	**WP_000240569.1**	**WP_000822678.1**	**WP_000459082.1**	**Taxonomic ID**	**Genome Assembly Accession**
50.0%	45.614698303841266%	54.39865140377418%	72.5283624441404%	34.65522396718927%	0%	66.49125370410246%	1667024	GCF_007623795.1

Other Gene VxrA VxrB VxrC VxrD VxrE

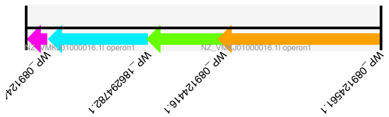

Vibrio_ezurae_GCF_003568985.1								
**Structural Similarity**	**Average Percent Amino Acid Identity**	**WP_001911723.1**	**WP_000815041.1**	**WP_000240569.1**	**WP_000822678.1**	**WP_000459082.1**	**Taxonomic ID**	**Genome Assembly Accession**
50.0%	44.76926483308285%	52.15889464594127%	80.57323695410717%	34.7997348881618%	0%	56.314457677203976%	252583	GCF_003568985.1

Other Gene VxrA VxrB VxrC VxrD VxrE

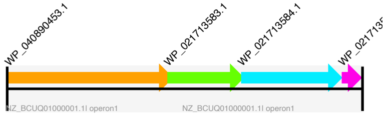

Vibrio_halioticoli_GCF_003568965.1								
**Structural Similarity**	**Average Percent Amino Acid Identity**	**WP_001911723.1**	**WP_000815041.1**	**WP_000240569.1**	**WP_000822678.1**	**WP_000459082.1**	**Taxonomic ID**	**Genome Assembly Accession**
50.0%	44.51198392493099%	52.512998266897746%	78.55732563514599%	35.175138045407266%	0%	56.314457677203976%	71388	GCF_003568965.1

Other Gene | VxrA | VxrB | VxrC | VxrD | VxrE

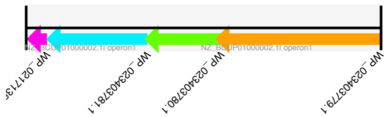

Vibrio_inusitatus_NBRC_102082_GCF_006538525.1								
**Structural Similarity**	**Average Percent Amino Acid Identity**	**WP_001911723.1**	**WP_000815041.1**	**WP_000240569.1**	**WP_000822678.1**	**WP_000459082.1**	**Taxonomic ID**	**Genome Assembly Accession**
50.0%	50.02527394456595%	52.25694444444445%	80.08874813136921%	34.967672921658504%	30.13701143596792%	52.67599278938967%	1219070	GCF_006538525.1

Other Gene | VxrA | VxrB | VxrC | VxrD | VxrE

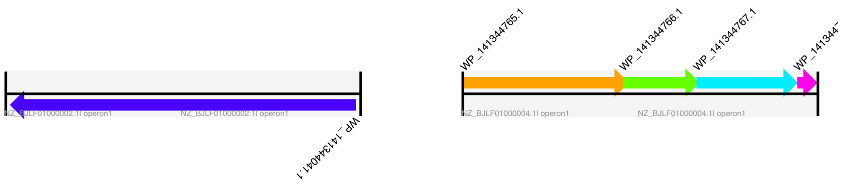

Vibrio_hibernica_GCF_015223435.1								
**Structural Similarity**	**Average Percent Amino Acid Identity**	**WP_001911723.1**	**WP_000815041.1**	**WP_000240569.1**	**WP_000822678.1**	**WP_000459082.1**	**Taxonomic ID**	**Genome Assembly Accession**
50.0%	45.488348940774486%	53.20623916811091%	75.52742616033755%	36.808297776482135%	0%	61.899781598941836%	2587465	GCF_015223435.1

Other Gene | VxrA | VxrB | VxrC | VxrD | VxrE

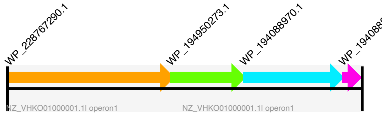

Vibrio_superstes_GCF_003569005.1								
**Structural Similarity**	**Average Percent Amino Acid Identity**	**WP_001911723.1**	**WP_000815041.1**	**WP_000240569.1**	**WP_000822678.1**	**WP_000459082.1**	**Taxonomic ID**	**Genome Assembly Accession**
50.0%	50.1833400612016%	54.24340958113222%	80.00110999084286%	34.271417302441435%	29.724770642201833%	52.67599278938967%	198815	GCF_003569005.1

Other Gene: black; VxrA: orange; VxrB: green; VxrC: cyan; VxrD: blue; VxrE: magenta

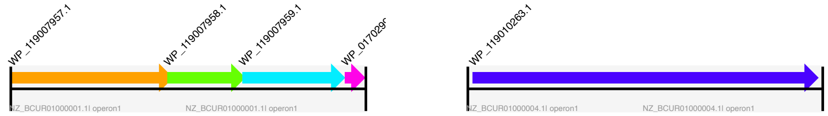

Vibrio_comitans_NBRC_102076_GCF_006538565.1								
**Structural Similarity**	**Average Percent Amino Acid Identity**	**WP_001911723.1**	**WP_000815041.1**	**WP_000240569.1**	**WP_000822678.1**	**WP_000459082.1**	**Taxonomic ID**	**Genome Assembly Accession**
50.0%	50.26143757142285%	53.88418170311148%	80.08808100171358%	34.88085792370373%	29.778074439195816%	52.67599278938967%	1219078	GCF_006538565.1

Other Gene | VxrA | VxrB | VxrC | VxrD | VxrE

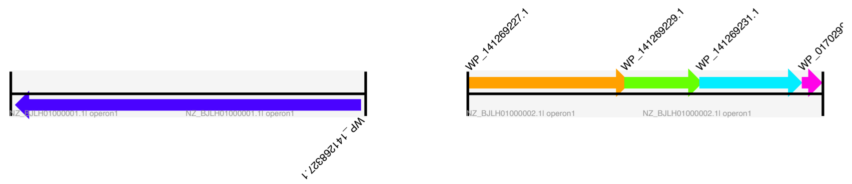

Vibrio_breoganii_GCF_002876585.1								
**Structural Similarity**	**Average Percent Amino Acid Identity**	**WP_001911723.1**	**WP_000815041.1**	**WP_000240569.1**	**WP_000822678.1**	**WP_000459082.1**	**Taxonomic ID**	**Genome Assembly Accession**
50.0%	44.30754782557402%	53.88418170311148%	80.17390339138399%	34.80366124398497%	0%	52.67599278938967%	553239	GCF_002876585.1

Other Gene VxrA VxrB VxrC VxrD VxrE

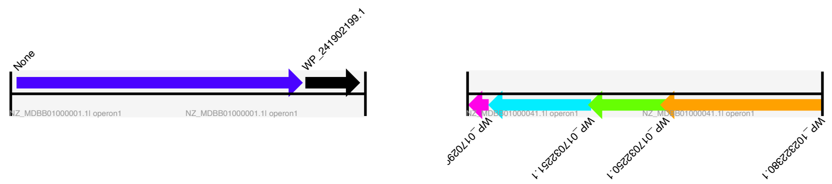

Vibrio_rumoiensis_GCF_002218045.2								
**Structural Similarity**	**Average Percent Amino Acid Identity**	**WP_001911723.1**	**WP_000815041.1**	**WP_000240569.1**	**WP_000822678.1**	**WP_000459082.1**	**Taxonomic ID**	**Genome Assembly Accession**
50.0%	46.23164989294185%	55.695031627446056%	73.54662384615361%	36.031331592689305%	0%	65.88526239842028%	76258	GCF_002218045.2

Other Gene VxrA VxrB VxrC VxrD VxrE

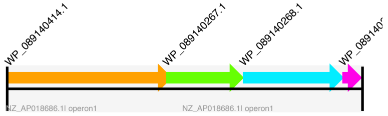

Aliivibrio_finisterrensis_GCF_008933155.1								
**Structural Similarity**	**Average Percent Amino Acid Identity**	**WP_001911723.1**	**WP_000815041.1**	**WP_000240569.1**	**WP_000822678.1**	**WP_000459082.1**	**Taxonomic ID**	**Genome Assembly Accession**
75.0%	48.68586804611185%	49.4097807757167%	69.7677046178769%	32.880149126023724%	37.32569830745169%	54.046007403490215%	511998	GCF_008933155.1

Other Gene VxrA VxrB VxrC VxrD VxrE

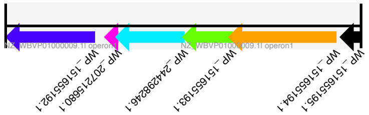

Vibrio_mexicanus_GCF_001012815.1								
**Structural Similarity**	**Average Percent Amino Acid Identity**	**WP_001911723.1**	**WP_000815041.1**	**WP_000240569.1**	**WP_000822678.1**	**WP_000459082.1**	**Taxonomic ID**	**Genome Assembly Accession**
50.0%	38.15888762601184%	0%	84.97225219598748%	47.96699467724113%	0%	57.85519125683061%	1004326	GCF_001012815.1

Other Gene | VxrA | VxrB | VxrC | VxrD | VxrE

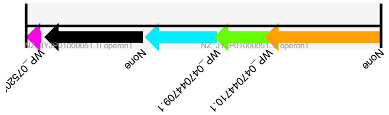

Vibrio_sonorensis_GCF_001854765.1								
**Structural Similarity**	**Average Percent Amino Acid Identity**	**WP_001911723.1**	**WP_000815041.1**	**WP_000240569.1**	**WP_000822678.1**	**WP_000459082.1**	**Taxonomic ID**	**Genome Assembly Accession**
100.0%	49.30896133690054%	0%	87.04184061880848%	46.05634445915712%	56.51680662984715%	56.929814976689975%	1004316	GCF_001854765.1

Other Gene | VxrA | VxrB | VxrC | VxrD | VxrE

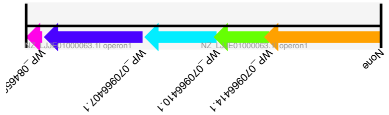

Vibrio_maritimus_GCF_003263775.1								
**Structural Similarity**	**Average Percent Amino Acid Identity**	**WP_001911723.1**	**WP_000815041.1**	**WP_000240569.1**	**WP_000822678.1**	**WP_000459082.1**	**Taxonomic ID**	**Genome Assembly Accession**
0.0%	20.017018794120855%	0%	0%	42.857142857142854%	0%	57.227951113461415%	990268	GCF_003263775.1

Other Gene VxrA VxrB VxrC VxrD VxrE

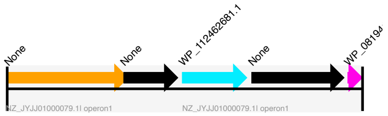

Aliivibrio_fischeri_GCF_004359415.1								
**Structural Similarity**	**Average Percent Amino Acid Identity**	**WP_001911723.1**	**WP_000815041.1**	**WP_000240569.1**	**WP_000822678.1**	**WP_000459082.1**	**Taxonomic ID**	**Genome Assembly Accession**
75.0%	49.42446740621165%	50.847457627118644%	70.49556078326977%	33.62837233090398%	38.23346890454641%	53.91747738521939%	668	GCF_004359415.1

Other Gene | VxrA | VxrB | VxrC | VxrD | VxrE

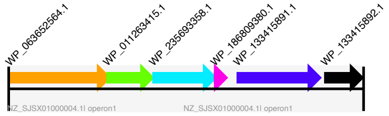

Aliivibrio_logei_GCF_001691055.1								
**Structural Similarity**	**Average Percent Amino Acid Identity**	**WP_001911723.1**	**WP_000815041.1**	**WP_000240569.1**	**WP_000822678.1**	**WP_000459082.1**	**Taxonomic ID**	**Genome Assembly Accession**
75.0%	48.51346873668168%	49.21681525730335%	69.29500350544296%	30.599801045112425%	38.45917473392741%	54.99654914162224%	688	GCF_001691055.1

Other Gene | VxrA | VxrB | VxrC | VxrD | VxrE

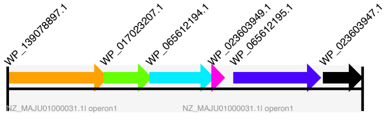

Aliivibrio_sifiae_GCF_002954715.1								
**Structural Similarity**	**Average Percent Amino Acid Identity**	**WP_001911723.1**	**WP_000815041.1**	**WP_000240569.1**	**WP_000822678.1**	**WP_000459082.1**	**Taxonomic ID**	**Genome Assembly Accession**
75.0%	48.50914619327778%	48.986486486486484%	70.79085553328723%	30.578797852927526%	38.466403723838965%	53.72318736984872%	566293	GCF_002954715.1
Other Gene VxrA VxrB VxrC VxrD VxrE								

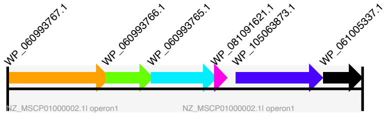

Aliivibrio_salmonicida_LFI1238_GCF_000196495.1								
**Structural Similarity**	**Average Percent Amino Acid Identity**	**WP_001911723.1**	**WP_000815041.1**	**WP_000240569.1**	**WP_000822678.1**	**WP_000459082.1**	**Taxonomic ID**	**Genome Assembly Accession**
75.0%	40.79775326635234%	47.82131975991097%	69.29500350544296%	30.25834836179664%	0%	56.61409470461116%	316275	GCF_000196495.1

Other Gene VxrA VxrB VxrC VxrD VxrE

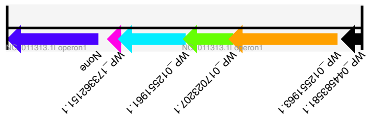

Photobacterium_aquae_GCF_001029445.1								
**Structural Similarity**	**Average Percent Amino Acid Identity**	**WP_001911723.1**	**WP_000815041.1**	**WP_000240569.1**	**WP_000822678.1**	**WP_000459082.1**	**Taxonomic ID**	**Genome Assembly Accession**
50.0%	41.78316132843498%	45.812807881773395%	58.8510887136196%	29.974811083123427%	29.927007299270077%	44.35009166438839%	1195763	GCF_001029445.1

Other Gene VxrA VxrB VxrC VxrD VxrE

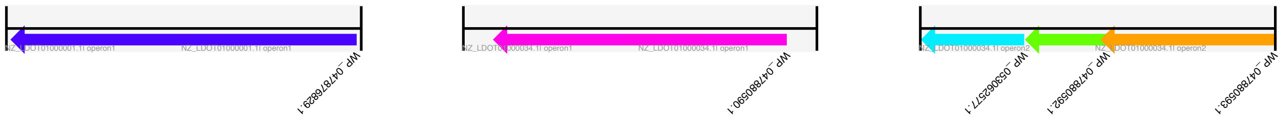

Photobacterium_sanctipauli_GCF_003026495.1								
**Structural Similarity**	**Average Percent Amino Acid Identity**	**WP_001911723.1**	**WP_000815041.1**	**WP_000240569.1**	**WP_000822678.1**	**WP_000459082.1**	**Taxonomic ID**	**Genome Assembly Accession**
50.0%	36.552140796015735%	45.99018003273322%	59.92182921256366%	28.67742329788535%	0%	48.171271436896454%	1342794	GCF_003026495.1

Other Gene VxrA VxrB VxrC VxrD VxrE

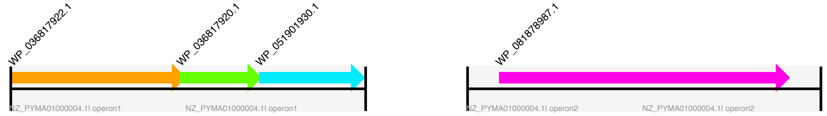

Salinivibrio_costicola_subsp_alcaliphilus_GCF_001996185.1								
**Structural Similarity**	**Average Percent Amino Acid Identity**	**WP_001911723.1**	**WP_000815041.1**	**WP_000240569.1**	**WP_000822678.1**	**WP_000459082.1**	**Taxonomic ID**	**Genome Assembly Accession**
50.0%	26.89527923854491%	45.486797970241675%	60.84697197318379%	28.142626249299074%	0%	0%	272773	GCF_001996185.1

Other Gene | VxrA | VxrB | VxrC | VxrD | VxrE

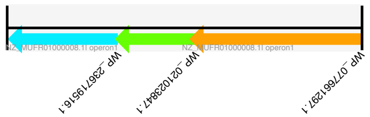

Photobacterium_ganghwense_GCF_003025595.1								
**Structural Similarity**	**Average Percent Amino Acid Identity**	**WP_001911723.1**	**WP_000815041.1**	**WP_000240569.1**	**WP_000822678.1**	**WP_000459082.1**	**Taxonomic ID**	**Genome Assembly Accession**
50.0%	37.49323340951709%	45.394736842105227%	61.588152156100506%	28.850941980942903%	0%	51.6323360684368%	320778	GCF_003025595.1

Other Gene | VxrA | VxrB | VxrC | VxrD | VxrE

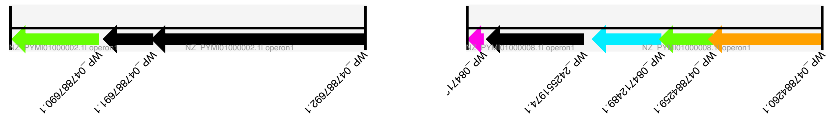

Photobacterium_frigidiphilum_GCF_003025615.1								
**Structural Similarity**	**Average Percent Amino Acid Identity**	**WP_001911723.1**	**WP_000815041.1**	**WP_000240569.1**	**WP_000822678.1**	**WP_000459082.1**	**Taxonomic ID**	**Genome Assembly Accession**
50.0%	35.551541579389585%	45.650335518873625%	60.03967765748032%	29.064117774207176%	0%	43.003576946386815%	264736	GCF_003025615.1

Other Gene | VxrA | VxrB | VxrC | VxrD | VxrE

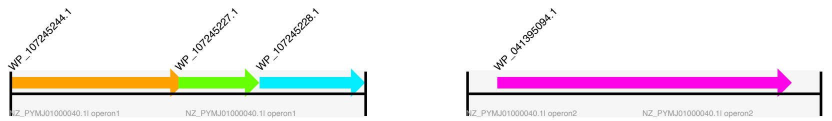

Photobacterium_gaetbulicola_Gung47_GCF_000940995.1								
**Structural Similarity**	**Average Percent Amino Acid Identity**	**WP_001911723.1**	**WP_000815041.1**	**WP_000240569.1**	**WP_000822678.1**	**WP_000459082.1**	**Taxonomic ID**	**Genome Assembly Accession**
50.0%	37.60046246047514%	44.756430188473395%	62.06268426687396%	30.407957462412906%	0%	50.77524038461545%	658445	GCF_000940995.1

Other Gene | VxrA | VxrB | VxrC | VxrD | VxrE

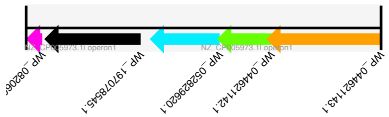

Photobacterium_indicum_GCF_003026215.1								
**Structural Similarity**	**Average Percent Amino Acid Identity**	**WP_001911723.1**	**WP_000815041.1**	**WP_000240569.1**	**WP_000822678.1**	**WP_000459082.1**	**Taxonomic ID**	**Genome Assembly Accession**
50.0%	35.421533780066326%	45.650335518873625%	59.88225082652857%	28.571505608542637%	0%	43.003576946386815%	81447	GCF_003026215.1
Other Gene VxrA VxrB VxrC VxrD VxrE								

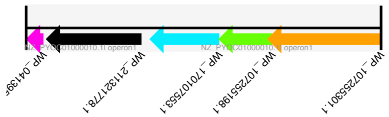

Photobacterium_profundum_GCF_003026285.1								
**Structural Similarity**	**Average Percent Amino Acid Identity**	**WP_001911723.1**	**WP_000815041.1**	**WP_000240569.1**	**WP_000822678.1**	**WP_000459082.1**	**Taxonomic ID**	**Genome Assembly Accession**
50.0%	34.45648350416732%	45.40939193257074%	59.48828865003824%	28.453690534938975%	0%	38.93104640328864%	74109	GCF_003026285.1

Other Gene VxrA VxrB VxrC VxrD VxrE

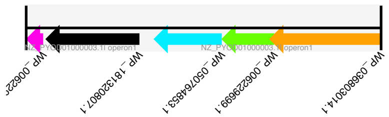

Photobacterium_aquimaris_GCF_900185565.1								
**Structural Similarity**	**Average Percent Amino Acid Identity**	**WP_001911723.1**	**WP_000815041.1**	**WP_000240569.1**	**WP_000822678.1**	**WP_000459082.1**	**Taxonomic ID**	**Genome Assembly Accession**
50.0%	35.44180445031417%	44.57236842105263%	59.96125208250387%	30.71165835411471%	0%	41.963743393899655%	512643	GCF_900185565.1

Other Gene | VxrA | VxrB | VxrC | VxrD | VxrE

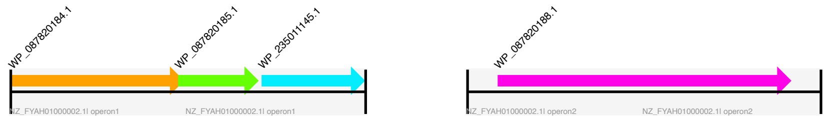

Photobacterium_jeanii_GCF_003025495.1								
**Structural Similarity**	**Average Percent Amino Acid Identity**	**WP_001911723.1**	**WP_000815041.1**	**WP_000240569.1**	**WP_000822678.1**	**WP_000459082.1**	**Taxonomic ID**	**Genome Assembly Accession**
50.0%	33.796380263613045%	44.42622950819672%	60.39047565648874%	30.800363587923236%	33.3648325654565545%	0%	858640	GCF_003025495.1
Other Gene VxrA VxrB VxrC VxrD VxrE								

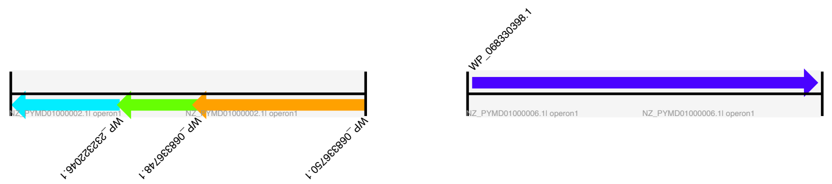

Photobacterium_kishitanii_GCF_003026635.1								
**Structural Similarity**	**Average Percent Amino Acid Identity**	**WP_001911723.1**	**WP_000815041.1**	**WP_000240569.1**	**WP_000822678.1**	**WP_000459082.1**	**Taxonomic ID**	**Genome Assembly Accession**
50.0%	36.60458661329271%	44.97528830313015%	59.96125208250387%	29.966749792186192%	0%	48.11964288864335%	318456	GCF_003026635.1

Other Gene, VxrA, VxrB, VxrC, VxrD, VxrE

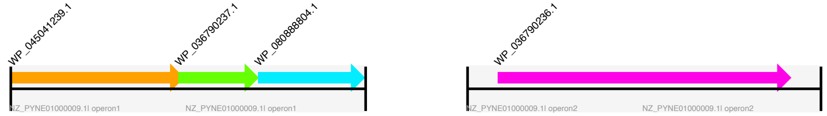

Photobacterium_andalusiense_GCF_900185625.1								
**Structural Similarity**	**Average Percent Amino Acid Identity**	**WP_001911723.1**	**WP_000815041.1**	**WP_000240569.1**	**WP_000822678.1**	**WP_000459082.1**	**Taxonomic ID**	**Genome Assembly Accession**
50.0%	35.143407838374664%	44.42622950819673%	59.56677016090845%	29.760296128868475%	0%	41.963743393899655%	2204296	GCF_900185625.1

Other Gene, VxrA, VxrB, VxrC, VxrD, VxrE

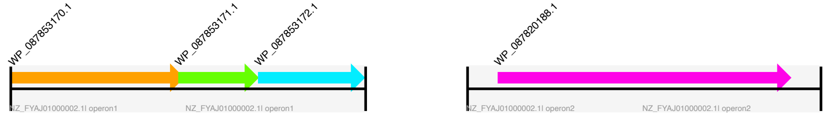

Photobacterium_malacitanum_GCF_900185615.1								
**Structural Similarity**	**Average Percent Amino Acid Identity**	**WP_001911723.1**	**WP_000815041.1**	**WP_000240569.1**	**WP_000822678.1**	**WP_000459082.1**	**Taxonomic ID**	**Genome Assembly Accession**
50.0%	35.80527142175135%	44.499178981937604%	59.96125208250387%	29.804599757489015%	0%	44.76132628682626%	2204294	GCF_900185615.1

Other Gene VxrA VxrB VxrC VxrD VxrE

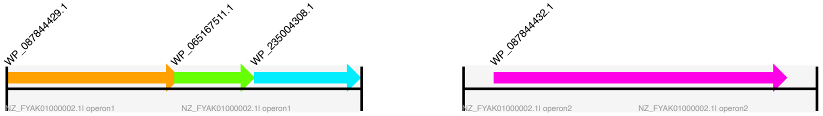

Photobacterium_swingsii_GCF_003026435.1								
**Structural Similarity**	**Average Percent Amino Acid Identity**	**WP_001911723.1**	**WP_000815041.1**	**WP_000240569.1**	**WP_000822678.1**	**WP_000459082.1**	**Taxonomic ID**	**Genome Assembly Accession**
50.0%	42.791668815803675%	44.44444444444445%	59.923626621776116%	32.791040100250626%	34.32415284218493%	42.475080070362246%	680026	GCF_003026435.1

Other Gene | VxrA | VxrB | VxrC | VxrD | VxrE

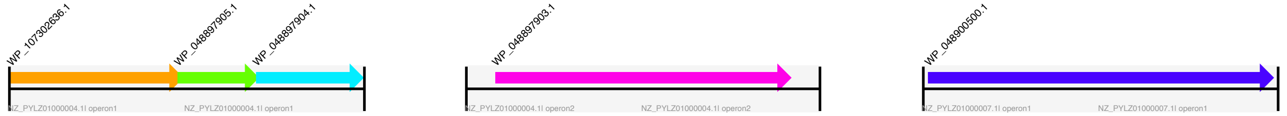

Salinivibrio_socompensis_S10B_GCF_000565325.1								
**Structural Similarity**	**Average Percent Amino Acid Identity**	**WP_001911723.1**	**WP_000815041.1**	**WP_000240569.1**	**WP_000822678.1**	**WP_000459082.1**	**Taxonomic ID**	**Genome Assembly Accession**
75.0%	25.73136813598046%	45.17195659316461%	60.70584080655546%	0%	22.779043280182233%	0%	868280	GCF_000565325.1

Other Gene VxrA VxrB VxrC VxrD VxrE

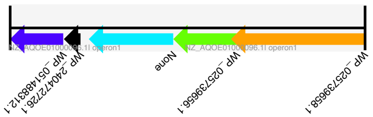

Photobacterium_toruni_GCF_900166975.1								
**Structural Similarity**	**Average Percent Amino Acid Identity**	**WP_001911723.1**	**WP_000815041.1**	**WP_000240569.1**	**WP_000822678.1**	**WP_000459082.1**	**Taxonomic ID**	**Genome Assembly Accession**
50.0%	36.14054791826852%	44.29752066115702%	60.31793523565565%	31.017369727047146%	0%	45.06991396748276%	1935446	GCF_900166975.1

Other Gene VxrA VxrB VxrC VxrD VxrE

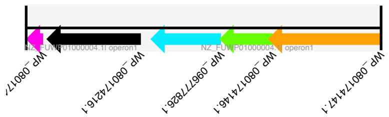

Photobacterium_iliopiscarium_GCF_003025455.1								
**Structural Similarity**	**Average Percent Amino Acid Identity**	**WP_001911723.1**	**WP_000815041.1**	**WP_000240569.1**	**WP_000822678.1**	**WP_000459082.1**	**Taxonomic ID**	**Genome Assembly Accession**
50.0%	36.37327764348423%	44.884488448844884%	60.07952069232453%	30.198019801980198%	0%	46.70435927427152%	56192	GCF_003025455.1

Other Gene VxrA VxrB VxrC VxrD VxrE

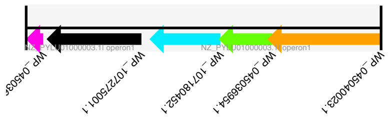

Thaumasiovibrio_subtropicus_GCF_002157755.1								
**Structural Similarity**	**Average Percent Amino Acid Identity**	**WP_001911723.1**	**WP_000815041.1**	**WP_000240569.1**	**WP_000822678.1**	**WP_000459082.1**	**Taxonomic ID**	**Genome Assembly Accession**
50.0%	26.374049793567213%	44.57900335488094%	58.92586099757048%	28.365384615384613%	0%	0%	1891207	GCF_002157755.1

Other Gene | VxrA | VxrB | VxrC | VxrD | VxrE

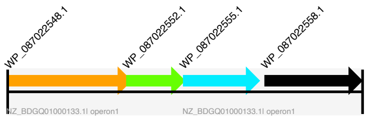

Photobacterium_piscicola_GCF_900166965.1								
**Structural Similarity**	**Average Percent Amino Acid Identity**	**WP_001911723.1**	**WP_000815041.1**	**WP_000240569.1**	**WP_000822678.1**	**WP_000459082.1**	**Taxonomic ID**	**Genome Assembly Accession**
50.0%	36.106783388143214%	44.64579901153213%	60.07952069232453%	29.756097560975608%	0%	46.052499675883816%	1378299	GCF_900166965.1

Other Gene | VxrA | VxrB | VxrC | VxrD | VxrE

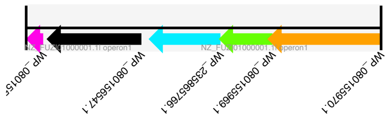

Salinivibrio_proteolyticus_GCF_001996165.1								
**Structural Similarity**	**Average Percent Amino Acid Identity**	**WP_001911723.1**	**WP_000815041.1**	**WP_000240569.1**	**WP_000822678.1**	**WP_000459082.1**	**Taxonomic ID**	**Genome Assembly Accession**
75.0%	33.065760844735024%	45.01953968974159%	60.664934776737056%	26.658518130492986%	32.985811626703466%	0%	334715	GCF_001996165.1

Other Gene VxrA VxrB VxrC VxrD VxrE

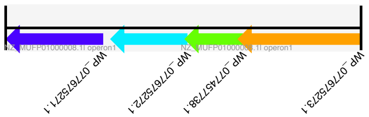

Photobacterium_phosphoreum_GCF_002954725.1								
**Structural Similarity**	**Average Percent Amino Acid Identity**	**WP_001911723.1**	**WP_000815041.1**	**WP_000240569.1**	**WP_000822678.1**	**WP_000459082.1**	**Taxonomic ID**	**Genome Assembly Accession**
50.0%	35.69284688884344%	44.511885866491525%	59.84298347268321%	30.866909713199846%	0%	43.242455391842626%	659	GCF_002954725.1
Other Gene ■ VxrA ■ VxrB ■ VxrC ■ VxrD ■ VxrE ■								

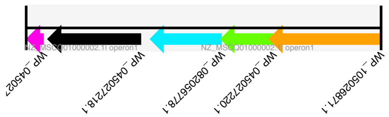

Photobacterium_lipolyticum_GCF_003026475.1								
**Structural Similarity**	**Average Percent Amino Acid Identity**	**WP_001911723.1**	**WP_000815041.1**	**WP_000240569.1**	**WP_000822678.1**	**WP_000459082.1**	**Taxonomic ID**	**Genome Assembly Accession**
50.0%	36.02794113891191%	45.936981757877284%	59.49142156862746%	30.364473680461977%	0%	44.34682868759285%	266810	GCF_003026475.1

Other Gene VxrA VxrB VxrC VxrD VxrE

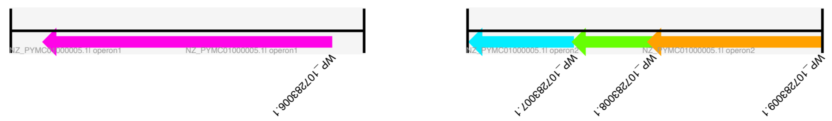

Photobacterium_sanguinicancri_GCF_002265265.1								
**Structural Similarity**	**Average Percent Amino Acid Identity**	**WP_001911723.1**	**WP_000815041.1**	**WP_000240569.1**	**WP_000822678.1**	**WP_000459082.1**	**Taxonomic ID**	**Genome Assembly Accession**
50.0%	43.86958218652015%	44.82758620689655%	60.5052380070195%	33.35021097046414%	34.06707230246695%	46.59780344575359%	875932	GCF_002265265.1

Other Gene | VxrA | VxrB | VxrC | VxrD | VxrE

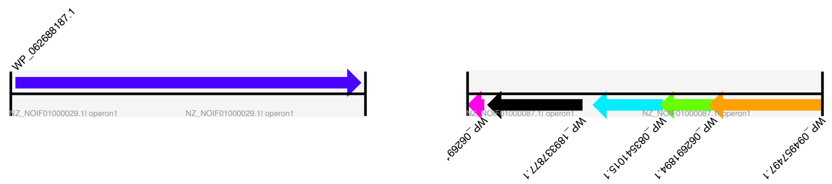

Photobacterium_aphoticum_GCF_001029435.1								
**Structural Similarity**	**Average Percent Amino Acid Identity**	**WP_001911723.1**	**WP_000815041.1**	**WP_000240569.1**	**WP_000822678.1**	**WP_000459082.1**	**Taxonomic ID**	**Genome Assembly Accession**
50.0%	36.650058316280926%	44.85049833887043%	59.74843629277209%	30.30307482545429%	0%	48.348282124307836%	754436	GCF_001029435.1

Other Gene | VxrA | VxrB | VxrC | VxrD | VxrE

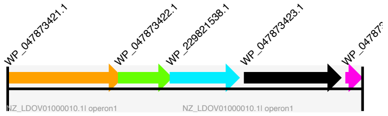

Salinivibrio_sharmensis_GCF_001995985.1								
**Structural Similarity**	**Average Percent Amino Acid Identity**	**WP_001911723.1**	**WP_000815041.1**	**WP_000240569.1**	**WP_000822678.1**	**WP_000459082.1**	**Taxonomic ID**	**Genome Assembly Accession**
75.0%	33.92029483729783%	45.09920781743597%	61.24925544450552%	27.890659072834094%	35.362351851713555%	0%	390883	GCF_001995985.1
Other Gene VxrA VxrB VxrC VxrD VxrE								

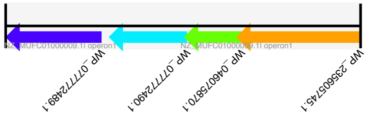

Photobacterium_lucens_GCF_009874335.1								
**Structural Similarity**	**Average Percent Amino Acid Identity**	**WP_001911723.1**	**WP_000815041.1**	**WP_000240569.1**	**WP_000822678.1**	**WP_000459082.1**	**Taxonomic ID**	**Genome Assembly Accession**
50.0%	35.91436923349694%	45.73979216103428%	56.92883895131086%	30.517621802134897%	0%	46.38559325300466%	2562949	GCF_009874335.1

Other Gene ■ VxrA ■ VxrB ■ VxrC ■ VxrD ■ VxrE ■

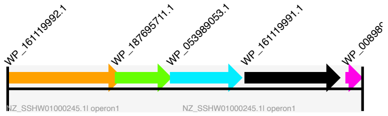

Enterovibrio_baiacu_GCF_004120195.1								
**Structural Similarity**	**Average Percent Amino Acid Identity**	**WP_001911723.1**	**WP_000815041.1**	**WP_000240569.1**	**WP_000822678.1**	**WP_000459082.1**	**Taxonomic ID**	**Genome Assembly Accession**
50.0%	36.32038031244647%	45.666666666666664%	60.03846041355912%	29.611650485543689%	0%	46.28512399656965%	2491023	GCF_004120195.1

Other Gene VxrA VxrB VxrC VxrD VxrE

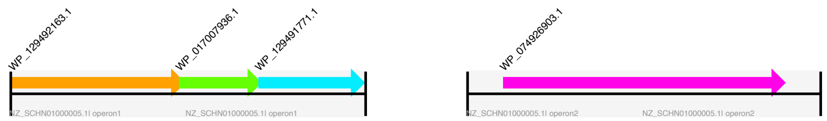

Photobacterium_carnosum_GCF_002849605.1								
**Structural Similarity**	**Average Percent Amino Acid Identity**	**WP_001911723.1**	**WP_000815041.1**	**WP_000240569.1**	**WP_000822678.1**	**WP_000459082.1**	**Taxonomic ID**	**Genome Assembly Accession**
50.0%	42.866938091485125%	44.51775490563883%	60.07952069232453%	29.925187032418954%	33.107868552771784%	46.70435927427152%	2023717	GCF_002849605.1

Other Gene VxrA VxrB VxrC VxrD VxrE

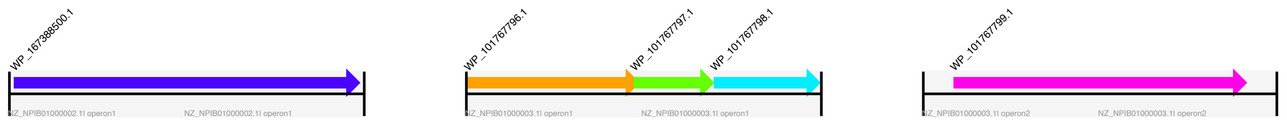

Salinivibrio_siamensis_GCF_001996005.1

Structural Similarity	Average Percent Amino Acid Identity	WP_001911723.1	WP_000815041.1	WP_000240569.1	WP_000822678.1	WP_000459082.1	Taxonomic ID	Genome Assembly Accession
75.0%	33.60792390452528%	43.986820428336074%	61.24925544450552%	28.221379173186538%	34.58216447659825%	0%	414286	GCF_001996005.1

Other Gene | VxrA | VxrB | VxrC | VxrD | VxrE

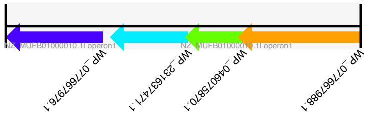

Enterovibrio_pacificus_GCF_001707825.1								
**Structural Similarity**	**Average Percent Amino Acid Identity**	**WP_001911723.1**	**WP_000815041.1**	**WP_000240569.1**	**WP_000822678.1**	**WP_000459082.1**	**Taxonomic ID**	**Genome Assembly Accession**
50.0%	32.748310462934725%	43.67816091954024%	59.58919971671216%	28.37576106237115%	32.09843061605012%	0%	1080227	GCF_001707825.1

Other Gene | VxrA | VxrB | VxrC | VxrD | VxrE

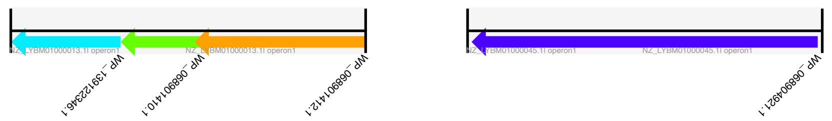

Enterovibrio_norvegicus_GCF_900537165.1								
**Structural Similarity**	**Average Percent Amino Acid Identity**	**WP_001911723.1**	**WP_000815041.1**	**WP_000240569.1**	**WP_000822678.1**	**WP_000459082.1**	**Taxonomic ID**	**Genome Assembly Accession**
50.0%	36.10536728852982%	45.666666666666664%	60.03846041355912%	28.536585365853657%	0%	46.28512399656965%	188144	GCF_900537165.1

Other Gene | VxrA | VxrB | VxrC | VxrD | VxrE

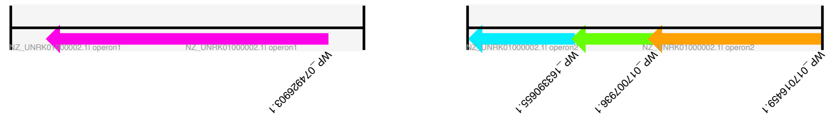

Photobacterium_leiognathi_subsp_mandapamensis_svers_1_1__GCF_000211495.1								
**Structural Similarity**	**Average Percent Amino Acid Identity**	**WP_001911723.1**	**WP_000815041.1**	**WP_000240569.1**	**WP_000822678.1**	**WP_000459082.1**	**Taxonomic ID**	**Genome Assembly Accession**
50.0%	35.81016632368015%	45.49453297883331%	58.33383412332981%	28.836871263232954%	0%	46.38559325300466%	1001530	GCF_000211495.1

Other Gene VxrA VxrB VxrC VxrD VxrE

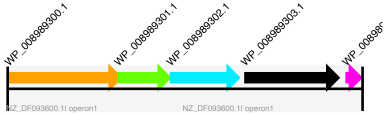

Thaumasiovibrio_occultus_GCF_002157795.1								
**Structural Similarity**	**Average Percent Amino Acid Identity**	**WP_001911723.1**	**WP_000815041.1**	**WP_000240569.1**	**WP_000822678.1**	**WP_000459082.1**	**Taxonomic ID**	**Genome Assembly Accession**
50.0%	26.982040073741963%	43.289885003897496%	62.01738441022472%	29.602930954587585%	0%	0%	1891184	GCF_002157795.1

Other Gene VxrA VxrB VxrC VxrD VxrE

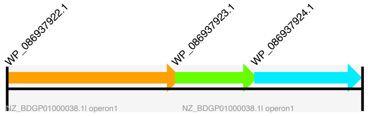

Photobacterium_rosenbergii_GCF_003026455.1								
**Structural Similarity**	**Average Percent Amino Acid Identity**	**WP_001911723.1**	**WP_000815041.1**	**WP_000240569.1**	**WP_000822678.1**	**WP_000459082.1**	**Taxonomic ID**	**Genome Assembly Accession**
50.0%	37.78785118595135%	44.66338259441708%	61.35233988476602%	30.609649417939572%	0%	52.31388403263407%	294936	GCF_003026455.1

Other Gene VxrA VxrB VxrC VxrD VxrE

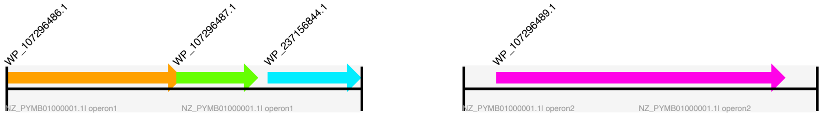

Salinivibrio_kushneri_GCF_005280275.1

Structural Similarity	Average Percent Amino Acid Identity	WP_001911723.1	WP_000815041.1	WP_000240569.1	WP_000822678.1	WP_000459082.1	Taxonomic ID	Genome Assembly Accession
50.0%	26.922998527679063%	44.54309264540663%	61.88079256410164%	28.191107428887047%	0%	0%	1908198	GCF_005280275.1

Other Gene ■ VxrA ■ VxrB ■ VxrC ■ VxrD ■ VxrE ■

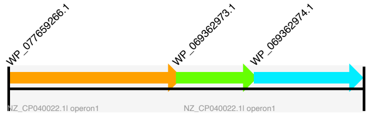

Photobacterium_alginatilyticum_GCF_009910675.1								
**Structural Similarity**	**Average Percent Amino Acid Identity**	**WP_001911723.1**	**WP_000815041.1**	**WP_000240569.1**	**WP_000822678.1**	**WP_000459082.1**	**Taxonomic ID**	**Genome Assembly Accession**
50.0%	36.95974744480854%	45.77114427860697%	56.043956043956044%	28.000000000000004%	0%	54.98363690147969%	1775171	GCF_009910675.1

Other Gene ■ VxrA ■ VxrB ■ VxrC ■ VxrD ■ VxrE ■

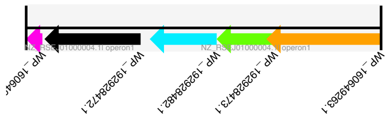

Photobacterium_angustum_GCF_003026895.1								
**Structural Similarity**	**Average Percent Amino Acid Identity**	**WP_001911723.1**	**WP_000815041.1**	**WP_000240569.1**	**WP_000822678.1**	**WP_000459082.1**	**Taxonomic ID**	**Genome Assembly Accession**
50.0%	35.73695160689414%	45.033112582781456%	59.64620632109685%	28.629728174888534%	0%	45.375710955703845%	661	GCF_003026895.1

Other Gene VxrA VxrB VxrC VxrD VxrE

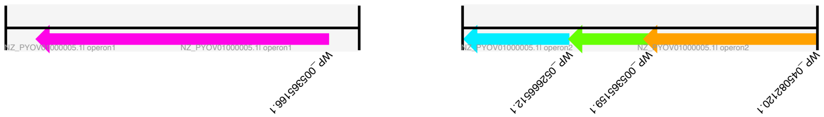

Enterovibrio_nigricans_DSM_22720_GCF_900167155.1								
**Structural Similarity**	**Average Percent Amino Acid Identity**	**WP_001911723.1**	**WP_000815041.1**	**WP_000240569.1**	**WP_000822678.1**	**WP_000459082.1**	**Taxonomic ID**	**Genome Assembly Accession**
100.0%	43.51909779351955%	44.42622950819673%	60.89875550289039%	29.567307692307693%	35.66721896712043%	47.03597729708252%	1121868	GCF_900167155.1
Other Gene	VxrA	VxrB	VxrC	VxrD	VxrE			

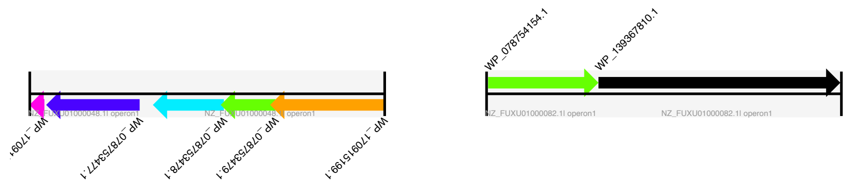

Photobacterium_proteolyticum_GCF_001939735.1

Structural Similarity	Average Percent Amino Acid Identity	WP_001911723.1	WP_000815041.1	WP_000240569.1	WP_000822678.1	WP_000459082.1	Taxonomic ID	Genome Assembly Accession
50.0%	37.776887502041106%	45.70957095709571%	58.245750102651215%	28.818768465110693%	0%	56.11034798534792%	1903952	GCF_001939735.1

Other Gene ■ VxrA ■ VxrB ■ VxrC ■ VxrD ■ VxrE ■

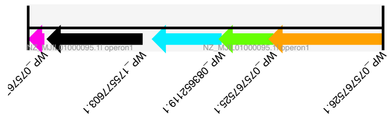

Enterovibrio_coralii_GCF_001559595.1								
**Structural Similarity**	**Average Percent Amino Acid Identity**	**WP_001911723.1**	**WP_000815041.1**	**WP_000240569.1**	**WP_000822678.1**	**WP_000459082.1**	**Taxonomic ID**	**Genome Assembly Accession**
100.0%	43.161939484116296%	44.38943894389439%	60.890913618803836%	29.197166714775356%	35.91813109764887%	45.41404704545901%	294935	GCF_001559595.1

Other Gene | VxrA | VxrB | VxrC | VxrD | VxrE

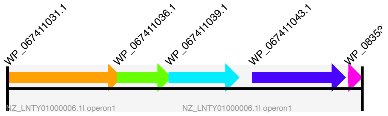

Photobacterium_halotolerans_GCF_009904675.1								
**Structural Similarity**	**Average Percent Amino Acid Identity**	**WP_001911723.1**	**WP_000815041.1**	**WP_000240569.1**	**WP_000822678.1**	**WP_000459082.1**	**Taxonomic ID**	**Genome Assembly Accession**
50.0%	42.88821807571832%	45.107794361525706%	58.57334864437461%	31.25%	30.297397769516728%	49.21254960317456%	265726	GCF_009904675.1

Other Gene | VxrA | VxrB | VxrC | VxrD | VxrE

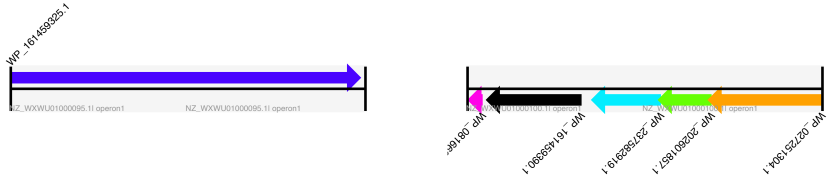

Grimontia_hollisae_GCF_001558255.2								
**Structural Similarity**	**Average Percent Amino Acid Identity**	**WP_001911723.1**	**WP_000815041.1**	**WP_000240569.1**	**WP_000822678.1**	**WP_000459082.1**	**Taxonomic ID**	**Genome Assembly Accession**
50.0%	36.02548175963102%	43.606557377049185%	58.02841597689028%	30.413625304136254%	0%	48.0788101400794%	673	GCF_001558255.2

Other Gene | VxrA | VxrB | VxrC | VxrD | VxrE

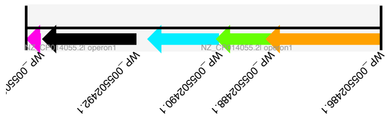

Enterovibrio_calviensis_DSM_14347_GCF_000621165.1								
**Structural Similarity**	**Average Percent Amino Acid Identity**	**WP_001911723.1**	**WP_000815041.1**	**WP_000240569.1**	**WP_000822678.1**	**WP_000459082.1**	**Taxonomic ID**	**Genome Assembly Accession**
75.0%	33.87781971944574%	43.256578947368425%	59.39148490197547%	30.676328502415455%	36.06470624546933%	0%	1121867	GCF_000621165.1

Other Gene VxrA VxrB VxrC VxrD VxrE

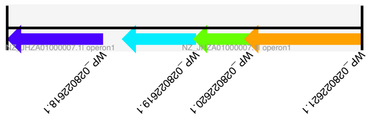

Photobacterium_lutimaris_GCF_003025555.1								
**Structural Similarity**	**Average Percent Amino Acid Identity**	**WP_001911723.1**	**WP_000815041.1**	**WP_000240569.1**	**WP_000822678.1**	**WP_000459082.1**	**Taxonomic ID**	**Genome Assembly Accession**
50.0%	37.09516233862533%	44.35363762991537%	61.6378075267887%	30.247769799825935%	0%	49.23659673659667%	388278	GCF_003025555.1

Other Gene | VxrA | VxrB | VxrC | VxrD | VxrE

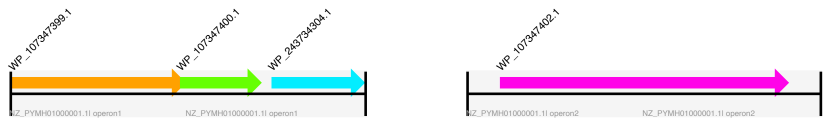

Photobacterium_marinum_GCF_000331515.1								
**Structural Similarity**	**Average Percent Amino Acid Identity**	**WP_001911723.1**	**WP_000815041.1**	**WP_000240569.1**	**WP_000822678.1**	**WP_000459082.1**	**Taxonomic ID**	**Genome Assembly Accession**
50.0%	38.0953325016707%	45.28925619834711%	59.884517151959%	29.292929292929294%	0%	56.0099598651181%	1056511	GCF_000331515.1
Other Gene	VxrA	VxrB	VxrC	VxrD	VxrE			

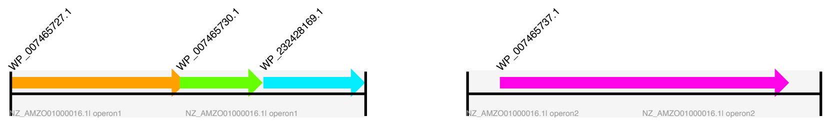

Grimontia_celer_GCF_900055185.1								
**Structural Similarity**	**Average Percent Amino Acid Identity**	**WP_001911723.1**	**WP_000815041.1**	**WP_000240569.1**	**WP_000822678.1**	**WP_000459082.1**	**Taxonomic ID**	**Genome Assembly Accession**
50.0%	35.07346061651059%	43.53518821603928%	57.59797994291145%	29.545049685534984%	0%	44.689085238067236%	1796497	GCF_900055185.1

Other Gene | VxrA | VxrB | VxrC | VxrD | VxrE

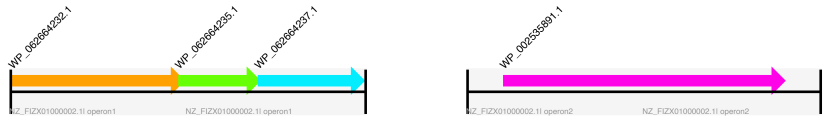

Grimontia_marina_GCF_900060185.1								
**Structural Similarity**	**Average Percent Amino Acid Identity**	**WP_001911723.1**	**WP_000815041.1**	**WP_000240569.1**	**WP_000822678.1**	**WP_000459082.1**	**Taxonomic ID**	**Genome Assembly Accession**
50.0%	26.259707685606497%	43.9344262295082%	58.19744553185762%	29.166666666666668%	0%	0%	646534	GCF_900060185.1

Other Gene | VxrA | VxrB | VxrC | VxrD | VxrE

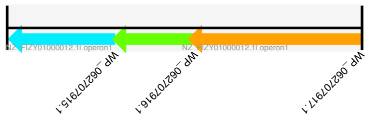

Photobacterium_damselae_GCF_014525015.1								
**Structural Similarity**	**Average Percent Amino Acid Identity**	**WP_001911723.1**	**WP_000815041.1**	**WP_000240569.1**	**WP_000822678.1**	**WP_000459082.1**	**Taxonomic ID**	**Genome Assembly Accession**
50.0%	37.72174866715818%	45.78512396694215%	58.55513307984791%	28.675619493066872%	0%	55.59286679593396%	38293	GCF_014525015.1

Other Gene | VxrA | VxrB | VxrC | VxrD | VxrE

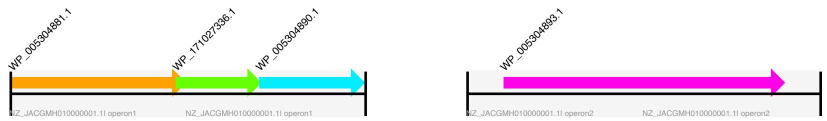

Grimontia_indica_GCF_000333895.2								
**Structural Similarity**	**Average Percent Amino Acid Identity**	**WP_001911723.1**	**WP_000815041.1**	**WP_000240569.1**	**WP_000822678.1**	**WP_000459082.1**	**Taxonomic ID**	**Genome Assembly Accession**
50.0%	35.46534492803003%	43.69885433715221%	58.47195459810022%	30.46683046683047%	0%	44.6890852380672236%	1056512	GCF_000333895.2

Other Gene | VxrA | VxrB | VxrC | VxrD | VxrE

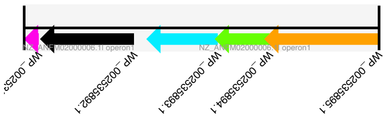

Paraphotobacterium_marinum_GCF_002216855.1								
**Structural Similarity**	**Average Percent Amino Acid Identity**	**WP_001911723.1**	**WP_000815041.1**	**WP_000240569.1**	**WP_000822678.1**	**WP_000459082.1**	**Taxonomic ID**	**Genome Assembly Accession**
50.0%	25.63069994304256%	41.7782778417719444%	58.7309911042626%	27.64423076923077%	0%	0%	1755811	GCF_002216855.1
Other Gene ■ VxrA ■ VxrB ■ VxrC ■ VxrD ■ VxrE ■								

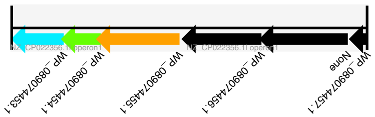

Supplement: S2 Data — (ZIP) [file ppat.1011415.s011.zip › vxrABCDE_vibrionales135623_table.pdf]
