# Supplementary material for: The Rvv two-component regulatory system regulates biofilm formation and colonization in Vibrio cholerae
Source: PLoS Pathog. 2023 May 22;19(5):e1011415. doi: 10.1371/journal.ppat.1011415 (PMC10237652; doi:10.1371/journal.ppat.1011415)

Vibrio_cholerae_MS6_GCF_000829215.1						
**Structural Similarity**	**Average Percent Amino Acid Identity**	**WP_000173586.1**	**WP_000821694.1**	**WP_000562692.1**	**Taxonomic ID**	**Genome Assembly Accession**
100.0%	100.0%	100.0%	100.0%	100.0%	1420885	GCF_000829215.1
Other Gene ■ RvvA ■ RvvB ■ RvvC ■						

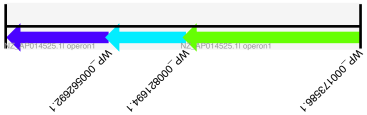

Vibrio_vulnificus_YJ016_GCF_000009745.1						
**Structural Similarity**	**Average Percent Amino Acid Identity**	**WP_000173586.1**	**WP_000821694.1**	**WP_000562692.1**	**Taxonomic ID**	**Genome Assembly Accession**
100.0%	94.58064717392853%	95.4432967927971%	98.15668202764977%	90.14196270133871%	196600	GCF_000009745.1

Other Gene | RvvA | RvvB | RvvC

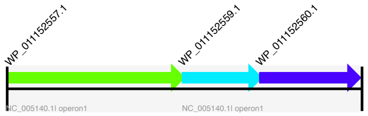

Vibrio_metoecus_GCF_009665275.1						
**Structural Similarity**	**Average Percent Amino Acid Identity**	**WP_000173586.1**	**WP_000821694.1**	**WP_000562692.1**	**Taxonomic ID**	**Genome Assembly Accession**
100.0%	93.5046640507727%	92.43500297381837%	98.1651376146789%	89.91385156382088%	1481663	GCF_009665275.1

Other Gene | RvvA | RvvB | RvvC

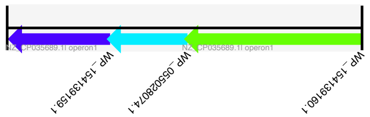

Vibrio_vulnificus_GCF_002204915.1						
**Structural Similarity**	**Average Percent Amino Acid Identity**	**WP_000173586.1**	**WP_000821694.1**	**WP_000562692.1**	**Taxonomic ID**	**Genome Assembly Accession**
100.0%	93.50402203983792%	92.79139187264921%	98.15668202764977%	89.56399221921474%	672	GCF_002204915.1

Other Gene RvvA RvvB RvvC

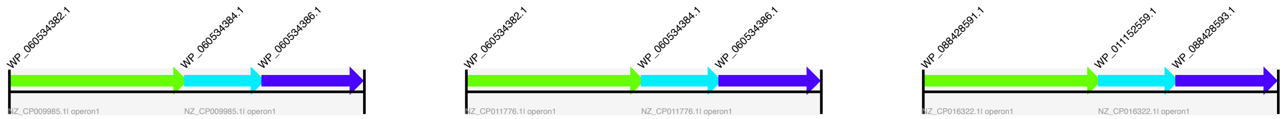

Vibrio_cidicii_GCF_001597945.1						
**Structural Similarity**	**Average Percent Amino Acid Identity**	**WP_000173586.1**	**WP_000821694.1**	**WP_000562692.1**	**Taxonomic ID**	**Genome Assembly Accession**
100.0%	93.34834889110262%	92.12257356884265%	97.69585253456622%	90.22662056990299%	1763883	GCF_001597945.1

Other Gene | RvvA | RvvB | RvvC

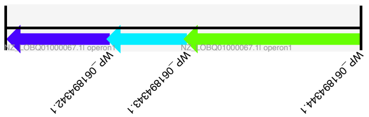

Vibrio_navarrensis_GCF_012275065.1						
**Structural Similarity**	**Average Percent Amino Acid Identity**	**WP_000173586.1**	**WP_000821694.1**	**WP_000562692.1**	**Taxonomic ID**	**Genome Assembly Accession**
100.0%	93.40006532654176%	89.88883005104718%	99.07407407407408%	91.23729185450405%	29495	GCF_012275065.1
Other Gene ■ RvvA ■ RvvB ■ RvvC ■						

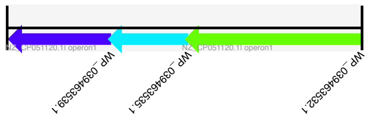

Vibrio_vulnificus_GCF_001433435.1						
**Structural Similarity**	**Average Percent Amino Acid Identity**	**WP_000173586.1**	**WP_000821694.1**	**WP_000562692.1**	**Taxonomic ID**	**Genome Assembly Accession**
100.0%	88.03210842453218%	85.04189495352071%	96.99820788530467%	82.0562224347712%	672	GCF_001433435.1

Other Gene | RvvA | RvvB | RvvC

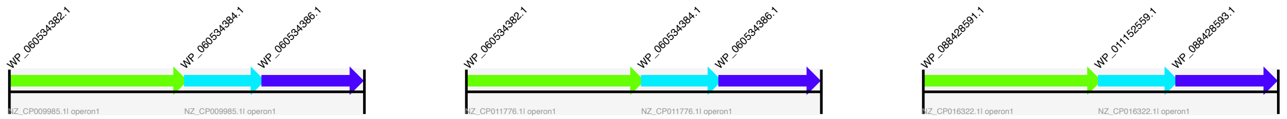

Vibrio_vulnificus_GCF_001653775.1						
**Structural Similarity**	**Average Percent Amino Acid Identity**	**WP_000173586.1**	**WP_000821694.1**	**WP_000562692.1**	**Taxonomic ID**	**Genome Assembly Accession**
100.0%	88.03210842453218%	85.04189495352071%	96.99820788530467%	82.0562224347712%	672	GCF_001653775.1

Other Gene ■ RvvA ■ RvvB ■ RvvC ■

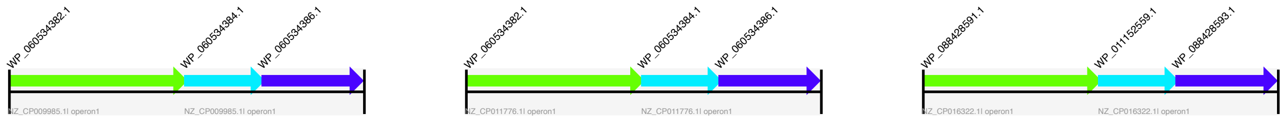

Vibrio_proteolyticus_NBRC_13287_GCF_000467125.1						
**Structural Similarity**	**Average Percent Amino Acid Identity**	**WP_000173586.1**	**WP_000821694.1**	**WP_000562692.1**	**Taxonomic ID**	**Genome Assembly Accession**
100.0%	49.248306619493604%	46.19167717528373%	64.2742621121747%	37.27898057102239%	1219065	GCF_000467125.1

Other Gene | RvvA | RvvB | RvvC

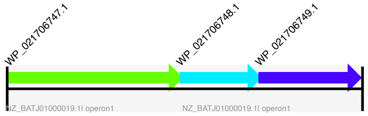

Vibrio_neptunius_GCF_000967495.1						
**Structural Similarity**	**Average Percent Amino Acid Identity**	**WP_000173586.1**	**WP_000821694.1**	**WP_000562692.1**	**Taxonomic ID**	**Genome Assembly Accession**
100.0%	43.17686063121516%	40.28892455858748%	57.198876586394896%	32.042780748663105%	170651	GCF_000967495.1

Other Gene | RvvA | RvvB | RvvC

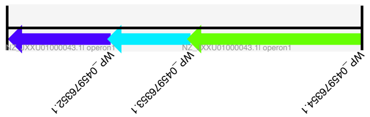

Vibrio_fluvialis_GCF_001558415.2						
**Structural Similarity**	**Average Percent Amino Acid Identity**	**WP_000173586.1**	**WP_000821694.1**	**WP_000562692.1**	**Taxonomic ID**	**Genome Assembly Accession**
100.0%	42.82711105368659%	39.874411302982736%	57.57243909945636%	31.03448275862069%	676	GCF_001558415.2

Other Gene ■ RvvA ■ RvvB ■ RvvC ■

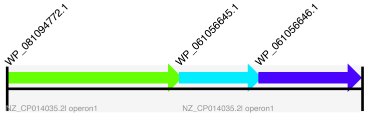

Vibrio_nigripulchritudo_POn4_GCF_001050675.1						
**Structural Similarity**	**Average Percent Amino Acid Identity**	**WP_000173586.1**	**WP_000821694.1**	**WP_000562692.1**	**Taxonomic ID**	**Genome Assembly Accession**
100.0%	42.34594857301396%	37.577639751552795%	58.812860544418065%	30.647345423071016%	1238445	GCF_001050675.1

Other Gene | RvvA | RvvB | RvvC

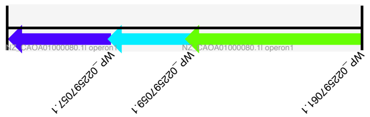

Vibrio_chagasii_GCF_005281815.1						
**Structural Similarity**	**Average Percent Amino Acid Identity**	**WP_000173586.1**	**WP_000821694.1**	**WP_000562692.1**	**Taxonomic ID**	**Genome Assembly Accession**
100.0%	42.621420220493796%	39.03082735083754%	57.47633013052997%	31.35710318011387%	170679	GCF_005281815.1

Other Gene | RvvA | RvvB | RvvC

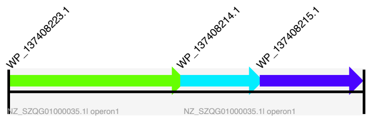

Vibrio_coralliilyticus_GCF_013266665.1						
**Structural Similarity**	**Average Percent Amino Acid Identity**	**WP_000173586.1**	**WP_000821694.1**	**WP_000562692.1**	**Taxonomic ID**	**Genome Assembly Accession**
100.0%	42.99840287521125%	37.871674491392795%	59.391388763664146%	31.732145370576813%	190893	GCF_013266665.1
Other Gene ■ RvvA ■ RvvB ■ RvvC ■						

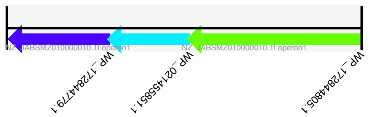

Vibrio_tubiashii_ATCC_19109_GCF_000772105.1						
**Structural Similarity**	**Average Percent Amino Acid Identity**	**WP_000173586.1**	**WP_000821694.1**	**WP_000562692.1**	**Taxonomic ID**	**Genome Assembly Accession**
100.0%	42.14630856696974%	37.76%	58.328680091596254%	30.350245609312964%	1051646	GCF_000772105.1

Other Gene RvvA RvvB RvvC

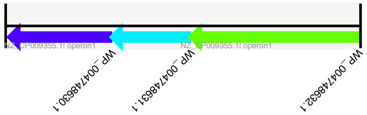

Vibrio_anguillarum_GCF_003390675.1						
**Structural Similarity**	**Average Percent Amino Acid Identity**	**WP_000173586.1**	**WP_000821694.1**	**WP_000562692.1**	**Taxonomic ID**	**Genome Assembly Accession**
100.0%	45.940344425665295%	41.35220125786164%	61.11263149142978%	35.35620052770449%	55601	GCF_003390675.1

Other Gene | RvvA | RvvB | RvvC

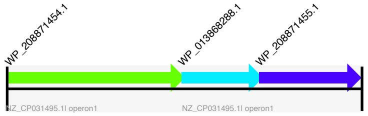

Vibrio_toranzoniae_GCF_900089765.1						
**Structural Similarity**	**Average Percent Amino Acid Identity**	**WP_000173586.1**	**WP_000821694.1**	**WP_000562692.1**	**Taxonomic ID**	**Genome Assembly Accession**
100.0%	42.12931601740823%	38.6321270801608%	58.04005869816209%	29.71576227390181%	1194427	GCF_900089765.1

Other Gene RvvA RvvB RvvC

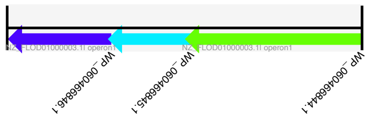

Vibrio_qinghaiensis_GCF_002257545.1						
**Structural Similarity**	**Average Percent Amino Acid Identity**	**WP_000173586.1**	**WP_000821694.1**	**WP_000562692.1**	**Taxonomic ID**	**Genome Assembly Accession**
100.0%	45.73341325086301%	41.61392405063291%	61.11263149142978%	34.473684210526315%	2025808	GCF_002257545.1

Other Gene RvvA RvvB RvvC

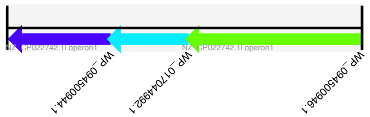

Vibrio_lentus_GCF_002874165.1						
**Structural Similarity**	**Average Percent Amino Acid Identity**	**WP_000173586.1**	**WP_000821694.1**	**WP_000562692.1**	**Taxonomic ID**	**Genome Assembly Accession**
100.0%	41.57417632505247%	38.49593759292206%	56.92067878583431%	29.30591259640103%	136468	GCF_002874165.1
Other Gene ■ RvvA ■ RvvB ■ RvvC ■						

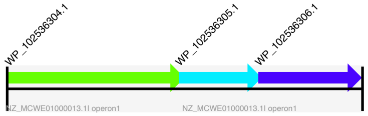

Vibrio_europaeus_GCF_001695575.1						
**Structural Similarity**	**Average Percent Amino Acid Identity**	**WP_000173586.1**	**WP_000821694.1**	**WP_000562692.1**	**Taxonomic ID**	**Genome Assembly Accession**
100.0%	42.01119287292777%	37.36089030206677%	58.10274013018806%	30.569948186528496%	300876	GCF_001695575.1

Other Gene | RvvA | RvvB | RvvC

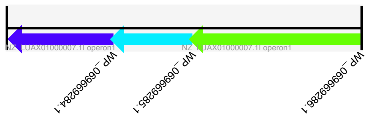

Vibrio_ordalii_FF_167_GCF_000287075.2						
**Structural Similarity**	**Average Percent Amino Acid Identity**	**WP_000173586.1**	**WP_000821694.1**	**WP_000562692.1**	**Taxonomic ID**	**Genome Assembly Accession**
100.0%	45.45567890177895%	40.909090909090914%	60.98426158571962%	34.473684210526315%	617131	GCF_000287075.2

Other Gene | RvvA | RvvB | RvvC

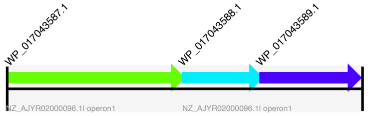

Vibrio_kanaloae_GCF_001995825.2						
**Structural Similarity**	**Average Percent Amino Acid Identity**	**WP_000173586.1**	**WP_000821694.1**	**WP_000562692.1**	**Taxonomic ID**	**Genome Assembly Accession**
100.0%	42.44282181986355%	38.04492742686086%	58.33420091151768%	30.949337121212118%	170673	GCF_001995825.2

Other Gene RvvA RvvB RvvC

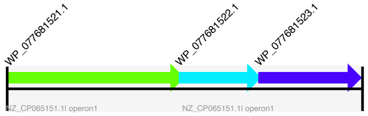

Vibrio_ostreicida_GCF_013074385.2						
**Structural Similarity**	**Average Percent Amino Acid Identity**	**WP_000173586.1**	**WP_000821694.1**	**WP_000562692.1**	**Taxonomic ID**	**Genome Assembly Accession**
100.0%	43.904441657107434%	39.80969989686313%	58.21941454814338%	33.68421052631579%	526588	GCF_013074385.2
Other Gene ■ RvvA ■ RvvB ■ RvvC ■						

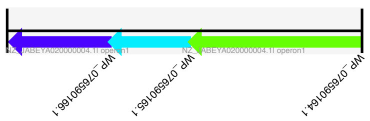

Vibrio_aquimaris_GCF_009363415.1						
**Structural Similarity**	**Average Percent Amino Acid Identity**	**WP_000173586.1**	**WP_000821694.1**	**WP_000562692.1**	**Taxonomic ID**	**Genome Assembly Accession**
100.0%	43.8524652218049%	39.28546356489742%	59.26271850314032%	33.00921359737696%	2587862	GCF_009363415.1

Other Gene | RvvA | RvvB | RvvC

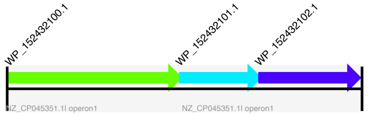

Vibrio_orientalis_CIP_102891_ATCC_33934_GCF_000176235.1						
**Structural Similarity**	**Average Percent Amino Acid Identity**	**WP_000173586.1**	**WP_000821694.1**	**WP_000562692.1**	**Taxonomic ID**	**Genome Assembly Accession**
100.0%	42.42170962481704%	39.22500849881017%	56.62650781019594%	31.413612565445025%	675816	GCF_000176235.1

Other Gene | RvvA | RvvB | RvvC

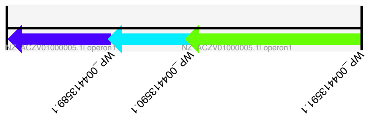

Vibrio_gigantis_GCF_002156475.1						
**Structural Similarity**	**Average Percent Amino Acid Identity**	**WP_000173586.1**	**WP_000821694.1**	**WP_000562692.1**	**Taxonomic ID**	**Genome Assembly Accession**
100.0%	41.57870658094998%	37.80521125711023%	57.087976548566935%	29.84293193717277%	296199	GCF_002156475.1

Other Gene | RvvA | RvvB | RvvC

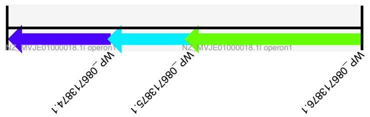

Vibrio_harveyi_GCF_000770115.1						
**Structural Similarity**	**Average Percent Amino Acid Identity**	**WP_000173586.1**	**WP_000821694.1**	**WP_000562692.1**	**Taxonomic ID**	**Genome Assembly Accession**
100.0%	43.40727229094285%	39.28894805177285%	58.374729286171956%	32.55813953488372%	669	GCF_000770115.1

Other Gene | RvvA | RvvB | RvvC

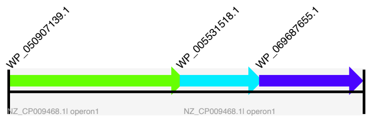

Vibrio_atlanticus_GCF_000091465.1						
**Structural Similarity**	**Average Percent Amino Acid Identity**	**WP_000173586.1**	**WP_000821694.1**	**WP_000562692.1**	**Taxonomic ID**	**Genome Assembly Accession**
100.0%	41.60728463992826%	38.674057240819096%	56.480277497380015%	29.66751918158568%	693153	GCF_000091465.1

Other Gene | RvvA | RvvB | RvvC

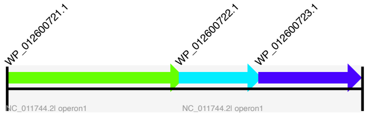

Vibrio_pectenicida_GCF_013114615.1						
**Structural Similarity**	**Average Percent Amino Acid Identity**	**WP_000173586.1**	**WP_000821694.1**	**WP_000562692.1**	**Taxonomic ID**	**Genome Assembly Accession**
100.0%	43.57799362178387%	37.2310646775227%	62.078093492793464%	31.42482269503546%	62763	GCF_013114615.1

Other Gene | RvvA | RvvB | RvvC

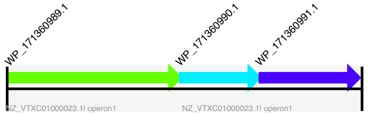

Vibrio_campbellii_GCF_003312585.1						
**Structural Similarity**	**Average Percent Amino Acid Identity**	**WP_000173586.1**	**WP_000821694.1**	**WP_000562692.1**	**Taxonomic ID**	**Genome Assembly Accession**
100.0%	43.549132642221075%	39.31704710010388%	58.34066010490987%	32.98969072164948%	680	GCF_003312585.1

Other Gene ■ RvvA ■ RvvB ■ RvvC ■

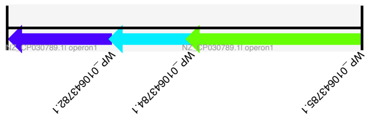

Vibrio_tasmaniensis_GCF_006333845.1						
**Structural Similarity**	**Average Percent Amino Acid Identity**	**WP_000173586.1**	**WP_000821694.1**	**WP_000562692.1**	**Taxonomic ID**	**Genome Assembly Accession**
100.0%	41.920226350979114%	38.22760362501857%	57.143465038308406%	30.38961038961039%	212663	GCF_006333845.1

Other Gene | RvvA | RvvB | RvvC

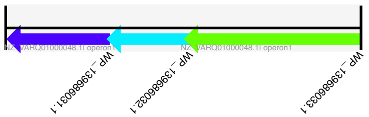

Vibrio_bivalvicida_GCF_001399455.2						
**Structural Similarity**	**Average Percent Amino Acid Identity**	**WP_000173586.1**	**WP_000821694.1**	**WP_000562692.1**	**Taxonomic ID**	**Genome Assembly Accession**
100.0%	42.8087950588969%	38.11051282051282%	57.883386203473364%	32.43248615270452%	1276888	GCF_001399455.2

Other Gene | RvvA | RvvB | RvvC

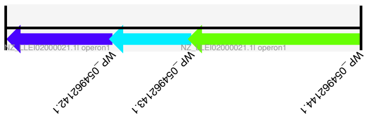

Vibrio_sagamiensis_NBRC_104589_GCF_007990935.1						
**Structural Similarity**	**Average Percent Amino Acid Identity**	**WP_000173586.1**	**WP_000821694.1**	**WP_000562692.1**	**Taxonomic ID**	**Genome Assembly Accession**
100.0%	42.471601115097634%	37.69720777554591%	59.414512664594945%	30.303082905152063%	1219064	GCF_007990935.1

Other Gene ■ RvvA ■ RvvB ■ RvvC ■

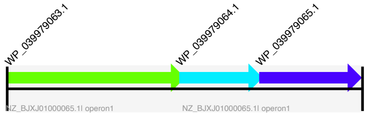

Vibrio_splendidus_GCF_002873735.1						
**Structural Similarity**	**Average Percent Amino Acid Identity**	**WP_000173586.1**	**WP_000821694.1**	**WP_000562692.1**	**Taxonomic ID**	**Genome Assembly Accession**
100.0%	42.201468535783086%	37.7170119421977%	58.077994187344785%	30.809399477806785%	29497	GCF_002873735.1

Other Gene ■ RvvA ■ RvvB ■ RvvC ■

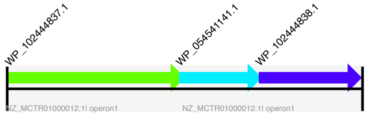

Vibrio_crassostreae_GCF_003751935.1						
**Structural Similarity**	**Average Percent Amino Acid Identity**	**WP_000173586.1**	**WP_000821694.1**	**WP_000562692.1**	**Taxonomic ID**	**Genome Assembly Accession**
100.0%	40.92060090010224%	37.766670999393426%	56.238655016975436%	28.756476683937827%	246167	GCF_003751935.1

Other Gene ■ RvvA ■ RvvB ■ RvvC ■

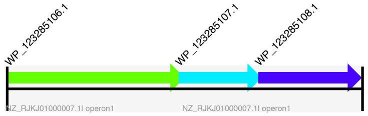

Vibrio_coralliirubri_GCF_900379685.1						
**Structural Similarity**	**Average Percent Amino Acid Identity**	**WP_000173586.1**	**WP_000821694.1**	**WP_000562692.1**	**Taxonomic ID**	**Genome Assembly Accession**
100.0%	42.16925749647894%	38.36119001247703%	57.874862992684584%	30.271719484275216%	1516159	GCF_900379685.1
Other Gene	RvvA	RvvB	RvvC			

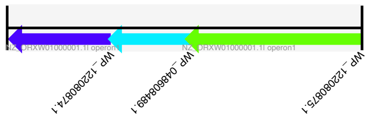

Vibrio_echinoideorum_GCF_004764665.1						
**Structural Similarity**	**Average Percent Amino Acid Identity**	**WP_000173586.1**	**WP_000821694.1**	**WP_000562692.1**	**Taxonomic ID**	**Genome Assembly Accession**
100.0%	41.80081096795897%	37.925736985654865%	57.76093364432023%	29.71576227390181%	2100116	GCF_004764665.1

Other Gene | RvvA | RvvB | RvvC

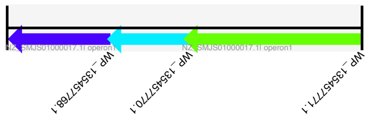

Vibrio_celticus_GCF_002156525.1						
**Structural Similarity**	**Average Percent Amino Acid Identity**	**WP_000173586.1**	**WP_000821694.1**	**WP_000562692.1**	**Taxonomic ID**	**Genome Assembly Accession**
100.0%	42.00184809354237%	38.085348413936565%	57.310546448750976%	30.609649417939572%	446372	GCF_002156525.1

Other Gene ■ RvvA ■ RvvB ■ RvvC ■

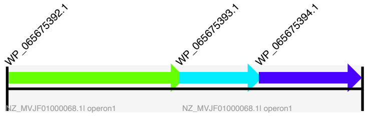

Vibrio_tapetis_subsp_tapetis_GCF_900233005.1						
**Structural Similarity**	**Average Percent Amino Acid Identity**	**WP_000173586.1**	**WP_000821694.1**	**WP_000562692.1**	**Taxonomic ID**	**Genome Assembly Accession**
100.0%	43.59040698208164%	37.019630615689735%	58.64099779808766%	35.110592532467535%	1671868	GCF_900233005.1

Other Gene | RvvA | RvvB | RvvC

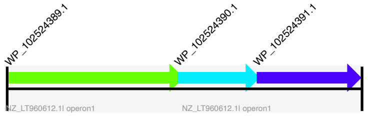

Vibrio_ponticus_GCF_009938225.1						
**Structural Similarity**	**Average Percent Amino Acid Identity**	**WP_000173586.1**	**WP_000821694.1**	**WP_000562692.1**	**Taxonomic ID**	**Genome Assembly Accession**
100.0%	43.096962141040215%	36.53398010920437%	57.60371482455455%	35.1531914893617%	265668	GCF_009938225.1

Other Gene ■ RvvA ■ RvvB ■ RvvC ■

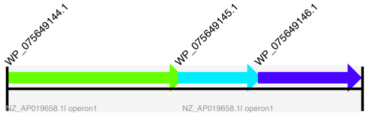

Vibrio_profundi_GCF_005281835.1						
**Structural Similarity**	**Average Percent Amino Acid Identity**	**WP_000173586.1**	**WP_000821694.1**	**WP_000562692.1**	**Taxonomic ID**	**Genome Assembly Accession**
100.0%	40.014296493894655%	36.08087091757387%	54.214457774180524%	29.74756078992959%	1774960	GCF_005281835.1
Other Gene ■ RvvA ■ RvvB ■ RvvC ■						

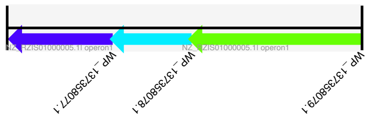

Vibrio_salilacus_GCF_002811245.1						
**Structural Similarity**	**Average Percent Amino Acid Identity**	**WP_000173586.1**	**WP_000821694.1**	**WP_000562692.1**	**Taxonomic ID**	**Genome Assembly Accession**
100.0%	42.5693605517459%	38.40505785833375%	56.89796050576472%	32.40506329113924%	1323749	GCF_002811245.1

Other Gene | RvvA | RvvB | RvvC

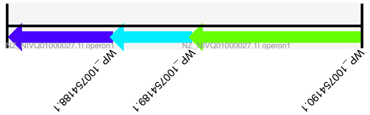

Vibrio_caribbeanicus_ATCC_BAA_2122_GCF_000165125.1						
**Structural Similarity**	**Average Percent Amino Acid Identity**	**WP_000173586.1**	**WP_000821694.1**	**WP_000562692.1**	**Taxonomic ID**	**Genome Assembly Accession**
100.0%	39.76912082497536%	34.18805482839562%	52.37532309224373%	32.74398455428673%	796620	GCF_000165125.1
Other Gene RvvA RvvB RvvC						

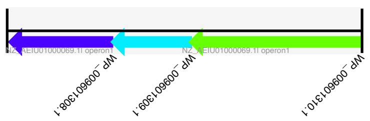

Vibrio_tetraodonis_GCF_003350295.1						
**Structural Similarity**	**Average Percent Amino Acid Identity**	**WP_000173586.1**	**WP_000821694.1**	**WP_000562692.1**	**Taxonomic ID**	**Genome Assembly Accession**
0.0%	10.577663015118501%	31.732989045355502%	0%	0%	2231647	GCF_003350295.1

Other Gene | RvvA | RvvB | RvvC

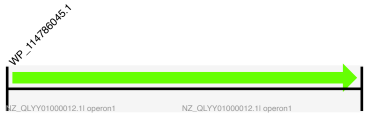

Supplement: S2 Data — (ZIP) [file ppat.1011415.s011.zip › rvvABC_vibrio622_table.pdf]
